# Multiple Imputation with Factor Scores: A Practical Approach for Handling Simultaneous Missingness Across Items in Longitudinal Designs

**DOI:** 10.1080/00273171.2024.2371816

**Published:** 2024-07-12

**Authors:** Yanling Li, Zita Oravecz, Linying Ji, Sy-Miin Chow

**Affiliations:** a Human Development and Family Studies, The Pennsylvania State University, University Park, PA, USA; b Department of Psychology, Montana State University, Bozeman, MT, USA

**Keywords:** Multiple imputation, factor score, missing data, dynamic factor model, emotion

## Abstract

Missingness in intensive longitudinal data triggered by latent factors constitute one type of nonignorable missingness that can generate simultaneous missingness across multiple items on each measurement occasion. To address this issue, we propose a multiple imputation (MI) strategy called MI-FS, which incorporates factor scores, lag/lead variables, and missing data indicators into the imputation model. In the context of process factor analysis (PFA), we conducted a Monte Carlo simulation study to compare the performance of MI-FS to list-wise deletion (LD), MI with manifest variables (MI-MV, which implements MI on both dependent variables and covariates), and partial MI with MVs (PMI-MV, which implements MI on covariates and handles missing dependent variables *via* full-information maximum likelihood) under different conditions. Across conditions, we found MI-based methods overall outperformed the LD; the MI-FS approach yielded lower root mean square errors (RMSEs) and higher coverage rates for auto-regression (AR) parameters compared to MI-MV; and the PMI-MV and MI-MV approaches yielded higher coverage rates for most parameters except AR parameters compared to MI-FS. These approaches were also compared using an empirical example investigating the relationships between negative affect and perceived stress over time. Recommendations on when and how to incorporate factor scores into MI processes were discussed.

## Introduction

Missing data handling is a critical issue in statistical analysis that has continued to garner attention from the statistical, data science, and other related communities in the past decades (Little, 1988; Rubin, 1976). One area that has evidenced some (see, e.g., Ji et al., 2018, 2020) but could benefit from much greater growth is the development of *tailored* tools for handling missingness in intensive longitudinal data (ILD), such as ecological momentary assessments (EMAs) of daily life experiences that capture nuanced changes in behavioral dynamics in naturalistic ecological settings (Bolger & Laurenceau, 2013).

One common characteristic of EMAs and other related ILD is that the constructs of interest are often latent factors (e.g., affect, attitudes, personality traits) that are measured using multiple items. In this scenario, if the reasons for missing data (referred to as missingness mechanisms in this article) are triggered by values of a latent factor, the missingness would likely be observed on *all* items that load on the same factor, yielding pervasive simultaneous missingness that would reduce the efficacy of many missing data handling techniques. For instance, an individual may skip an entire stress-related survey when they feel extremely stressful, in which case the individual’s level on a latent factor, stress, would lead to missingness on all the items. Such simultaneous missingness patterns may generate a nonignorable missingness—or in other words, not missing at random (NMAR)—scenario where the missingness depends on information that is not available in the data set (Rubin, 1976). Such type of missingness, if not appropriately accounted for, would lead to severe biases in estimates and thus misleading inferential results (Ji et al., 2018; Tang et al., 2017). In addition, simultaneous missing data patterns are more challenging to handle compared with missingness in specific, scattered items because in the latter, many existing techniques can still be used to yield reasonable imputed scores for the missing values based on other observed items measuring the same latent construct (Roth et al., 1999). In contrast, few relevant variables can be used to inform imputations when the best items that convey the richest information are themselves missing on the same occasion.

Although many missing data handling techniques exist, few of them have been tailored for handling (simultaneous) missingness in longitudinal data. For instance, one convenient way of handling missing data, the listwise deletion (LD) approach, is known to yield biased estimates in longitudinal settings since the deletion of rows of data alters the time dependencies between observations (Ji et al., 2018). Some alternative approaches, such as full-information maximum likelihood (FIML; Anderson, 1957) and multiple imputation (MI; Rubin, 2004) have been shown to work well under certain missing data mechanisms in longitudinal settings (Ji et al., 2018; Liu & Molenaar, 2014; Rubin, 2004). Among these two approaches, FIML handles missing data by constructing the raw data likelihood function based on the observed data and then optimizing the likelihood function to perform parameter estimation (Arbuckle et al., 1996). This method generally works well for handling missingness in dependent variables provided that the model for dependent variables is correctly specified and the data are missing completely at random (MCAR) or missing at random (MAR). However, since FIML handles missingness in covariates by including the covariates as dependent variables in the model, this approach may not be practically feasible when there are a large number of covariates, especially for categorical covariates and those whose change mechanisms are poorly understood to support reasonable model specification (Ji et al., 2018). In contrast, MI offers considerable flexibility in handling missing data in both dependent variables and covariates, and may benefit from the added information afforded by auxiliary variables, namely, variables that are not included in the model of interest, but are correlated with the variables of interest to some extent or can help explain the missingness mechanisms of certain variables. Therefore, the auxiliary variables, when appropriately chosen, can inform the imputation process and thus reduce biases (Thoemmes & Rose, 2014). In fact, many adaptations of MI methods involved novel selection of auxiliary variables that best suit data characteristics and/or model specifications. For instance, Ji and colleagues proposed two MI strategies (i.e., partial MI and full MI) that were tailored for handling missingness in ILD by including the preceding or following observations of the missing values in the imputation model (Ji et al., 2018). The difference between partial and full MI is that partial MI handles missingness in dependent variables *via* FIML, whereas full MI imputes missing values in dependent variables *via* MI. Despite the growth in the development of tailored tools for handling missingness in ILD, to our best knowledge, there are no MI strategies tailored for handling simultaneous missingess across all manifest variables associated with one common latent variable.

In the present article, we propose an MI strategy called MI with factor scores (MI-FS), which incorporates a series of variables, including factor scores, lag/lead variables (i.e., variables that are lagged/leading in time; for instance, variable $Y_{t-1}\left(Y_{t+1}\right)$ is a lagged (leading) variable of variable $Y_{t}$ with a lag (lead) of one timepoint), and missingness indicators (i.e., a dummy variable indicating whether the data are missing on a specific variable), into the imputation model to respectively account for missingness associated with latent factors, time dependencies between observations in longitudinal data, and missingness mechanisms. Our novel contribution lies in the development and evaluation of ways to leverage information from latent variables through use of longitudinal factor scores in the imputation model to inform the missing data mechanism (i.e., missingness associated with latent factors). Our method also capitalizes on previous useful MI strategies such as including lag/lead variables and missingness indicators in the imputation model. For instance, previous research found that including missingness indicators as auxiliary variables in MI would not introduce bias for parameter estimates under MCAR and MAR, and would reduce bias under NMAR (Beesley et al., 2021; Sperrin & Martin, 2020). The inclusion of missingness indicators in the imputation model allows us to model the relationship between missingness as indicated by missingness indicators and possible variables that may help explain the missingness mechanism, which is essential when missing data follow the NMAR mechanism.

The remainder of the article is organized as follows. We first introduce the empirical data example that motivates our development of the proposed method, with a brief introduction of the model to be fitted to the empirical data. Then we describe the general modeling framework and provide a step-by-step guide on implementing the MI-FS method. Using the process factor analysis model as a basis, we report results from a Monte Carlo simulation study comparing the performance of the MI-FS with that of LD and MI without factor scores under different conditions. We then present an empirical illustration using our empirical data and the four missing data handling methods considered in this study. Finally, we discuss the results, limitations, and future directions.

## Motivating example

**Figure 1.**
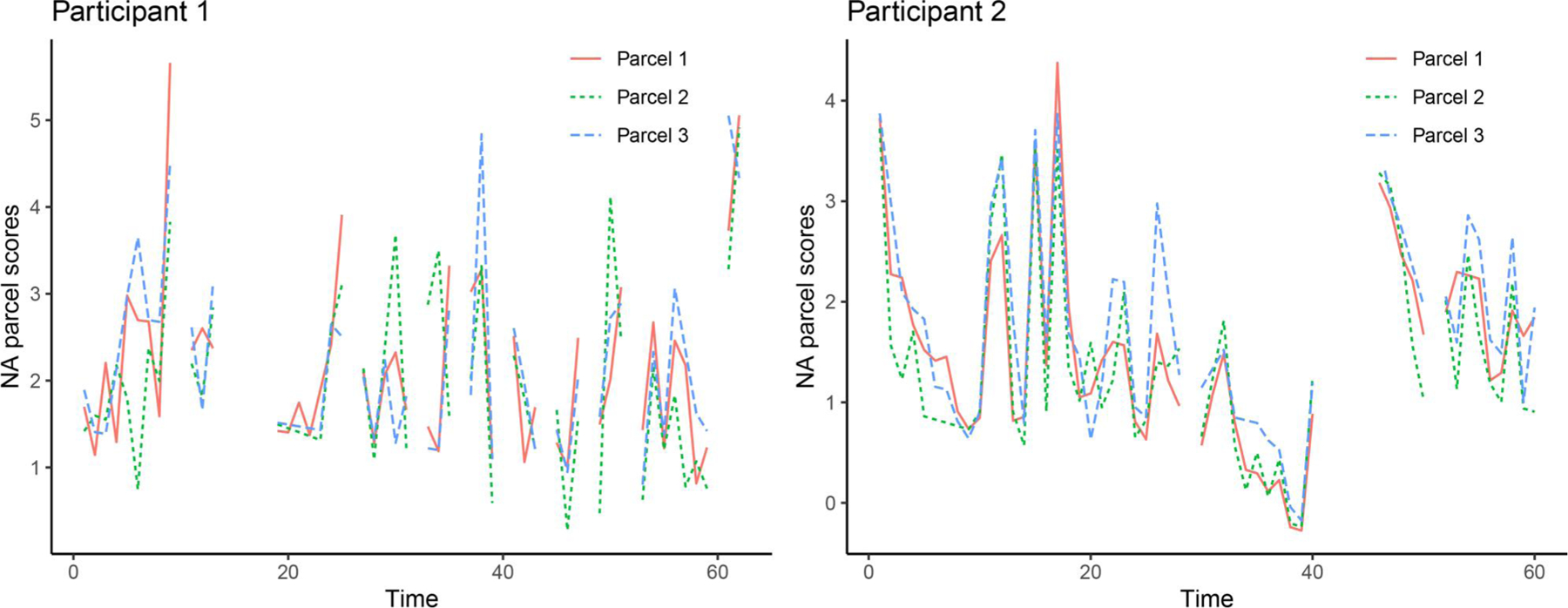
Dynamics of three indicators of negative affect (parcel 1 to 3) for two randomly selected participants.

The motivating example was inspired by the Affective Dynamics and Individual Differences (ADID; Emotions and Dynamic Systems Laboratory, 2010) study where participants were asked to rate their momentary emotions five times a day over a month. All participants provided informed consent prior to participation in the study. In the present article, we were interested in the reciprocal and dynamical linkages between two latent variables, negative affect (NA) and perceived stress (PSS), both of which were measured *via* multiple-item scales over time. Figure 1 shows dynamics of three NA parcels (i.e., item parcels created as indicators for NA; see details in the Empirical Illustration section) for two randomly selected participants. In both plots, the trajectories of three NA parcel scores displayed overall similar patterns since these parcels/indicators were associated with the same underlying latent construct. From the plots we can also see that Participant 1 had a slightly higher baseline level of NA than Participant 2, indicating between-individual differences in affect levels. Importantly, Figure 1 also shows that the missingness in our empirical data was featured by simultaneous missingness across parcels/indicators at a particular timepoint. That is, participants tended to skip all questions in the scale. Therefore, it was reasonable to assume that the missingness was triggered by the latent variable, which motivated us to develop and apply the MI-FS method to address such missingness pattern.

### Process factor analysis model

In this section, we introduce the model to be used in analyzing the empirical data—the process factor analysis (PFA) model and more generally, the dynamic factor analysis (DFA) model (see, e.g., Browne & Nesselroade, 2005; Molenaar, 1985). Conventional factor analysis models are insufficient for studying the intraindividual changes in psychological processes over time, whereas DFA models which can be conceptually understood as a combination of factor analysis and dynamic models allow for simultaneous evaluations of temporal characteristics of latent factors and their relationships with manifest variables over time. Recent decades have seen various applications of DFA models in social and behavioral science fields such as affective processes in dyadic relations (Ferrer & Nesselroade, 2003) and relationships between positive and negative affect (Chow & Zhang, 2013).

In this study, a multilevel PFA model was fitted to the data to investigate (1) intraindividual dynamics of two latent variables, NA and PSS, and their reciprocal linkages over time; (2) effects of other momentary experiences on NA and PSS, such as negative events occurred since the last measurement and personality states (e.g., extraversion, emotional stability, and agreeableness); and (3) individual differences in the baseline levels of NA and PSS, and specifically, how age, gender, and personality traits affected baseline levels. Note that the PFA model considered in this article can be viewed as a special case of DFA models in that the manifest variables are only linked to the latent variables at the same timepoint. Other specifications of DFA models can be found in the relevant literature (e.g., Browne & Nesselroade, 2005).

Based on previous findings on the relationships between NA, PSS, and personality states (Ching et al., 2014; Ebstrup et al., 2011; Leger et al., 2016), we chose to use extraversion, emotional stability, and agreeableness as time-varying covariates because of their purported associations with NA and PSS, while openness and conscientiousness were used as auxiliary variables to inform the MI procedure. As discussed before, individuals may have different affect levels which need to be accounted for in the model fitting. With all these being considered, our final PFA model was defined as follows.


(1) $$\left[\begin{array}{l} {\text{NA}}_{i,t}\\ {\text{PSS}}_{i,t} \end{array}\right]=\left[\begin{array}{l} \mu_{1,i}\\ \mu_{2,i} \end{array}\right]+\left[\begin{array}{ll} a_{1} & b_{1}\\ b_{2} & a_{2} \end{array}\right]\left[\begin{array}{l} {\text{NA}}_{i,t-1}-\mu_{1,i}\\ {\text{PSS}}_{i,t-1}-\mu_{2,i} \end{array}\right]+\left[\begin{array}{llll} c_{1} & d_{1} & e_{1} & f_{1}\\ c_{2} & d_{2} & e_{2} & f_{2} \end{array}\right]\left[\begin{array}{c} {\mathrm{Extrav}}_{i,t}\\ {\mathrm{EmoStab}}_{i,t}\\ {\;\text{Agree}}_{i,t}\\ {\mathrm{NegEvents}}_{i,t} \end{array}\right]+\left[\begin{array}{l} \zeta_{1,i,t}\\ \zeta_{2,i,t} \end{array}\right]$$

(2) $$\left[\begin{array}{l} \mu_{1,i}\\ \mu_{2,i} \end{array}\right]=\left[\begin{array}{l} \gamma_{10}\\ \gamma_{20} \end{array}\right]+\left[\begin{array}{llllll} \gamma_{11} & \gamma_{12} & \gamma_{13} & \gamma_{14} & \gamma_{15} & \gamma_{16}\\ \gamma_{21} & \gamma_{22} & \gamma_{23} & \gamma_{24} & \gamma_{25} & \gamma_{26} \end{array}\right]\left[\begin{array}{c} {\;\text{Gender}}_{i}\\ {\;\text{Age}}_{i}\\ {\;\text{ExtravMean}}_{i}\\ {\;\text{EmoStabMean}}_{i}\\ {\;\text{AgreeMean}}_{i}\\ {\;\text{NegEventsMean}\;}_{i} \end{array}\right]+\left[\begin{array}{l} \psi_{1,i}\\ \psi_{2,i} \end{array}\right]$$

(3) $$\left[\begin{array}{c} {\text{NA\_Ind1}}_{i,t}\\ {\text{NA\_Ind2}}_{i,t}\\ {\text{NA\_Ind3}}_{i,t}\\ {\text{PSS\_Ind4}}_{i,t} \end{array}\right]=\left[\begin{array}{cc} 1 & 0\\ \lambda_{1} & 0\\ \lambda_{2} & 0\\ 0 & 1 \end{array}\right]\left[\begin{array}{l} {\text{NA}}_{i,t}\\ {\text{PSS}}_{i,t} \end{array}\right]+\left[\begin{array}{l} \epsilon_{1,i,t}\\ \epsilon_{2,i,t}\\ \epsilon_{3,i,t}\\ \epsilon_{4,i,t} \end{array}\right]$$

**Figure 2.**
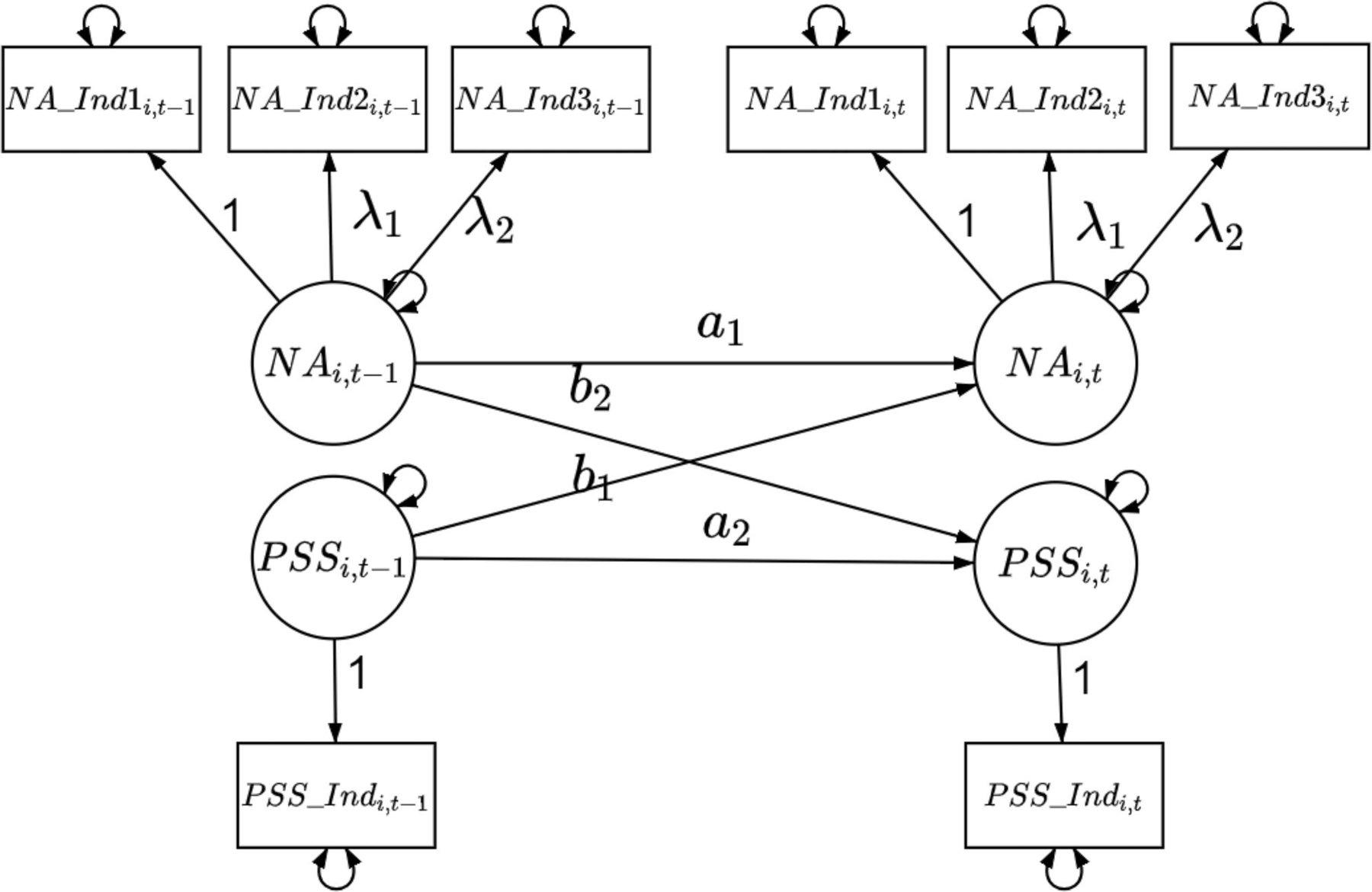
A diagram illustrating the idea of the PFA model presented in Equations (1)–(3). The purpose of this diagram was to visualize the relationships between latent factors of interest, and between latent factors and their corresponding indicators, thus covariates and level-2 predictors were not included. NA: negative affect; PSS: perceived stress.

Here, Equations (1) and (2) jointly defined a *dynamic* model, which was a multilevel vector autoregressive (VAR) model with exogenous variables (i.e., a multilevel VAR-X model) where the dynamics of NA and PSS and their associations with time-varying covariates were modeled in Equation (1). The VAR models are widely used in time series research to capture the dynamic relationships among variables (e.g., NA and PSS in our case) whose past values affect each others’ current values. The graphic representation of such dynamic relationships was presented in the PFA diagram in Figure 2. The exogenous variables (covariates) capture the effects of other measured person- and time-varying (e.g., situational or contextual) variables on the process variables of interest (i.e., NA and PSS). The individual differences in two intercepts (i.e., baseline levels of NA and PSS, $\mu_{1,i}$ and $\mu_{2,i}$) and the effects of person-specific characteristics on baseline levels of NA and PSS were accounted for by the level-2 model in Equation (2). In this empirical example, the random effects were only added for two intercepts to account for possible individual differences in baseline levels of NA and PSS as observed in Figure 1. The random effects were specified as latent variables in the current modeling framework (see, e.g., Chow et al., 2010; Ou, 2018).

Specifically, in the level-1 model in Equation (1), ${\text{NA}}_{i,t}$ and ${\text{PSS}}_{i,t}$ denoted NA and PSS for person i(i=1,…,N) at time $t\left(t=1,\ldots ,T_{i}\right)$, respectively; $a_{1}$ and $a_{2}$ represented auto-regression (AR) parameters which described the relationship between concurrent and lagged values of NA or PSS; and $b_{1}$ and $b_{2}$ represented cross-regression (CR) parameters describing the cross-lagged relationship between NA and PSS. The time-varying covariates, ${\text{Extrav}}_{i,t}$, ${\text{EmoStrb}}_{i,t}$, ${\text{Agree}}_{i,t}$, ${\text{NegEvents}}_{i,t}$, were collected in a vector, with a corresponding matrix of regression coefficients, $c_{1}-f_{2}$. The process noise terms were denoted by $\zeta_{1,i,t}$ and $\zeta_{2,i,t}$, which reflected unmeasured sources that affected the dynamics of ${\text{NA}}_{i,t}$ and ${\text{PSS}}_{i,t}$, respectively, and followed a multivariate normal distribution with zero means and covariance matrix $\sum_{\text{ζ}}$.

In the level-2 model in Equation (2), the person-specific baseline levels of NA and PSS (i.e., $\mu_{1,i,t}$ and $\mu_{2,i,t}$) were respectively regressed on a set of person-specific predictors, including gender, age, and average levels of extraversion, emotional stability, and agree-ableness for each person over the course of the study. The regression coefficients were denoted by γ parameters, with $\gamma_{10}$ and $\gamma_{20}$ being the intercepts, and $\gamma_{11}\text{—}\gamma_{26}$ being the regression weights corresponding to the 6 predictors. We hypothesized that participants’ baseline levels of NA and PSS were negatively related to these personality traits. Finally, the random effects were denoted by $e_{1,i}$ and $e_{2,i}$, which represented person i ’s deviations in the values of $\mu_{1,i}$ and $\mu_{2,i}$ not accounted for by person-specific predictors. These random effects followed a multivariate normal distribution with zero means and covariance matrix $\sum_{\text{ψ}}$, where the random effect variances were denoted by $\sigma_{\psi_{1}}^{2}$ and $\sigma_{\psi_{2}}^{2}$, and the random effect covariance was denoted by $\sigma_{\psi_{12}}$.

Equation (3) defined the *measurement* model which described the factor structures of NA and PSS, which was also shown in Figure 2. The left-hand side of the equation were indicators of NA and PSS (see detailed descriptions about these indicators in the Empirical Illustration section), which were linked to NA and PSS *via* the factor loadings, $\lambda_{1}$ and $\lambda_{2}$. The first factor loading for each factor was fixed at unity for identification purposes. We allowed measurement errors of NA (i.e., $\epsilon_{1,i,t}\;-\;\epsilon_{3,i,t}$) to be correlated to account for common errors associated with the latent factor. We used $\sigma_{\epsilon_{12}}$, $\sigma_{\epsilon_{13}}$, and $\sigma_{\epsilon_{23}}$ to denote the covariance between $\epsilon_{1,i,t}$ and $\epsilon_{2,i,t}$, $\epsilon_{1,i,t}$ and $\epsilon_{3,i,t}$, and $\epsilon_{2,i,t}$ and $\epsilon_{3,i,t}$, respectively.

### General modeling framework

The PFA model presented above can be viewed as a special case of linear discrete-time state-space models. In this section, we introduce this general modeling framework as the general notations will help illustrate the estimation procedure used in our proposed approach. Overall, the general modeling framework is composed of a dynamic model which describes how the latent variables change over time, and a measurement model which relates the observed variables to latent variables at a specific time.

Specifically, the dynamic model exists in a state-space form (Durbin & Koopman, 2001) as

(4) $$\eta_{i,t}=\alpha +F\eta_{i,t-1}+Bx_{i,t}+\zeta_{i,t}\;\zeta_{i,t}∼N\left(0,\Sigma_{\zeta }\right)$$

where $\eta_{i,t}$ is a q-dimensional vector of latent variables for person i at time t, and is linked to their previous values, $\eta_{i,t-1}$ *via* a q×q transition matrix, F; α is a q-dimensional vector of intercepts; B is a matrix of regression weights relating the covariates in $x_{i,t}$ to $\eta_{i,t}$; and $\zeta_{i,t}$ is a q-dimensional vector of process noises following a multivariate normal distribution with zero means and a covariance matrix $\sum_{\text{ζ}}$. Due to the dependencies of $\eta_{i,t}$ on $\eta_{i,t-1}$, the initial conditions for the dynamic processes have to be specified. Here, we specify these initial conditions for $\eta_{i,1}$ to be normally distributed with means ${\text{μ}}_{\eta_{1}}$ and covariance matrix $\sum_{\eta_{1}}$.

In the measurement model, the latent variables at time t, $\eta_{i,t}$, are indicated by a p-dimensional vector of manifest variables at time t, $y_{i,t}$, as follows.

(5) $$y_{i,t}=\tau +\Lambda \eta_{i,t}+Ax_{i,t}+\epsilon_{i,t}\;\epsilon_{i,t}∼N\left(0,\Sigma_{\epsilon }\right)$$

where τ is a p-dimensional vector of intercepts; A is a matrix of regression weights relating the covariates in $x_{i,t}$ to $y_{i,t}$; Λ is a p×q factor loading matrix relating the manifest variables to the latent variables; and $\epsilon_{i,t}$ is a vector of measurement errors assumed to be serially uncorrelated over time and normally distributed with zero means and covariance matrix ${\text{Σ}}_{\epsilon }$.

## Step-by-step guide on implementing MI-FS

Successful use of factor scores in MI processes for ILD are contingent on the availability of a reasonable factor analytic model and corresponding longitudinal factor score estimation approach. In the following section, we group some of these decisions into a five-step description of how to implement the MI-FS approach under the general state-space modeling framework described in Equations (4) and (5), of which our model of interest, the PFA, is a special case.

### Step 1. Constructing factor models

Obtaining factor scores is a critical step in MI-FS. An intuitive approach for obtaining factor scores is factor analysis, such as exploratory factor analysis (EFA) and confirmatory factor analysis (CFA). Below we introduce two types of dynamic CFA models in the context of PFA and VAR models, respectively. Alternative models such as EFA models (see, e.g., Gilbert & Meijer, 2005) can also be potentially used for obtaining factor scores, but we focus on CFA models in the present article.

For longitudinal models that can be expressed in state-space form, such as the PFA utilized in our motivating example, the measurement model of the state-space model (Equation (5)) is itself a factor analytic model. The state-space model thus serves as a rubric for obtaining longitudinal factor scores for imputation purposes. Other cross-sectional factor analytic models can also be specified as special cases of the state-space model (with F in Equation (4) set to be a null matrix). If any information is available to define the dynamics of the implicated variables, a state-space model that includes both the measurement and dynamic models should be utilized whenever possible. For instance, when the PFA model is used for data analysis and missingness occurs in both dependent variables and covariates, one can fit a PFA model without covariates to obtain factor scores using the Kalman filter or Kalman smoother (see more details in Step 2 below)—for instance, excluding the time-varying covariates in Equation (1) from the analysis so that we do not need to handle missingness in covariates. Such omission of covariate effects when they are indeed present in the true model would induce biases, particularly in the dynamic-related parameters (e.g., F and $\sum_{\zeta }$ in Equation (4)). In our simulation study, we verified that these biased factor score estimates may still be informative for the MI procedure by providing information about the dynamics of the latent variables, especially when the missingness is associated with the values of latent variables.

As another example, the VAR model may also be specified as another special case of the state-space model. In this case, the latent variable estimators described in the next step can provide model-implied estimates of the missing observed variables at a particular timepoint, as opposed to just their observed lead or lagged values at other nonmissing timepoints. Alternatively, if missingness in dependent variables is hypothesized to be triggered by a common latent variable, one may fit a CFA model in which the dependent variables in the VAR model are specified as indicators of a common factor. In this case, even though the dependent variables that constitute the VAR processes do not share any theoretically meaningful common factors, their shared missingness mechanisms might constitute a source of covariation that needs to be accounted for. The present study explored this possibility in the context of a Monte Carlo simulation study. In other contexts, an EFA may need to be performed to facilitate the derivation of a heuristic factor structure.

### Step 2. Estimating latent variable values

Once a factor analytic model of choice has been specified, popular factor score estimators, such as the Kalman filter (KF; Kalman, 1960) or the related Kalman smoothers (Ansley & Kohn, 1985; Chow et al., 2010; Dolan & Molenaar, 1991; Oud et al., 1990) can then be applied to obtain the estimates of latent variable values.

Here, we briefly describe the key estimation procedures. More detailed estimation procedures for fitting the model shown in Equations (4) and (5) can be found in Harvey (1990) and Chow et al. (2010). In brief, the KF provides the conditional latent variable estimates for each person at a particular time t based on observed data available up to time t, namely, $E\left(\eta_{i,t}\text{|}\left\{y_{i,j},j=1,\ldots ,t\right\}\right)$, and the corresponding covariance matrix, $Cov\left(\eta_{i,t}\text{|}\left\{y_{i,j},j=1,\ldots ,t\right\}\right)$ In applications where the entire time series of observations is available for estimation purposes, a closely related alternative is a Kalman smoothing technique known as the fixed interval approach, which yields the smoothed latent variable estimates, $E\left(\eta_{i,t}\text{|}\left\{y_{i,j};j=1,\ldots ,T_{i}\right\}\right)$, and the corresponding covariance matrix, $Cov\left(\eta_{i,t}\text{|}\left\{y_{i,j},j=1,\ldots ,T_{i}\right\}\right)$, where $T_{i}$ is the number of observations for person i. This approach has been shown to be equivalent to the well-known regression approach for estimating factor scores (Chow et al., 2010; Dolan & Molenaar, 1991).

Application of the KF and related techniques assumes that the parameter are fixed at their known values. We provide a brief description about ways to obtain these parameter estimates. Under normality assumptions of the measurement and process noise components and linearity of the dynamic and measurement functions (see Equations (4) and (5)), the prediction errors, $y_{i,t}-E\left(y_{i,t}\text{|}\left\{y_{i,j},j=1,\ldots ,t-1\right\}\right)$, which capture the discrepancies between the manifest observations and the predictions implied by the model at time t, are multivariate normally distributed. This yields a log-likelihood function, also known as the *prediction error decomposition* function (Schweppe, 1965), computed using by-products from the KF, that can be optimized to yield estimates of all the time-invariant parameters. This is similar to the FIML parameter estimates which are based on only the available observed variables. In sum, following the specification of a factor analytic model, the KF, the Kalman smoother, and optimization of the prediction error decomposition function can be utilized to yield maximum likelihood point estimates for all the time-invariant parameters, and smoothed estimates of all the latent variables for all individuals and timepoints.

### Step 3. Implementing multiple imputation

The factor scores obtained in Step 2 will be used in MI to inform the missingness mechanism (i.e., missingness associated with latent factors). With MI, missing observations are repeatedly imputed based on prespecified imputation models to yield multiple imputed data sets. One commonly used method for imputing missing values is the fully conditional specification (FCS) method (van Buuren & Oudshoorn, 2000), which can be carried out with the *Multivariate Imputation by Chained Equations R* package (*MICE*; van Buuren & Groothuis-Oudshoorn, 2011). The specific algorithm adopted in *MICE* is called “chained equations” (Raghunathan et al., 2001), which imputes missing values in each variable iteratively, conditional on the observed and imputed values prior to the current imputation.

Assume that we have p dependent variables, $y=\left(y_{1},\ldots ,y_{p}\right)$, and q covariates, $x=\left(x_{1},\ldots ,x_{q}\right)$. The observed parts of y and x are denoted by $y^{obs}=\left(y_{1}^{obs},\ldots ,y_{p}^{obs}\right)$ and $x^{obs}=\left(x_{1}^{obs},\ldots ,x_{q}^{obs}\right)$, respectively. Let z be a vector containing all fully observed auxiliary variables that are hypothesized to be associated with the missingness in y and x; $y_{-j}=\left(y_{1},\ldots ,y_{j-1},y_{j+1},\ldots ,y_{p}\right)$ be the collection of the p−1 variables in y without $y_{j}$, and $x_{-j}=\left(x_{1},\ldots ,x_{j-1},x_{j+1},\ldots ,x_{q}\right)$ be the collection of the q−1 variables in x without $x_{j}$. Then the conditional distribution of $y_{j}$ and $x_{j}$ are $P\left(y_{j}\text{|}\theta_{y_{j}},y_{-j},x,\text{z}\right)$ and $P\left(x_{j}\text{|}\theta_{x_{j}},x_{-j},y,\text{z}\right)$, respectively, where $\theta_{y_{j}}$ and $\theta_{x_{j}}$ are unknown parameters that are specific to the respective conditional distributions. Then starting from a simple draw from observed marginal distributions of $y_{j}$, the tth iteration of the chained equations algorithm will successively draw samples (denoted by $\theta_{y_{j}}^{*\left(t\right)}$, $\theta_{x_{j}}^{*\left(t\right)}$, $y_{j}^{*\left(t\right)}$, and $x_{j}^{*\left(t\right)}$) from the respective conditional distribution of $\theta_{y_{j}}$, $\theta_{x_{j}}$, $y_{j}$, and $x_{j}$. Here, $y_{j}^{*\left(t\right)}$ can be viewed as the imputed values for the jth dependent variable at the tth iteration, and we let $y_{j}^{\left(t\right)}=\left(y_{j}^{obs},y_{j}^{*\left(t\right)}\right)$ represent the jth imputed dependent variable at the tth iteration, which consists of both observed and imputed values. Similarly, we use $x_{j}^{*\left(t\right)}$ to denote the imputed values for the jth covariate at the tth iteration, and $x_{j}^{\left(t\right)}=\left(x_{j}^{obs},x_{j}^{*\left(t\right)}\right)$ to represent the jth imputed covariate.

The process of drawing these samples can be summarized as follows.


$$\begin{array}{cc} \text{Update}\;y_{1}^{*(t)},\ldots ,y_{p}^{*(t)}\text{:} & \text{Update}\;x_{1}^{*(t)},\ldots ,x_{q}^{*(t)}\text{:}\\ \theta_{y_{1}}^{*(t)}∼P\left(\theta_{y_{1}}\text{|}y_{1}^{obs},y_{-1}^{\left(t-1\right)},x^{\left(t-1\right)},z\right) & \theta_{x_{1}}^{*(t)}∼P\left(\theta_{x_{1}}\text{|}x_{1}^{obs},y^{\left(t\right)},x_{-1}^{\left(t-1\right)},z\right)\\ y_{1}^{*(t)}∼P\left(y_{1}\text{|}\theta_{y_{1}}^{*(t)},y_{-1}^{\left(t-1\right)},x^{\left(t-1\right)},z\right) & x_{1}^{*(t)}∼P\left(x_{1}\text{|}\theta_{x_{1}}^{*(t)},y^{\left(t\right)},x_{-1}^{\left(t-1\right)},z\right)\\ \ldots & \ldots \\ \theta_{y_{p}}^{*(t)}∼P\left(\theta_{y_{p}}\text{|}y_{p}^{obs},y_{-p}^{\left(t\right)},x^{\left(t-1\right)},z\right) & \theta_{x_{q}}^{*(t)}∼P\left(\theta_{x_{q}}\text{|}x_{q}^{obs},y^{\left(t\right)},x_{-q}^{\left(t\right)},z\right)\\ y_{p}^{*(t)}∼P\left(y_{p}\text{|}\theta_{y_{p}}^{*(t)},y_{-p}^{\left(t\right)},x^{\left(t-1\right)},z\right) & x_{q}^{*(t)}∼P\left(x_{q}\text{|}\theta_{x_{q}}^{*(t)},y^{\left(t\right)},x_{-q}^{\left(t\right)},z\right) \end{array}$$

Note that the chained equations algorithm is essentially Gibbs sampling, a widely used Markov chain Monte Carlo (MCMC) algorithm, but different from many MCMC algorithms, the chained equations algorithm often converges faster (e.g., convergence can be achieved after 10–20 iterations). Finally, the chained equations algorithm will be implemented multiple times to generate multiple imputed data sets.

With *MICE*, users can specify a univariate imputation model for each incomplete variable, so that at each iteration of the above algorithm, missing values in each variable will be imputed based on the prespecified imputation model (i.e., $P\left(y_{j}\text{|}\theta_{y_{j}},y_{-j},x,\text{z}\right)$ for dependent variables and $P\left(x_{j}\text{|}\theta_{x_{j}},x_{-j},y,\text{z}\right)$ for covariates). In the present study, we use the default imputation models in *MICE* to impute different types of variables—for instance, predictive mean matching (PMM; Little, 1988) for continuous variables and multinomial logistic regression for categorical variables. Specifically, consider a variable c which has some cases with missing data. The PMM method works by first estimating the conditional distribution of c given all observed variables that are hypothesized to be related to the missingness in c, using a predictive model (typically linear regression). Then, for each missing value of c, the algorithm selects an observed value of c that is close to the predicted value. This observed value is used to replace the missing value. This method is particularly useful when dealing with continuous variables, and can produce imputations that preserve the distribution and relationships between variables in the original data set. For missing categorical variables, multinomial logistic regression models are often used to generate the predictive distribution.

The proposed approach considers the following variables to be included in the imputation model: (1) variables in the model of interest, including both dependent variables and covariates; (2) factor scores obtained from Step 2; (3) lags/leads of variables in (1) and (2); (4) missing data indicators; and (5) other auxiliary variables hypothesized to be related to variables of interest and/or missing data mechanisms. Among these variables, the factor scores are important missingness-related variables when the missingness is associated with latent factors; the lagged and leading variables are helpful in capturing the time dependencies between observations; and missing data indicators are important auxiliary variables as evidenced in previous research (Beesley et al., 2021; Sperrin & Martin, 2020).

### Step 4. Fitting models of interest and pooling estimation results

Assume that we obtain m imputed data sets from Step 3, then we can fit our model of interest to each imputed data set to obtain m sets of parameter estimates, which are then pooled following Rubin’s rules (Rubin, 1976) to obtain the final estimation results (i.e., point and standard error estimates, confidence intervals, etc.).

Finally, the MI-FS approach can be implemented using an utility function called *dynr.mi()* in an *R* package called *Dynamic Modeling in R* (*dynr;* Ou et al., 2019), which was designed to handle possibly nonignorable missingness in the dependent variables and/or covariates by performing the MI procedure adapted to ILD with dynamic systems modeling within *dynr*. A more thorough introduction of the general modeling framework and estimation procedures underlying *dynr*, as well as a detailed introduction about *dynr.mi()* can be found in the relevant literature (Chow et al., 2018; Li et al., 2019; Ou et al., 2019).

## Missing data handling approaches considered

The proposed approach was compared with LD (a baseline approach) and two MI strategies without factor scores, referred to as “full MI” and “partial MI” in Ji et al. (2018), and “MI with manifest variables (MI-MV)” and “partial MI with manifest variables (PMI-MV)” in the present article. The four approaches are detailed below, with a brief summary and comparison of these approaches in Table 1.

### LD

The LD approach was designed to mirror common practice of deleting an entire instance (row) of observations with missingness in any variable, whereas the LD approach was implemented in the present study by removing rows with missing entries in covariates, whereas rows with missing entries *only* in the dependent variables were kept in the data set and handled *via* FIML.

### MI-FS

Following the four steps described before, we first obtained factor scores by fitting a PFA model with no covariates. Then we multiply imputed both dependent variables and covariates using an imputation model consisting of (1) variables in the model of interest, including both dependent variables and covariates; (2) factor scores; (3) lags/leads of variables in (1) and (2); (4) missing data indicators; and (5) other auxiliary variables. Lastly, we fitted the PFA model to each imputed data set and pooled estimation results.

### MI-MV

The MI with manifest variables approach was almost identical to the MI-FS, except that factor scores did not need to be estimated and included in the imputation model.

### PMI-MV

Both MI-MV and PMI-MV included dependent variables and covariates in the imputation model. The difference was that, with MI-MV, the imputed dependent variables were used in the following model fitting as if they were completely observed, whereas with PMI-MV, the imputed values of dependent variables were discarded since the missingness in dependent variables was handled *via* FIML.

## Simulation study

The goals of this simulation study were to evaluate: (1) whether and how the incorporation of factor scores into MI affect the estimation accuracy and uncertainty compared to LD and MI without factor scores; (2) the effects of the autocorrelation level of the process on the performance of these approaches; (3) the effects of missingness mechanisms on the performance of these approaches; (4) the role of missingness indicators in data imputation; and (5) the effects of misspecification of factor analytic structure in deriving factor scores for MI purposes. The results will help guide researchers to select missing data handling approaches that are better suited for their study questions, hypotheses about missing data mechanisms, as well as data characteristics.

Specifically, using the PFA model, we simulated data by crossing two factors with two conditions each, yielding four conditions in total. The first factor was the autocorrelation level of the VAR process, which was set to either low or high to investigate the performance of missing data handling approaches when they are applied to longitudinal data with different autocorrelation levels. The second factor was the missingness mechanism, namely, whether missingness was driven by items versus latent factors. Four missing data handling approaches were considered, including LD, MI-FS, MI-MV and PMI-MV. For each of the three MI-based approaches, we also considered MI with and without the inclusion of missingness indicators in the imputation model. Finally, based on simulation results designed to address our first three request questions of interest, we performed targeted simulations within one the four conditions—missing mechanisms driven by latent factors and data with low autocorrelations, to further examine the performance of our proposed method under misspecification of the factor analytic structure. In the following section, we describe the data generating model, followed by details of the two design factors, the four missing data handling approaches considered in this study, as well as a set of summary statistics used to evaluate the estimation performance. The R code for generating complete and missing data as well as implementing different missing data handling approaches can be accessed *via* https://github.com/yanlingli1/MI-with-factor-scores.

### Complete data generation model

The complete data generation model was similar to the PFA model presented in Equations (1)–(3), except that we did not allow the intercepts in the dynamic model to be person-specific and the measurement errors to be correlated, as well as reduced the number of covariates for simplicity purposes.

The dynamic model was specified below, where most notations have been described in the Motivating Example section, reiterated here for clarity with “context–neutral” notations for the dependent variables (i.e., $\eta_{1,i,t}$ and $\eta_{2,i,t}$) and time-varying covariates (i.e., $x_{1,i,t}$, a binary covariate, and $x_{2,i,t}$, a continuous covariate). Specifically, the dynamic model was specified as:

(6) $$\left[\begin{array}{l} \eta_{1,i,t}\\ \eta_{2,i,t} \end{array}\right]=\left[\begin{array}{ll} a_{1} & b_{1}\\ b_{2} & a_{2} \end{array}\right]\left[\begin{array}{l} \eta_{1,i,t-1}\\ \eta_{2,i,t-1} \end{array}\right]+\left[\begin{array}{ll} c_{1} & d_{1}\\ c_{2} & d_{2} \end{array}\right]\left[\begin{array}{l} x_{1,i,t}\\ x_{2,i,t} \end{array}\right]+\left[\begin{array}{l} \zeta_{1,i,t}\\ \zeta_{2,i,t} \end{array}\right],\left[\begin{array}{l} \zeta_{1,i,t}\\ \zeta_{2,i,t} \end{array}\right]∼N\left(0,{\text{Σ}}_{\zeta }=\left[\begin{array}{cc} \sigma_{\zeta_{1}}^{2} & \sigma_{\zeta_{12}}\\ \sigma_{\zeta_{12}} & \sigma_{\zeta_{2}}^{2} \end{array}\right]\right)$$

whereas the measurement model was defined as:

(7) $$\left[\begin{array}{l} y_{1,i,t}\\ y_{2,i,t}\\ y_{3,i,t}\\ y_{4,i,t}\\ y_{5,i,t}\\ y_{6,i,t} \end{array}\right]=\left[\begin{array}{ccc} \mu_{1} & 1 & 0\\ \mu_{2} & \lambda_{1} & 0\\ \mu_{3} & \lambda_{2} & 0\\ \mu_{4} & 0 & 1\\ \mu_{5} & 0 & \lambda_{3}\\ \mu_{6} & 0 & \lambda_{4} \end{array}\right]\left[\begin{array}{c} 1\\ \eta_{1,i,t}\\ \eta_{2,i,t} \end{array}\right]+\left[\begin{array}{c} \epsilon_{1,i,t}\\ \epsilon_{2,i,t}\\ \epsilon_{3,i,t}\\ \epsilon_{4,i,t}\\ \epsilon_{5,i,t}\\ \epsilon_{6,i,t} \end{array}\right],\epsilon_{j,i,t}∼N\left(0,\sigma_{\epsilon_{j}}^{2}\right)$$

in which the manifest variables, $y_{1,i,t}-y_{3,i,t}$, were indicators for $\eta_{1,i,t}$, with $\lambda_{1}$ and $\lambda_{2}$ being the factor loadings; and $y_{4,i,t}-y_{6,i,t}$ were linked to $\eta_{2,i,t}$ *via* factor loadings, $\lambda_{3}$ and $\lambda_{4}$. Note that the first factor loading for each factor was fixed at unity for identification purposes. We also freely estimated the intercepts, $\mu_{1}\;\text{-}\;\mu_{6}$. Each manifest process’s measurement errors were hypothesized to follow a univariate normal distribution.

The complete data set that consisted of 30 participants and 100 timepoints for each participant was simulated based on the PFA model defined in Equations (6) and (7), where the true values of model parameters were set mainly based on estimation results in previous studies (e.g., You et al., 2020). Specifically, in the dynamic model, the true values for $a_{1}$ and $a_{2}$ were set to 0.5 for the low-autocorrelation condition and 0.7 for the high-autocorrelation condition. Under the low-autocorrelation condition, the relationship between a variable’s current value and its previous values was low to moderate, whereas under the high-autocorrelation condition, such relationship was high. These values were specifically selected so the resultant VAR process is stable. A system is said to be stable (Lütkepohl, 2005) when all of its statistical properties, including mean and variance, are constant over time. For the case of the VAR process shown in Equation (4), the process is stable when the roots of the determinant of the matrix I−FB all have moduli greater than unity, where B is the backshift operator such that $B\eta_{t}=\eta_{t-1}$. An AR(1) process is stable when the AR parameter (e.g., $a_{1}$ and $a_{2}$ in Equation (6)) is between −1 and 1 (noninclusive). Processes that are unstable, or close to being unstable, are generally associated with greater estimation challenges, although they also highlight more clearly the impact of using factor scores in the imputation process.

The CR parameters were set as $b_{1}=-0.2$ and $b_{2}=-0.3$; the covariate-related coefficients were specified as $c_{1}=0.3$, $c_{2}=-0.3$, $d_{1}=0.5$, and $d_{2}=-0.4$; and elements in $\sum_{\text{ζ}}$ were set as $\sigma_{\zeta_{1}}^{2}=2$, $\sigma_{\zeta_{12}}=0.5$, and $\sigma_{\zeta_{2}}^{2}=6$. In terms of parameters in the measurement model, the intercepts were set to 3 for $\mu_{1}\;\text{-}\;\mu_{6}$; the factor loadings were set as $\lambda_{1}=\lambda_{3}=2$ and $\lambda_{2}=\lambda_{4}=1$; and measurement error variances were set as 1 for $\sigma_{\epsilon_{1}}^{2}\;\text{-}\;\sigma_{\epsilon_{6}}^{2}$.

### Missing data generation models

To investigate the roles of different missing data mechanisms and implications on the utility of including factor scores in the imputation process, we examined two possible nonignorable missingness mechanisms, namely, when the missingness was triggered by (1) values of individual items (i.e., item-dependent missingness); and (2) values of latent factors (i.e., factor-dependent missingness). The former would generate missingness in scattered items when the values of these items were extreme, whereas the latter would yield simultaneous missingness on all items that loaded on the implicated factor when the values of this factor were extreme. The coefficients in the missing data generation model below were specified to generate about 30% missingness in both dependent variables and covariates. This missingness rate was decided based on the range of percentages of missing data in previous longitudinal studies. For instance, a review article found that among 82 longitudinal studies, the percentage of missing data varied from 10% to 55%, with an average of 14% (Okpara et al., 2022). In addition, this setting also allowed us to compare the proposed method with two existing methods proposed by Ji and colleagues (see details below), who conducted their simulation studies also under a missingness rate of 30%.

### Item-dependent missingness

The missingness associated with individual items was generated based on the missing data generation model below.

(8) $$\text{logit}\left(P\left(R_{x_{j,i,t}}=1\text{|}x_{j,i,t},z_{1,i,t},z_{2,i,t}\right)\right)=\phi_{0}+\phi_{1}z_{1,i,t}+\phi_{2}z_{2,i,t}+\phi_{3}x_{j,i,t},j=1,2$$

(9) $$\text{logit}\left(P\left(R_{y_{j,i,t}}=1\text{|}y_{j,i,t},z_{1,i,t},z_{2,i,t}\right)\right)=\phi_{0}+\phi_{1}z_{1,i,t}+\phi_{2}z_{2,i,t}+\phi_{3}y_{j,i,t},j=1,\ldots ,6$$

where $R_{x_{j,i,t}}$ and $R_{y_{j,i,t}}$ were missingness indicators (1 = missing) for covariates and dependent variables, respectively. $\phi_{1}-\phi_{2}$ were both set to 0.6; $\phi_{3}$ was set to −0.8 for covariates, −0.6 for $y_{1}–y_{3}$, and 0.6 for $y_{4}–y_{6}$; and $\phi_{0}$ was adjusted for each manifest variable and covariate to make sure the final missingness rate for each of them was approximately 30%. The probability of missingness was dependent on (1) two fully observed variables, $z_{1,i,t}$ and $z_{2,i,t}$, simulated from a uniform distribution, $U\left[-3,\;3\right]$ and (2) the variable itself, thus yielding a combination of MAR and NMAR conditions.

Based on the above specifications, the manifest dependent variable, $y_{j,i,t}$ accounted for a relatively substantial portion of the variability in the log odds of missingness in the corresponding dependent variable. Specifically, we calculated R-squared values (McKelvey & Zavoina, 1975) to quantify the extent to which $y_{j,i,t}$ contributed to the prediction of missingness in $y_{j,i,t}$. The contribution of $z_{1,i,t}$ and $z_{2,i,t}$, based on McKelvey and Zavoina’s R-squared, were both around 0.2, while the contribution of $y_{1,i,t}-y_{3,i,t}$ and $y_{4,i,t}-y_{6,i,t}$ were round 0.29 and 0.46, respectively, in Equation (9). Thus, the dependent variable itself was designed to play a slightly higher role than the fully observed covariates in affecting the log odds of missingness in the dependent variable under this item-dependent missingness condition.

After incorporating such item-dependent missingness, each item would have 30% missing entries and it would be rare that all items were missing for the same participant given the nature of the missing data generation model—that is, the missingness in each item depended on this item itself.

### Factor-dependent missingness

Under this condition, missingness in covariates was generated based on the same missing data generation model as specified above (see Equation (8)), whereas missingness in dependent variables was generated based on the following missing data generation models.


(10) $$logit\left(P\left(R_{y_{j,i,t}}=1\text{|}\eta_{1,i,t},z_{1,i,t},z_{2,i,t}\right)\right)=\phi_{0}+\phi_{1}z_{1,i,t}+\phi_{2}z_{2,i,t}+\phi_{3}\eta_{1,i,t},j=1,2,3$$

(11) $$logit\left(P\left(R_{y_{j,i,t}}=1\text{|}\eta_{2,i,t},z_{1,i,t},z_{2,i,t}\right)\right)=\phi_{0}+\phi_{1}z_{1,i,t}+\phi_{2}z_{2,i,t}+\phi_{3}\eta_{2,i,t},\;j=4,5,6$$

The difference between Equation (9) and Equations (10) and (11) was that the probability of missingness in each dependent variable (e.g., $y_{1}\text{—}y_{3}$) was specified to be associated with the corresponding latent variable (e.g., $\eta_{1}$), rather than the dependent variable itself. $\phi_{1}-\phi_{2}$ were both set to 0.6; $\phi_{3}$ was set to −0.6 for $\eta_{1}$ and 0.6 for $\eta_{2}$ to represent two different scenarios—the dependent variables (e.g., $y_{1}\text{—}y_{3}$) were more likely to be missing when the values of the corresponding latent variable (e.g., $\eta_{1}$) were low/high; and $\phi_{0}$ was adjusted to make sure the final missingness rate for each manifest variable was approximately 30%.

Based on McKelvey and Zavoina’s R-squared, the contribution of $z_{1,i,t}$ and $z_{2,i,t}$, were both around 0.2, while the contribution of $\eta_{1,i,t}$ was 0.26 in Equation (10) and 0.39 in Equation (11). Thus, the latent factors were also designed to contribute more than the fully observed covariates in affecting the log odds of missingness in the latent variables under the factor-dependent missingness condition, as the manifest dependent variables were under the item-dependent missingness condition. However, note that missingness in the time-varying covariates was never dependent on the latent factors or manifest dependent variables but just the fully observed auxiliary variables (see Equation (8)).

The factor-dependent missing data model defined in Equations (10) and (11) specifies the same probability of missingness for all indicators of the same latent factor. In other words, when one indicator is missing, all other indicators for the same factor would also be missing. In contrast, the item-dependent missing data model in Equation (9) would not yield such simultaneous missingness in general because the probability of missingness is item-specific. In other words, the simultaneous vs. scattered missing data patterns were direct consequences of the two different missingness mechanisms.

### Implementation of missing data handling approaches

Four missing data handling approaches were implemented, including LD, MI-FS, MI-MV, and PMI-MV. The implementation of MI-FS followed the four steps described before. We first obtained factor scores by fitting the PFA model in Equations (6) and (7) with no covariates. Then we multiply imputed both dependent variables and covariates using an imputation model consisting of (1) six dependent variables $\left(y_{1,i,t}-y_{6,i,t}\right)$ and two covariates ($x_{1,i,t}$ and $x_{2,i,t}$); (2) two factor scores (smoothed estimates of $\eta_{1,i,t}$ and $\eta_{2,i,t}$); (3) lags of variables in (1) and (2); (4) eight indicators for missingness in dependent variables and covariates; and (5) two auxiliary variables associated with missingness mechanisms ($z_{1,i,t}$ and $z_{2,i,t}$). Here, the default imputation models in the *MICE* package were used to impute different types of variables. Specifically, the PMM method (introduced before) was used for continuous variables (e.g., $y_{1,i,t}-y_{6,i,t}$, and $x_{2,i,t}$), and logistic regression was used for categorical variables (e.g., $x_{1,i,t}$). Lastly, we fitted the PFA model defined in Equations (6) and (7) to each imputed data set and pooled estimation results. The implementation of other three approaches can be found in the previous section.

For all MI-based approaches, 5 imputations were implemented with 30 iterations in the MCMC procedure in each imputation. Although more imputations may be needed for more complex models, we found that 5 imputations might be sufficient given the good estimation results (see simulation results). In addition, the number of iterations was determined based on the trace plots generated by the *MICE* package, which displayed the mean and standard deviation of the imputed values against the iteration number for each of the 5 imputations. When the number of iterations was set to 30, the trace lines showed good mixing for all variables without any systematic trends. In addition, based on the $\hat{R}$ values (a summary statistic to assess the convergence across multiple MCMC chains by comparing the within-chain variance to the variance of the pooled draws across multiple chains; Gelman et al., 2013) output by the *dynr.mi()* utility function in dynr, satisfactory convergence (i.e., R<1.1) was achieved for all variables under 30 iterations.

Among these four approaches, we hypothesized that LD would generally yield the worst performance on estimation accuracy because the deletion of rows of data would alter the time dependencies between observations and reduce the sample size available for estimation purposes. Among the three MI-based approaches, given the nature of the hypothesized model (i.e., based on dynamics of factors) and the missingness mechanism (i.e., factor-dependent missingness), we hypothesized that MI-FS would outperform MI-MV under conditions with factor-dependent missingness because the inclusion of factor scores and their lagged values would facilitate recovery of the true temporal relationships among latent variables, provided that the factor score estimates were reasonable approximations to the true factor scores. The relative performance of the MI-FS and PMI-MV was unknown and a focus of the simulation study. The PMI-MV uses FIML to handle missingness in dependent variables, which generally works well when the model is correctly specified, even under some NMAR scenarios. For instance, Ji et al. (2018) examined the performance of PMI-MV and MI-MV in the context of VAR models under the low-autocorrelation condition, and the simulation results showed that regardless of the missingness mechanism (i.e., MCAR, MAR, and NMAR), the PMI-MV approach generally outperformed the MI-MV approach in terms of parameter estimation (Ji et al., 2018). However, they only compared these approaches under the low-autocorrelation condition. The increase in the level of autocorrelations may pose additional challenges to imputation and parameter estimation given that time series with a high level of autocorrelation tends to be close to the unstable range. Hence, an investigation of the performance of these MI strategies with highly autocorrelated data was warranted.

In this simulation study, we compared the performance of these methods with data generated using the PFA model in Equations (6) and (7) across four scenarios (two missing data models × two autocorrelation conditions), with the goal of investigating under what circumstances one method outperforms the other in terms of estimation accuracy and uncertainty.

### Performance measures

For each condition and method, we ran 500 Monte Carlo replications, based on which we calculated summary statistics such as biases, relative biases, standard errors (SEs), Monte Carlo standard errors (MCSEs), and root-mean-square errors (RMSEs), as defined below.

Suppose that θ was the true value of a particular parameter, and the point and standard error estimates of θ in the hth $\left(h=1,\ldots ,H\right)$ replication were ${\hat{\theta }}_{h}$ and $SE_{{\hat{\theta }}_{h}}$, respectively. Let the average of point estimates across H replications be $\bar{\theta }$, then the bias, relative bias, SE, MCSE and RMSE were defined as follows:

(12) $$\text{bias}=\frac{1}{H}\sum_{h=1}^{H}\left({\hat{\theta }}_{h}-\theta \right)\text{,}$$

(13) $$\text{relative}\;\text{bias}=\frac{1}{H}\sum_{h=1}^{H}\frac{{\hat{\theta }}_{h}-\theta }{\theta }\text{,}$$

(14) $$\text{SE}=\frac{1}{H}\sum_{h=1}^{H}SE_{{\hat{\theta }}_{h}},$$

(15) $$\text{MCSE}=\sqrt{\frac{1}{H-1}\sum_{h=1}^{H}{\left({\hat{\theta }}_{h}-\bar{\theta }\right)}^{2}},$$

(16) $$\text{RMSE}=\sqrt{\frac{1}{H}\sum_{h=1}^{H}{\left({\hat{\theta }}_{h}-\theta \right)}^{2}}.$$

Here, the SE represents the average estimated standard error for parameter θ, which was compared to the MCSE, the Monte Carlo or empirical standard error, obtained from the standard deviation of point estimates for θ over H replications. It was expected that the SE and MCSE measures would be close to each other in the simulation study. In addition, to compare the quality of the SE estimates to the “benchmark” SE estimates obtained from the complete data set, we calculated “dSEfulls,” defined as the difference between SEs obtained using any of the four missing data handling methods and SEs obtained based on complete data without missingness. Despite the fact that missing data would typically lead to larger SE estimates compared to complete data, this specific measure allowed us to evaluate the *relative* performance of the four missing data handing methods in terms of the deviation from the SEs based on complete data. That is, the SEs based on complete data were used as “benchmark” estimates. We also calculated power, defined as the proportion of replications whose confidence intervals did not contain 0, and coverage rates, defined as the percentages of replications whose confidence intervals contained the true values. Simulation results with higher power and coverage rates close to the nominal rate of 95% would be considered ideal. Note that the power reported in this simulation study was based on the current specifications of effect sizes and sample sizes.

### Simulation results

**Figure 3.**
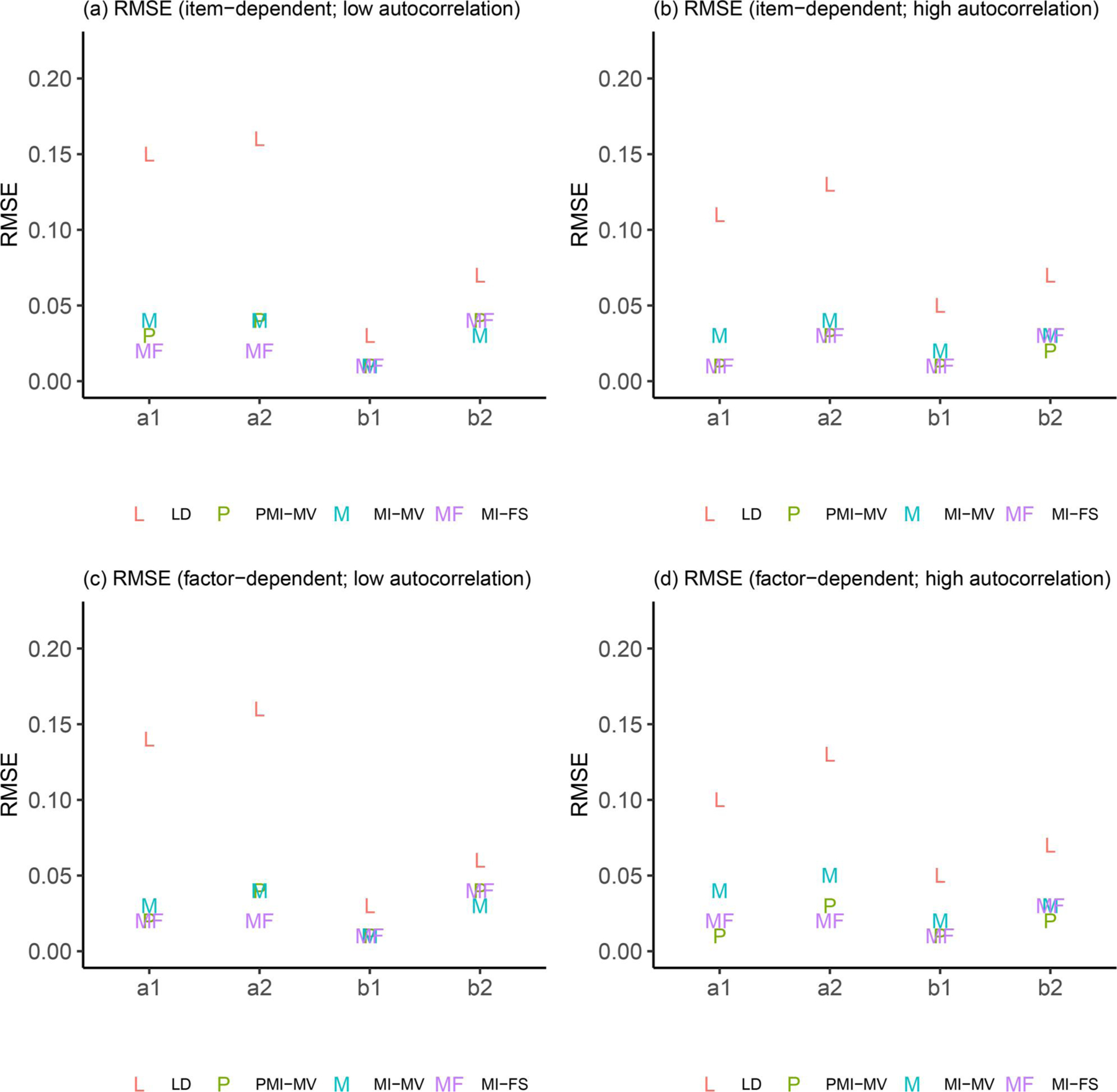
Comparisons of RMSEs across conditions based on 500 Monte Carlo replications (part 1). This plot shows results for AR ($a_{1}$, $a_{2}$) and CR ($b_{1}$, $b_{2}$) parameters in the dynamic model (see Equation (6)). RMSE: root mean square error; LD: listwise deletion; PMI-MV: partial MI with manifest variables; MI-MV: MI with manifest variables; MI-FS: MI with factor scores.

**Figure 4.**
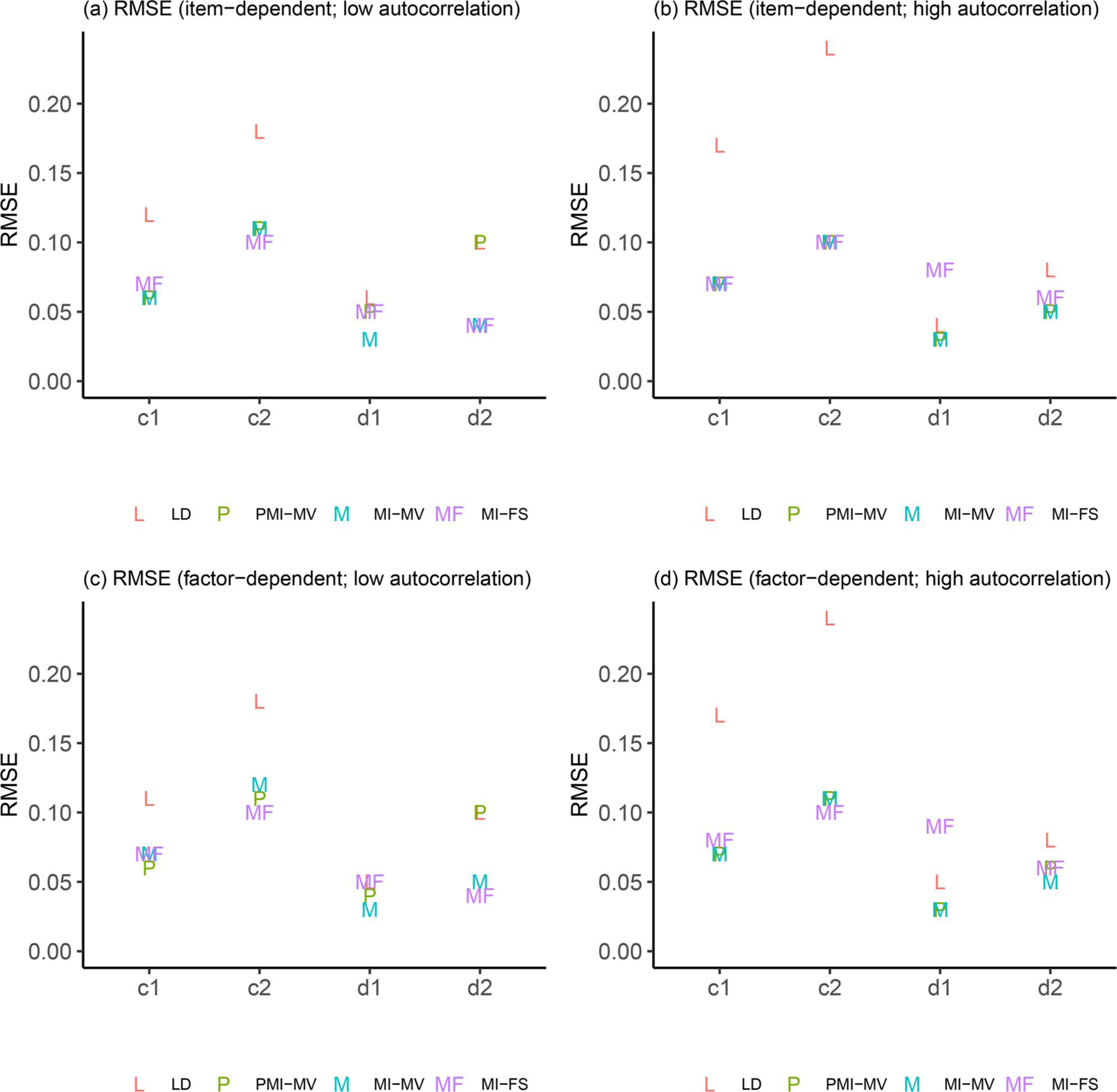
Comparisons of RMSEs across conditions based on 500 Monte Carlo replications (part 2). This plot shows results for coefficients of covariates (i.e., $c_{1}$, $c_{2}$, $d_{1}$, $d_{2}$) in the dynamic model (see Equation (6)). RMSE: root mean square error; LD: listwise deletion; PMI-MV: partial MI with manifest variables; MI-MV: MI with manifest variables; MI-FS: MI with factor scores.

**Figure 5.**
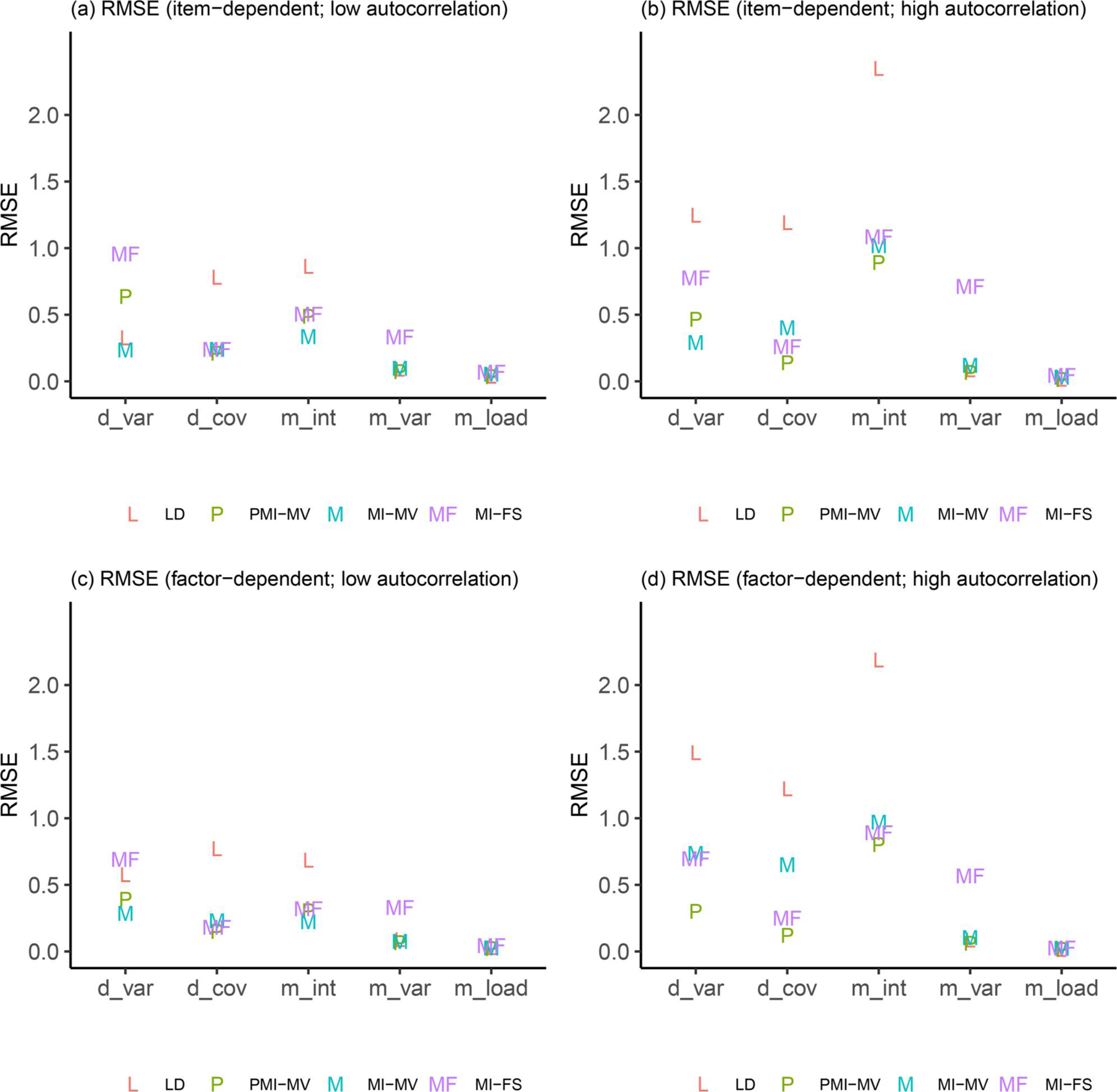
Comparisons of RMSEs across conditions based on 500 Monte Carlo replications (part 3). This plots shows results for the remaining parameters in the model. Specifically, “d_var” and “d_cov”: process noise variances $\left(\sigma_{\zeta_{1}}^{2},\sigma_{\zeta_{2}}^{2}\right)$ and covariance $\left(\sigma_{\zeta_{12}}\right)$ in the dynamic model (see Equation (6)); “m_int”, “m_var” and “m_load”: intercepts $\left(\mu_{1}-\mu_{6}\right)$, measurement error variances $\left(\sigma_{\epsilon_{1}}^{2}-\sigma_{\epsilon_{6}}^{2}\right)$, and factor loadings $\left(\lambda_{1}-\lambda_{4}\right)$ in the measurement model (see Equation (7)). RMSE: root mean square error; LD: listwise deletion; PMI-MV: partial MI with manifest variables; MI-MV: MI with manifest variables; MI-FS: MI with factor scores.

**Figure 6.**
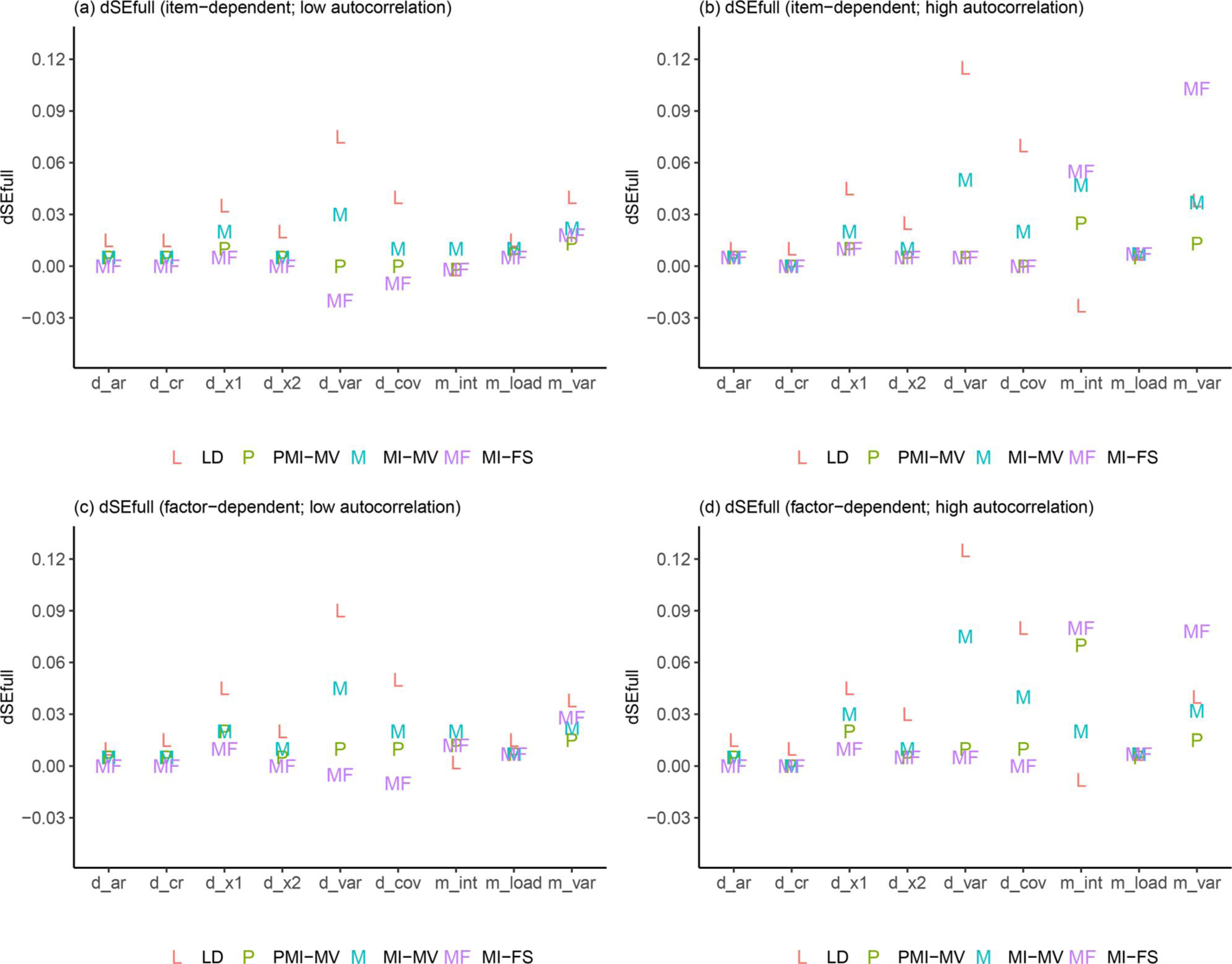
Comparisons of dSEfulls across conditions based on 500 Monte Carlo replications. The dSEfull measure was defined as the difference between SEs obtained using any of the four missing data handling methods and SEs obtained based on complete data without missingness. “d_ar” and “d_cr”: AR ($a_{1}$, $a_{2}$) and CR ($b_{1}$, $b_{2}$) parameters in the dynamic model (see Equation (6)); “d_x1” and “d_x2”: coefficients of covariates (i.e., $c_{1}$, $c_{2}$, $d_{1}$, $d_{2}$) in Equation (6); “d_var” and “d_cov”: process noise variances $\left(\sigma_{\zeta_{1}}^{2},\sigma_{\zeta_{2}}^{2}\right)$ and covariance $\left(\sigma_{\zeta_{12}}\right)$ in Equation (6); “m_int,” “m_var” and “m_load”: intercepts $\left(\mu_{1}-\mu_{6}\right)$, measurement error variances $\left(\sigma_{\epsilon_{1}}^{2}-\sigma_{\epsilon_{6}}^{2}\right)$ and factor loadings $\left(\lambda_{1}-\lambda_{4}\right)$ in the measurement model (see Equation (7)). LD: listwise deletion; PMI-MV: partial MI with manifest variables; MI-MV: MI with manifest variables; MI-FS: MI with factor scores.

**Figure 7.**
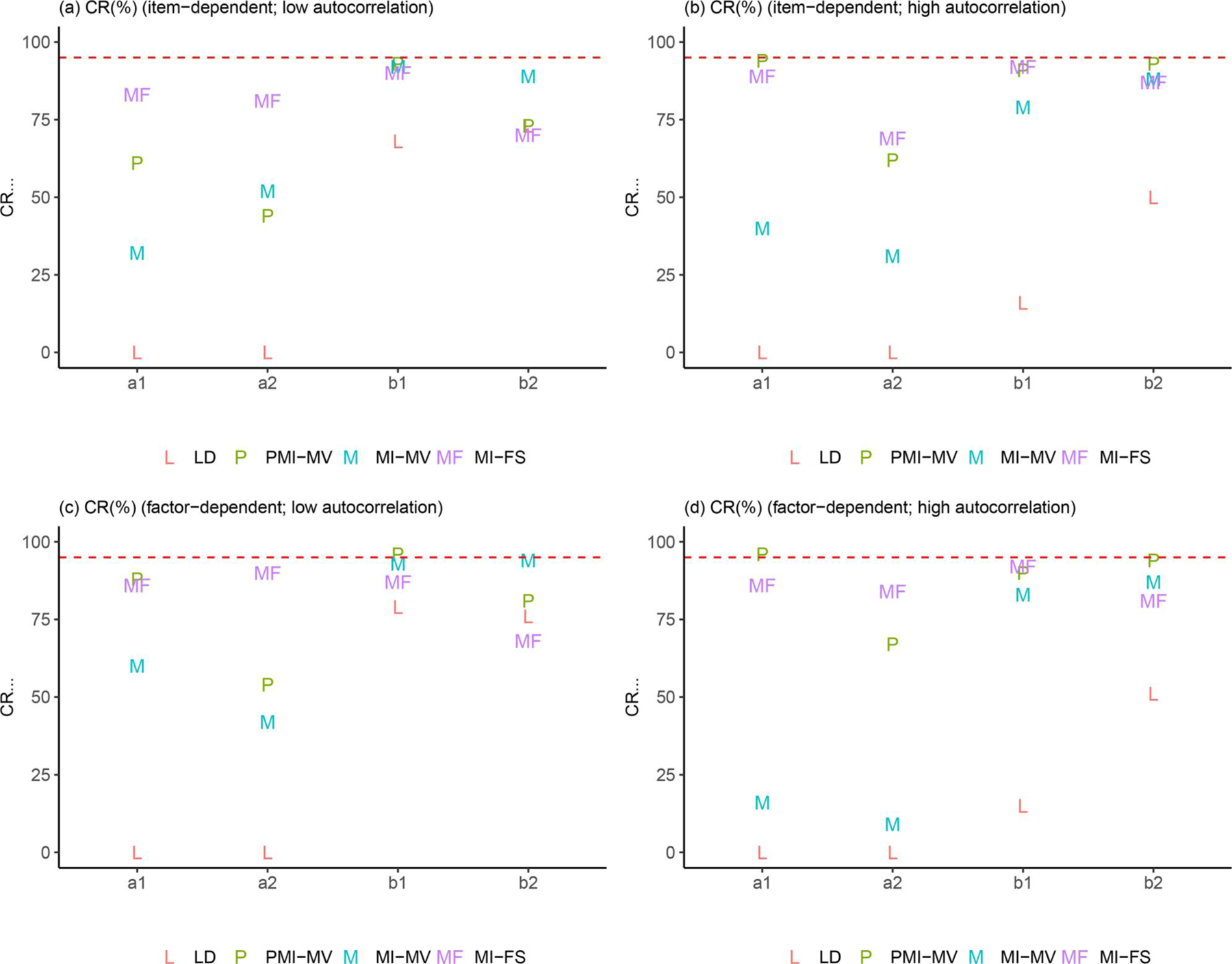
Comparisons of coverage rates across conditions based on 500 Monte Carlo replications. The red dash line represents 95%. This plot shows results for AR ($a_{1}$, $a_{2}$) and CR ($b_{1}$, $b_{2}$) parameters in the dynamic model (see Equation (6)). CR: cross-regression; LD: listwise deletion; PMI-MV: partial MI with manifest variables; MI-MV: MI with manifest variables; MI-FS: MI with factor scores.

**Figure 8.**
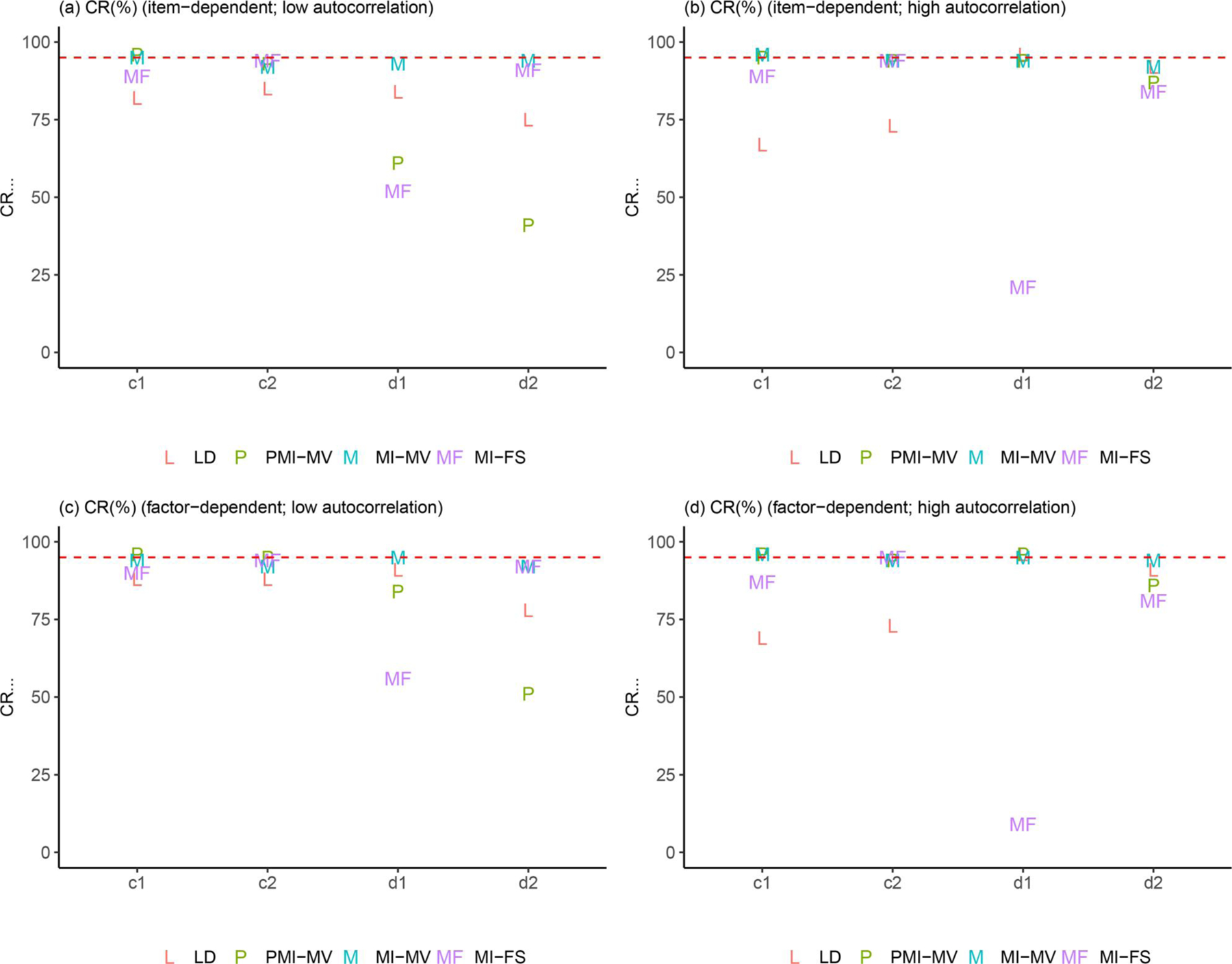
Comparisons of coverage rates across conditions based on 500 Monte Carlo replications. The red dash line represents 95%. This plot shows results for coefficients of covariates (i.e., $c_{1}$, $c_{2}$, $d_{1}$, $d_{2}$) in the dynamic model (see Equation (6)). CR: cross-regression; LD: listwise deletion; PMI-MV: partial MI with manifest variables; MI-MV: MI with manifest variables; MI-FS: MI with factor scores.

**Figure 9.**
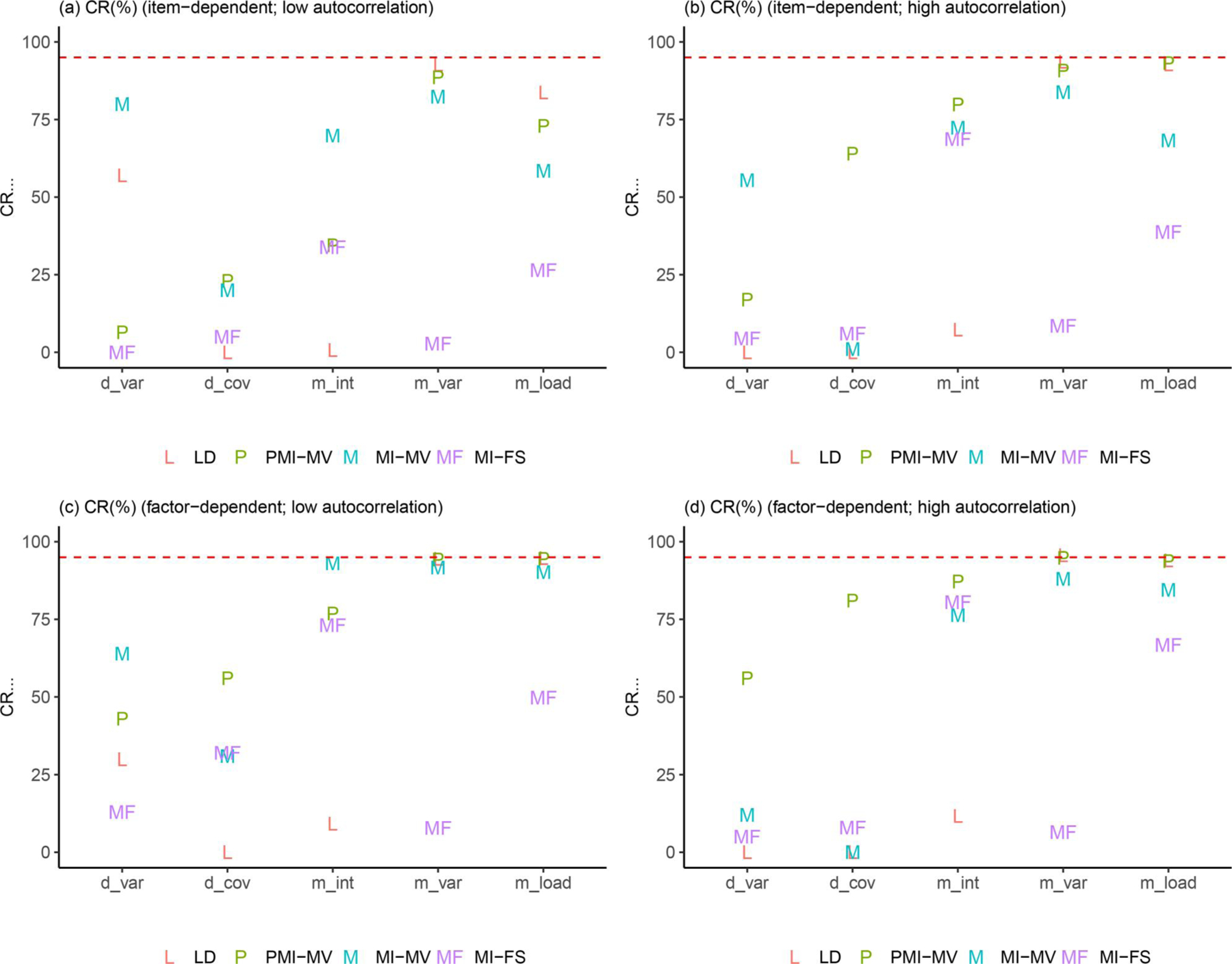
Comparisons of coverage rates across conditions based on 500 Monte Carlo replications. The red dash line represents 95%. This plots shows results for the remaining parameters in the model. Specifically, “d_var” and “d_cov”: process noise variances $\left(\sigma_{\zeta_{1}}^{2},\sigma_{\zeta_{2}}^{2}\right)$ and covariance $\left(\sigma_{\zeta_{12}}\right)$ in the dynamic model (see Equation (6)); “m_int,” “m_var” and “m_load”: intercepts $\left(\mu_{1}-\mu_{6}\right)$, measurement error variances $\left(\sigma_{\epsilon_{1}}^{2}-\sigma_{\epsilon_{6}}^{2}\right)$, and factor loadings $\left(\lambda_{1}-\lambda_{4}\right)$ in the measurement model (see Equation (7)). CR: cross-regression; LD: listwise deletion; PMI-MV: partial MI with manifest variables; MI-MV: MI with manifest variables; MI-FS: MI with factor scores.

Due to space limits, we focus in this section on three summary statistics—RMSEs, dSEfulls, and coverage rates. Other summary statistics (e.g., biases, SEs, MCSEs, and power) can be found in the full simulation results, as summarized in Tables S1–S26 in the Supplementary Material. Across all simulation conditions, we verified that the SE and MCSE measures were relatively close to each other. Since parameters in the dynamic model were of key interest, we presented results for most parameters in the dynamic model but aggregated other parameters by parameter type and then calculated the average of each summary statistic for parameters within the same group (see details about parameter groups in Figures 3–9).

In the following discussion of simulation results, we first described results under the low-autocorrelation condition because this autocorrelation level mirrored the range of autocorrelations reported in most published studies, and then focused on comparisons between different simulation conditions to provide a more thorough comparison. We then discussed the role of missing data indicators, the effects of misspecification of the factor analytic structure, and other findings. Finally, we provided our recommendations for the selection of missing data handling methods in different scenarios.

### Effects of incorporating factor scores into MI (focusing on the low-autocorrelation condition)

This subsection focused on results under the low-autocorrelation condition. First, comparisons of RMSEs across the four methods (see Figures 3–5) showed that MI-based methods (i.e., MI-FS, MI-MV and PMI-MV) substantially outperformed LD on most parameters except for factor loadings, variance parameters (e.g., process noise variances, measurement error variances), and one covariate-related coefficient in the dynamic model (see “d1” in Figure 4). The worse performance of LD on most parameters in the dynamic model was expected since the deletion of rows of data altered the time dependencies between observations.

Focusing next on the three MI-based methods, the simulation results highlighted the advantage of the MI-FS over MI-MV in recovering AR parameters (see “a1” and “a2” in Figure 3), suggesting that the inclusion of factor scores in imputation models did help capture the time-lagged relationship among latent factors and the missingness mechanism. Compared with PMI-MV, MI-FS yielded smaller RMSEs for $a_{2}$ and comparable RMSEs for $a_{1}$ under the low-autocorrelation condition. The difference in the relative performance on $a_{1}$ and $a_{2}$ might be due to different variability levels of $\eta_{1,i,t}$ and $\eta_{2,i,t}$. In fact, in this low-autocorrelation condition, although the autocorrelation level was set to be the same (i.e., 0.5) for $\eta_{1,i,t}$ and $\eta_{2,i,t}$, $\eta_{2,i,t}$ was characterized by higher variability due to its larger process noise variances. Typically, it would be harder to recover $a_{2}$ than $a_{1}$ in this scenario. These results suggested that under the low-autocorrelation condition, MI-FS might be more robust to high-variability time series.

In term of CR parameters, the three MI-based methods yielded almost identical results on RMSEs (see “b1” and “b2” in Figure 3). By contrast, comparisons on the coefficients of time-varying covariates (see Figure 4) differed between low- and high-autocorrelation conditions and will be discussed in detail in the following subsection. Note that the larger RMSEs (i.e., close to 0.1) for coefficients of the binary time-varying covariate (i.e., “c1” and “c2”) indicated more difficulty in recovering this type of parameters than other dynamic model parameters.

Despite the promising use of MI-FS in recovering dynamics of latent factors, we found that the MI-FS yielded worse performance than MI-MV and PMI-MV on variance parameters, including both process noise variances and measurement error variances (see Figure 5). The RMSEs for these parameters under MI-FS were twice as high as under MI-MV or PMI-MV. Further inspection revealed that the MI-FS tended to underestimate process noise variances, which might be due to some losses in data variability at the item level when factor scores were included to generate imputed values. Also note that a model without covariates was used to obtain factor scores, which might also lead to biased process noise variance estimates. By contrast, there were no systematic under/overestimates of measurement error variances under the MI-FS.

Comparisons of dSEfulls are displayed in Figure 6. Similar to comparisons of RMSEs, MI-based methods substantially outperformed LD on dynamic model parameters because the deletion of records with missing covariates reduced the sample size and led to larger SE estimates. Across all methods, the MI-FS led to the least biased SE estimates for most parameters, using the complete data set as a “benchmark.” The only exceptions resided in the intercept and error variance parameters in the measurement model, which was reasonable given the biased estimates of these parameters, which were usually associated with more estimation uncertainty. Note that for process noise variance and covariance parameters, the MI-FS tended to underestimate their SEs, compared with SE estimates obtained based on the complete data (see negative dSEfull values in Figure 6). As mentioned before, the MI-FS tended to underestimate these parameters, probably due to the reduced data variability—specifically, measurement noises—carried by the factor scores used for MI purposes, and such underestimation might in turn lead to underestimated SEs associated with these parameters.

Figures 7–9 provide some clarifications on the extent to which the coverage rates met the nominal levels of coverage probability (e.g., 95%). Under the low-autocorrelation condition, the MI-FS yielded the highest coverage rates on AR parameters but relatively low coverage rates (below 50%) on factor loadings and variance and covariance parameters. The worse performance on process noise variances and covariance was due to smaller SE estimates, more biased point estimates (discussed before), or both, while the worse performance on factor loadings and error variance parameters in the measurement model was mainly resulted from more biased point estimates. By contrast, MI-MV and PMI-MV were generally characterized by comparable or higher coverage rates than the MI-FS on all but the AR parameters, partially due to larger SE estimates and thus larger confidence intervals generated by these MI with manifest variable approaches.

In sum, under the low-autocorrelation condition, compared to other MI methods, the MI-FS yielded less to comparable biases for AR and CR parameters but more biases in variance parameters. The comparison of coverage rates showed lower coverage rates under MI-FS for most parameters except for AR parameters, which was partially due to the smaller SE estimates compared to other MI methods. It should be noted that all these simulation results were discussed based on the PFA model in Equations (6) and (7). Also note that the performance of the MI-FS method relies heavily on the specification of the factor analytic model. The effects of misspecification will be discussed later.

### Effects of autocorrelation levels

Different strengths of the AR parameters could alter the levels of stability of a system’s dynamics as well as its signal-to-noise ratio, thus affecting the consequences of including factor scores in the MI process. By comparing RMSEs between low- and high-autocorrelation conditions, we found MI-based methods were more robust to the change of levels of autocorrelation in the data set, whereas LD generally yielded relatively worse estimation results under the high-autocorrelation condition. In addition, the intercepts of dependent variables (see “m_int” in Figure 5) were harder to recover under the high-autocorrelation condition regardless of the method used (e.g., RMSEs were close to 1 under MI-based approaches and above 2 under the LD approach), which was expected because there were more instances of time series close to the unstable range under this situation.

In terms of comparisons across MI-based methods, the low- and high-autocorrelation conditions led to similar results in terms of the relative performance of these MI approaches, except for comparisons between MI-FS and PMI-MV. First, compared with PMI-MV, the MI-FS yielded smaller RMSEs and higher coverage rates on AR parameters, especially $a_{2}$, under the low-autocorrelation condition but comparable RMSEs and coverage rates under the high-autocorrelation condition. That is, the MI-FS lost its advantage over PMI-MV in recovering AR parameters in the high-autocorrelation scenario. Second, the MI-FS overall outperformed the PMI-MV on the coefficients of the second time-varying covariate (i.e., $x_{2,i,t}$) under the low-autocorrelation condition, but the reverse was observed under the high-autocorrelation condition (see “d1” and “d2” in Figures 4 and 8). These might be due to the increased difficulty in imputation and thus more uncertainty around imputed values in this high-autocorrelation scenario. Since MI-FS and PMI-MV handle missingness in dependent variables differently, with MI-FS using MI while PMI-MV using FIML, a possible reason for the reduced performance of the MI-FS would be that under the high-autocorrelation condition, the MI approach might generate some imputed values that rendered the corresponding time series unstable. More specifically, the default imputation model in the *MICE* package (e.g., see the PMM method introduced before) was used to perform MI without regard to what the true data generation model was, so imputations were basically performed without any stability constraints. In contrast, the PMI-MV approach, through use of the FIML, propagated forward in time model-implied values based on the correctly specified dynamic model (i.e., PFA) at the values of the parameter estimates for each iteration. Thus, when the parameter values are in the stability range (which can be accomplished through constraints on the time series parameters during the optimization process), this approach would yield stable (in contrast to unstable, with increasing variability) model-implied latent variable values, unlike alternatives such as MI-FS and MI-MV which impute with much less constraints and more extraneous (sometimes noisy) information.

### Effects of missingness mechanisms

In addition to autocorrelation levels, missingness mechanisms may also affect the (relative) performance of different missing data handling approaches. First, across all methods, we observed substantial increases in coverage rates on a set of parameters from item-dependent missingness to factor-dependent missingness, under the low-autocorrelation condition (see, e.g., “d_cov,” “m_int,” and “m_load” in Figure 9(a) and (c)). Such increase in coverage rates was mainly related to the larger SE estimates (i.e., larger confidence intervals) of these parameters.

Second, comparisons across methods did not change substantially under two different missing data mechanisms, except for more notable differences between MI-FS and MI-MV. Specifically, in terms of RMSEs on AR parameters and the process noise covariance, the discrepancy between MI-FS and MI-MV was more notable when the missingness was associated with latent factors and the autocorrelation level was high (see Figure 3(d) and Figure 5(d)). Such notable improvement highlighted the power of using factor scores in the imputation, especially when the missing data mechanism was related to latent factors and the autocorrelation level was high.

### The role of missing data indicators

To verify the role of missing data indicators in MI, we replicated the simulation without including missingness indicators in the implementation of MI-MV, PMI-MV, and MI-FS. As a result, the performance of PMI-MV and MI-FS remained similar, but the performance of MI-MV changed dramatically. That is, MI-MV could only yield comparable performance to other MI-based methods when missingness indicators were included in the imputation model. Otherwise, it would produce much larger biases in most parameters, some of which were even close to biases under LD. The results suggested that if MIs were to be used, missingness indicators should be selected as critical auxiliary variables under NMAR missingness, unless other variables closely related to the missingness have already been included in the imputation model, such as the inclusion of factor scores when missingness was associated with latent factors. However, it should be noted that adding too many missing data indicators as auxiliary variables may cause convergence problems, so it is highly recommended to examine the convergence of the MI algorithm. As mentioned before, both the trace plots generated by *MICE* and the diagnostic $\hat{R}$ plots generated by the *dynr.mi()* function can be leveraged to examine the convergence issue. The *MICE* package also provides logged events warnings which may arise from a variety of issues such as collinearity.

## Effects of misspecification of the factor analytic structure

In our main simulation study, the estimated factor scores obtained by fitting a model without covariates were highly correlated with the true latent factors (e.g., correlations higher than 0.9), indicating a relatively good recovery of the latent factors. We also conducted a small simulation study to examine the performance of MI-FS under more misspecified factor analytic models, where we focused on one of the four conditions (i.e., factor-dependent missingness and low autocorrelation). The data were generated based on a tri-VAR model where the missingness in three manifest/observed variables was dependent on a common factor, the values of which were generated as the composite scores of the three manifest variables. Then we fitted a single factor CFA model, a more misspecified factor analytic model compared to the PFA scenario, to obtain the factor scores. Results showed that PMI-MV performed the best in recovering parameters, followed by MI-FS and MI-MV, which yielded similar results. The comparison between MI-FS and MI-MV indicated that even when the dynamics of the underlying processes unfold at the item as opposed to factor level, the inclusion of factor scores in the MI process did not help improve, but also did not induce notably greater biases in the estimation process. In contrast, including factor scores in the MI process greatly improved model estimation properties when the underlying dynamics were driven by latent factors, as in the PFA model. However, given the better performance of PMI-MV, the use of MI-FS may not be necessary in this specific scenario. Therefore, inclusion of factor scores in the MI process helps more in some scenarios than others, and we recommend that the usefulness of incorporating factor scores into MI be examined *via* sensitivity analysis by comparing estimation results with alternative approaches not using factor scores.

### Other remarks

First, the simulation results showed that the MI-FS would generally outperform MI-MV in recovering the temporal relationships between latent variables (provided that the factor scores were reasonable estimates of the true latent factor scores), suggesting that although the observed indicators contained information about the latent factors, just including observed indicators might not be sufficient to provide critical information in the MI process. In fact, this point can also be understood analytically. Specifically, under the hypothesized model in Equations (4) and (5), the conditional distribution of the observed indicators is $y_{i,t}\text{|}\eta_{i,t}∼N\left(\tau +\Lambda \eta_{i,t}+Ax_{i,t},{\text{Σ}}_{\epsilon }\right)$. Thus, information on latent factors, $\eta_{i,t}$, (e.g., factor scores) should be included in the imputation process to facilitate imputation of the observed indicators, $y_{i,t}$. Under factor-dependent missingness (as generated e.g., with Equations (10) and (11)), good estimates of latent factors could further help recover the true missingness mechanism.

On the contrary, if manifest variables were not included in MI—that is, when imputing a specific manifest variable $\left(y_{j}\right)$, other manifest variables $\left(y_{-j}\right)$ would not be included in the imputation model, then the variables used in the imputation model would contain no information about the measurement errors and thus parameters such as measurement error variances are expected to be more biased. To validate our analytically driven conjectures, we tested the performance of this approach under one simulation condition (i.e., high-autocorrelation and factor-dependent missingness) by running a small simulation (100 replications). Detailed simulation results can be found in Table S17 in the Supplementary Material. We found that although estimates of the dynamic model (see Equation (6)) were satisfactory, estimates of parameters in the measurement model (e.g., factor loadings and measurement error variances in Equation (7)) were more biased with this approach compared with either MI-FS or MI-MV. In sum, the comparisons between these three approaches (i.e., MI-FS, MI-MV, and MI with only factor scores) indicated that both manifest variables (or observed indicators) and factor scores played critical roles in the MI process.

Second, to make sure that the simultaneous vs. scattered missing data patterns did not confound the comparisons across missing data handling approaches, we conducted a small simulation study (under the low-autocorrelation condition) by considering an alternative missingness generation scenario in which we defined a fully observed location indicator variable, which took the value of either 0 or 1, with 1 marking data locations for scattered/simultaneous missingness. For instance, under the simultaneous missingness scenario, we had an indicator for simultaneous missingness in $y_{1}$, $y_{2}$, and $y_{3}$, denoted as $R_{\eta_{1}}$. If $R_{\eta_{1}}=1$ for a specific case, then we would set the corresponding cases of $y_{1}$, $y_{2}$, and $y_{3}$ to missing values (similar relationships between $R_{\eta_{2}}$ and $y_{4}$, $y_{5}$, and $y_{6}$). In contrast, in the scattered missingness scenario, we used distinct missing location indicators for $y_{1}$, $y_{2}$, and $y_{3}$, denoted as $R_{y_{1}}$, $R_{y_{2}}$, and $R_{y_{3}}$. If $R_{y_{1}}=1$, we would set the corresponding case of $y_{1}$ but not that of the remaining ys to be missing. These missing data locations were determined randomly, and we included $R_{\eta_{1}}$ and $R_{\eta_{2}}$ as auxiliary variables under simultaneous missingness, and $R_{y_{1}}$, $R_{y_{2}}$, and $R_{y_{3}}$ as auxiliary variables under scattered missingness. This led to two new alternative MAR scenarios that helped to clarify potential confounds due to simultaneous vs. scattered missingness. The simulation results can be found in Tables S18–S23 in the Supplemental Material. We found comparable estimation results between these two scenarios regardless of which missing data handling approach we used. That is, differences in missing data patterns were not a contributor of the different estimation results evidenced across missing data handling approaches.

Finally, the simulation studies thus far did not evaluate the effects of the different missing data handling approaches on type I error rates, namely, the probability of falsely rejecting the null hypothesis (typically a statement of null effects). To examine whether there was a type I error inflation issue in our study, we chose one condition (i.e., the low-autocorrelation and factor-dependent missingness condition since low autocorrelation has been found in many previous empirical studies (e.g., Li et al., 2022; You et al., 2020) and factor-dependent missingness is of particular interest in this study) and conducted a small simulation study with a model with several null effects by setting the true values of cross-regression parameters and coefficients of covariates to 0. Results can be found in Tables S24–S26 in the Supplementary Material. We found that the type I error rates generally fell into a reasonable range (e.g., below 0.08) for most parameters, except for certain covariate-related coefficients under PMI-MV and MI-FS, for which the type I error rate were elevated (around 0.14). Further inspection showed that the MI-MV consistently yielded higher coverage rates than PMI-MV and MI-FS for covariate-related parameters across the four simulation conditions (see, e.g., c1, c2, d1, d2 in Figure 8). Our speculation was that that these inflations in type I errors might stem from distinct sources of misspecification of the imputation models utilized under these approaches. That is, in the true missing data generation model, the missingness in the covariates depended on the covariate values, but not latent factors or the manifest dependent variables (see Equation (8)). As mentioned before, in the imputation model for the PMI-MV, missing values in both dependent variables and covariates were iteratively imputed *via* chained equations, while the imputed values of dependent variables were discarded. Thus, some of the variability contained in imputed dependent variables was lost. In the MI-FS, the latent factor scores used in the imputation model were estimates based on a model with no covariates. Whereas these factor scores were still helpful in improving the dynamic parameter estimates (e.g., cross-regression parameters, $b_{1}$ and $b_{2}$) without notable inflation in type I error rates, the inclusion of both dependent variables and factor scores for imputation purposes in the current MI-FS approach might have induced additional spurious associations shared by the dependent variables and covariates. Overall, this suggested that these approaches would benefit from having improved imputation models and variables for generating missing covariate values.

### Recommendations for the selection of missing data handling methods

To summarize, no one method could be declared as the best method universally, but we could select more appropriate methods depending on our needs. Based on our simulation results across 2 × 2 conditions, we can offer the following recommendations. First, the LD approach would not be recommended under any circumstances when handling longitudinal missing data. Second, if missingness was triggered by or at least associated with latent factors and the dynamics of latent factors (e.g., AR and CR parameters) were of more interest to researchers, then MI-FS and PMI-MV would be recommended given their better overall performances on these dynamic parameters. Third, the PMI-MV approach may be preferred under the high-autocorrelation condition, due to its comparable to better performance compared to MI-FS (e.g., comparable to smaller RMSEs, as well as higher coverage rates), and it is relatively easier to implement. Fourth, it is recommended to include missingness indicators as auxiliary variables in the imputation model, especially under NMAR missingness to inform the missingness mechanism. However, convergence needs to be guaranteed while implementing the MI algorithm. Finally, it is always recommended that users try all three MI-based methods to see if there are notable discrepancies. If different directions of effect were found under different approaches, it is suggested that users do not trust those specific results and implement further inspections to see if there are any mistakes made in the imputation and/or model fitting process.

## Empirical illustration

### Data descriptions

Data analyzed below were collected as part of the ADID study, in which 217 participants aged from 18 to 86 years old were asked to rate their momentary emotions five times a day over a month. Participants’ self-reported negative affect (NA) was measured using items from the Positive Affect and Negative Affect Schedule (PANAS; Watson et al., 1988) and other items posited in the circumplex model of affect (Larsen & Diener, 1992; Russell, 1980). For each item, participants were asked to rate on a four-point scale (1 = never; 4 = very often) the extent to which the affect has been experienced. Through item parceling (Kishton & Widaman, 1994), we created three item parcels^1^ as indicators of the latent variable, NA. Participants’ levels of PSS were measured *via* a five-item short-form of the Perceived Stress Scale (Cohen et al., 1983). For each item, participants were asked to rate on a five-point scale (1 = never; 5 = very often) how often they felt or thought in a certain way (e.g., how often they have felt nervous and stressed). We then computed the composite score on these items and used it as the indicator of PSS. Finally, participants’ personality states, including extraversion, emotional stability (reversely coded neuroticism), agreeableness, openness, and conscientiousness, were measured *via* selected questions from the revised NEO personality inventory (NEO-PI-R) where participants were asked to rate on a four-point scale (1 = never; 4 = very often) the extent to which they have felt about a word describing emotions since the last assessment.^2^

Because the proposed model hypothesized that the data were equally spaced, we followed the data pre-processing procedures adopted in previous studies (Chow & Zhang, 2013; You et al., 2020), and aggregated the data to two equally spaced data blocks per day, yielding a total of 26 to 74 measurement occasions per participant, with an average missing data proportion of 0.18. To remove the linear trends in NA and PSS, we first regressed the indicators of NA and PSS on measurement occasions, respectively. We then extracted the corresponding residuals, and added the person-specific means back to obtain the final scores for indicators of NA and PSS to be used in modeling fitting.

After all these data preprocessing, we fitted the model presented in the Motivating Example Section to the data, where missingness were handled by the four missing data handling approaches (i.e., LD, MI-MV, PMI-MV, MI-FS) to compare the results. The steps for implementing the MI-FS approach are provided below. MI-MV and PMI-MV approaches basically followed the same steps except for the differences illustrated in the previous section. Specifically, we first obtained factor scores by fitting a PFA model without including the four time-varying covariates in Equation (1). Then we multiply imputed both dependent variables and covariates using an imputation model consisting of (1) four dependent variables (three indicators of NA and one indicator of PSS) and four time-varying covariates (extraversion, emotional stability, agreeableness, and negative events); (2) two factor scores (smoothed estimates of latent factors, NA and PSS); (3) lags of variables in (1) and (2); (4) eight indicators for missingness in dependent variables and covariates; and (5) two auxiliary variables (openness and conscientiousness). Lastly, we fitted the PFA model defined in Equations (1)–(3) to each imputed data set and pooled estimation results.

### Empirical results

The empirical results are summarized in Table 2. We will first discuss similar results found across all four methods, followed by major differences on certain parameters across different methods.

First, with all methods, we found moderate inertia (see $a_{1}$ and $a_{2}$ in Table 2) in the dynamics of NA and PSS, indicating that levels of NA (PSS) were positively associated with previous levels of NA (PSS). Second, consistent with previous studies (Park et al., 2020), we found positive and moderate associations between concurrent NA and PSS, as indicated by the process noise covariance estimate (i.e., $\sigma_{\zeta_{12}}$). Third, in terms of relationships between personality states, NA, and PSS, previous studies have reported that extraversion and agreeableness were negatively associated with NA and PSS, while neuroticism was positively associated with NA and PSS (Ching et al., 2014; Ebstrup et al., 2011; Leger et al., 2016). With the exception of MI-FS on $d_{1}$, most missingness handling approaches suggested that participants tended to report lower levels of NA and PSS when they had higher concurrent levels of emotional stability (see $d_{1}$ and $d_{2}$) and agreeableness (see $e_{1}$ and $e_{2}$). Participants’ levels of extraversion were found to be negatively associated with PSS (see $c_{2}$) but not NA (see $c_{1}$). Fourth, from the inter-individual perspective, our study found individual differences in participants’ baseline levels of NA and PSS. Specifically, we found lower average levels of PSS among older people (see $\gamma_{22}$), which might be due to less exposure to daily stressors reported in old age (Stawski et al., 2008), and lower emotional and physical reactivity to interpersonal stressors (Neupert et al., 2007). Lower average levels of NA and PSS were also found associated with higher average levels of emotional stability (see $\gamma_{14}$ and $\gamma_{24}$), which was consistent with previous findings about positive associations between neuroticism and NA (Leger et al., 2016; Zhang & Zheng, 2019) as well as between neuroticism and PSS (Ebstrup et al., 2011). In particular, Duggan et al. (1995) found that individuals high in neuroticism responded more negatively to daily stressors and reported more daily stressful events and higher levels of daily stress (Duggan et al., 1995). Lastly, gender and other personality traits including extraversion and agreeableness were not found to be associated with individual differences in baseline levels of NA and PSS. This was in line with some previous studies showing that different from neuroticism, extraversion was not associated with between-person differences in the average levels of daily negative emotion (Zhang & Zheng, 2019).

In addition to the abovementioned consistent findings, the four missing data handling methods yielded different estimation results and/or levels of estimation uncertainty on several parameters. First, although AR parameters were found significant across all methods, their magnitudes differed—that is, lower levels of autocorrelation were found under LD and MI-MV. The smaller AR parameter estimates under LD were expected since removing cases with missing covariates altered the time dependency between observations, thus yielding less autocorrelation in the time series. The notable deviations in AR parameter estimates (especially $a_{1}$) under MI-MV might be due to the relatively worse imputation of indicators of NA which did not incorporate factor scores in the imputation model, thus highlighting the necessity of including factor scores in MI-MV.

Second, in terms of CR parameters, for the cross-lagged effect of PSS on NA (i.e., $b_{1}$), the estimates under LD and MI-MV were close to 0 and not significant, while under PMI-MV and MI-FS, the magnitudes of cross-lagged effect were a bit larger and the estimates were significant; for the cross-lagged effect of NA on PSS (i.e., $b_{2}$), all methods yielded significant results with different magnitudes of point estimates. Previous studies utilizing VAR models have found individuals who experienced higher levels of PSS tended to have higher levels of NA at the subsequent timepoint. In contrast, our results suggested negative associations between current levels of PSS and subsequent levels of NA and vice versa. This might be related to our data preprocessing where we aggregated data into four equally-spaced blocks per day. One speculation was that the length of time window in each block (i.e., 6 h) might lead to diminished influence of PSS on itself as well as on NA in the next time block. We note that cross-lagged relationships appeared highly sensitive to choices of the missing data handling techniques and would thus caution the reader to exercise caution in interpreting CR parameters.

Third, in terms of the effects of a set of time-varying covariates (e.g., personality states and negative events) on participants’ levels of NA and PSS, LD and PMI-MV yielded similar results. In contrast, MI-MV and MI-FS, produced slightly different results. Specifically, due to considerably larger standard error estimates under MI-MV, even if the point estimates were comparable to those under other approaches, some of them were not significant (e.g., $c_{2}$, $f_{1}$, and $f_{2}$). In addition, MI-FS yielded much smaller point estimates on $d_{1}$ than other methods. The reasons why the effect of emotional stability on levels of NA (i.e., $d_{1}$) was smaller under the MI-FS method might be that such effect was mostly captured by the higher AR parameter (i.e., $a_{1}$) which can be conceptually understood as higher inertia in NA dynamics and thus higher emotional stability (Kuppens et al., 2010).

Fourth, the MI-FS led to the smallest estimates of process noise (co)variance parameters, which was consistent with our findings in the simulation study that the MI-FS tended to underestimate process noise variances probably due to some losses in data variability at the item level when factor scores were included to the imputation model.

Fifth, another major difference between these methods lied in the estimates of covariances between measurement errors of three indicators of NA, which were significant and positive under PMI-MV. This might indicate that other methods failed to discover the existence of some common factors influencing these indicator variables. For instance, there might be systematic response patterns among indicator variables that were unrelated to the latent factor.

Finally, in terms of estimation efficiency, the MI-FS yielded more efficient estimates on AR and CR parameters than other methods, as indicated by smaller SE estimates, which were consistent with our findings in the simulation study. However, both MI-FS and MI-MV yielded larger SE estimates for the effects of time-varying covariates (see $c_{1}-f_{2}$) than LD and PMI-MV. Since the only difference between MI-MV and PMI-MV was whether MI was applied to dependent variables, the reason for such discrepancy in SE estimates might be that the MI approach generated some imputed values for dependent variables that rendered the corresponding time series unstable, which in turn affected the estimates of the relationships between dependent variables and time-varying covariates.

Overall, the four missing data handling approaches yielded consistent findings in terms of (1) moderate inertia in NA and PSS dynamics; (2) negative associations between concurrent NA/PSS and extraversion, emotional stability, and agreeableness; and (3) individual differences in the baseline levels of NA and PSS. Specifically, we found lower average levels of PSS among older people as well as lower average levels of NA and PSS among people with higher levels of emotional stability. Among the four approaches, MI-FS and PMI-MV yielded similar point estimates of AR and CR parameters, whereas MI-MV and LD tended to yield lower estimates of these parameters. Consistent with findings from the simulation study, the MI-FS yielded overall smaller SE estimates on dynamic model parameters compared with other approaches, indicating a reduction in estimation uncertainty by including factor scores in MI.

## Discussion

In this article, we proposed a novel multiple imputation strategy for longitudinal data, called MI-FS, to address possible types of nonignorable missingness across all items linked to the same common factor(s). The proposed method was designed with features tailored to the analysis of changes in latent factors over time, including factor scores, lag/lead variables, and missingness indicators into the imputation model to respectively account for missingness associated with latent factors, time dependencies between observations in longitudinal data, and missingness mechanisms.

The proposed method was evaluated and compared to LD and two MI methods without factor scores—MI-MV and PMI-MV, *via* both simulation and empirical studies. Simulation results showed that compared with MI without factor scores, MI-FS could yield overall less biased or similar estimates of AR and CR parameters. The advantage of MI-FS over MI-MV was more prominent when missingness was associated with latent factors. However, MI-FS tended to result in more biases in the variance parameters, which might be due to some losses in data variability at the item level when factor scores were included to generate imputed values. In addition, PMI-MV could yield comparable performance to MI-FS in recovering AR parameters under the high-autocorrelation condition, thus highlighting the capability of the FIML approach in handling missing data when combined with MI of missing covariates. In fact, the PMI-MV approach may offer advantages in terms of ease of implementation compared to MI-FS, particularly given its comparable performance to MI-FS in high-autocorrelation scenarios. Finally, compared to the LD method, which showed notable decrements in performance under high autocorrelations, the MI-based methods were relatively robust to variations in autocorrelations in the data set.

Our empirical illustration investigated the reciprocal linkages between NA and PSS over time as well as individual differences in their baseline levels. Despite the slight differences in the magnitudes of point estimates, the four missing data handling approaches led to overall similar results in terms of how NA and PSS were associated with each other and certain personality states over time, as well as the effects of personality traits on baseline levels of NA/PSS. Compared with other approaches, MI-FS yielded higher efficiency in AR and CR parameters, as indicated by their smaller SE estimates.

There were several unresolved issues in our studies. First of all, since our empirical study consisted of items characterized by theoretically informed factor structures, we adopted CFA models where indicators were item parcels used in previous studies (You et al., 2020). However, when the underlying factor structures are unknown or of interest, EFA needs to be conducted first to determine the number of latent constructs and the factor structure of a set of items/variables (Gilbert & Meijer, 2005). Second, in addition to the latent variables considered in the model (i.e., NA and PSS), the missingness in indicators may also be triggered by other common factors, which were not taken into account in this study. Third, as mentioned before, the time intervals between measurement points varied within and/or between individuals over time, and thus the raw data were aggregated to be equally spaced so that a discrete-time model could be fitted. However, modeling continuously changed processes simply as changing in discrete-time can be problematic. For instance, classical concerns include sign flipping and counter-intuitive effect directions in VAR models (Driver, 2022). Future research may consider applying continuous-time models such as continuous-time structural equation models (Voelkle et al., 2012) to accommodate unequally spaced EMA assessments.

Some possible future directions can be considered. First, our simulation study utilized relatively simple missing data generation models where the missingness in manifest variables was associated with either themselves or their corresponding latent variables. Under this simulation design, PMI-MV could yield comparable or even better results than MI-FS for most parameters. Future simulation studies may increase the complexity of the missing data generation model (e.g., missingness associated with both unobserved manifest variables and latent variables) and test the performance of PMI-MV under this more challenging situation. Also, future work may consider integrating features of MI-FS into joint modeling of the change processes of interest and their corresponding missingness mechanisms, such as through selection modeling or shared parameter approach (Creemers et al., 2010). Second, the model specified for the simulation study did not allow for any person-specific parameters. Since our focus was on the comparison between different missing data handling approaches, the model setup was sufficient for illustration purposes. However, in real data analysis, some modifications can be made to capture meaningful aspects of individual differences. For instance, in our empirical illustration, we included random effects for intercept parameters given the notable individual differences in baseline levels. Future work may consider also including random effects for AR parameters and investigate how predictors affect AR parameters—for instance, whether and how emotional stability is associated with the AR parameters. In addition to the multilevel modeling framework adopted in the present study, some alternative approaches also allow for higher-dimensional random effects, such as mixed effects models (Henderson, 1982) and fitting relevant models in the Bayesian framework to aid computational efficiency (Li et al., 2022). Note that missing data handling in the presence of these complex models may involve complex imputation models such as models with random slopes or nonlinear terms, in which scenario the incompatibility issue may arise in the MI procedure and Bartlett’s model-compatible specification may be utilized to address this issue (Bartlett & Morris, 2015). Finally, though illustrated in the context of the PFA models, the propose method can potentially be used with any dynamic models capturing changes of latent variables such as nonlinear dynamic factor models (Chow & Zhang, 2013; Tang et al., 2017), various types of latent variable models (Muthén & Curran, 1997; Roy & Lin, 2000), and continuous-time structural equation models (Voelkle et al., 2012). Future work may consider investigating the performance of MI-FS in the context of these different types of models.

Overall, we proposed an MI strategy suited for longitudinal analysis of psychological constructs. The idea of including factor scores in MI was guided by the general rule of choosing appropriate and relevant variables as predictors in the imputation model by taking missing data mechanisms into consideration. Through comparisons with other existing methods under various conditions in the simulation study, we provided our suggestions on the selection of missing data handling methods in different scenarios. Our proposed method is highly recommended for handling missing data in multiple-item scales as well as data analysis involving factor-analytic models.

## Supplementary Material

Supp 1

## Figures and Tables

**Table 1.** Summary of missing data handling methods considered in the present study.

Method	Missingness in DVs	Missingness in covariates	Use factor scores
LD	FIML	Removed	No
PMI-MV	FIML	MI	No
MI-MV	MI	MI	No
MI-FS	MI	MI	Yes

*Note*: DV: dependent variables; MI: multiple imputation; FIML: full-information maximum likelihood; LD: listwise deletion; PMI-MV: partial MI with manifest variables; MI-MV: MI with manifest variables; MI-FS: MI with factor scores.

**Table 2.** Comparison of empirical results.

	LD		MI-MV		PMI-MV		MI-FS	
Parameter	Est	SE	Est	SE	Est	SE	Est	SE
Level 1 dynamic model parameters (see Equation (1))								
AR and CR parameters								
$a_{1}$, ${\text{NA}}_{i,t-1}\to {\text{NA}}_{i,t}$	0.308*	0.028	0.198*	0.079	0.399*	0.028	0.448*	0.031
$a_{2}$, ${\text{PSS}}_{i,t-1}\to {\text{PSS}}_{i,t}$	0.404*	0.032	0.419*	0.073	0.444*	0.032	0.461*	0.027
$b_{1}$, ${\text{PSS}}_{i,t-1}\to {\text{NA}}_{i,t}$	−0.026	0.023	0.003	0.077	−0.080*	0.023	−0.049*	0.018
$b_{2}$, ${\text{NA}}_{i,t-1}\to {\text{PSS}}_{i,t}$	−0.106*	0.028	−0.171	0.087	−0.140*	0.029	−0.083*	0.025
Covariate-related coefficients								
$c_{1}$, Extrav→NA	−0.006	0.008	0.040	0.062	−0.010	0.008	−0.025	0.014
$c_{2}$, Extrav→PSS	−0.032*	0.009	−0.027	0.042	−0.030*	0.009	−0.043*	0.015
$d_{1}$, EmoStab→NA	−0.123*	0.009	−0.167*	0.050	−0.115*	0.009	−0.035	0.024
$d_{2}$, EmoStab→PSS	−0.139*	0.010	−0.108*	0.041	−0.134*	0.010	−0.057*	0.017
$e_{1}$, Agree→NA	−0.183*	0.009	−0.214*	0.037	−0.181*	0.009	−0.117*	0.028
$e_{2}$, Agree→PSS	−0.177*	0.010	−0.186*	0.029	−0.177*	0.010	−0.113*	0.025
$f_{1}$, NegEvents→NA	0.043*	0.008	0.028	0.021	0.042*	0.008	0.051*	0.012
$f_{2}$, NegEvents→PSS	0.045*	0.009	0.048	0.030	0.045*	0.009	0.053*	0.016
Process noise (co)variances								
$\sigma_{\zeta_{1}}^{2}$	0.478*	0.030	0.688*	0.182	0.415*	0.021	0.356*	0.019
$\sigma_{\zeta_{12}}$	0.365*	0.009	0.481*	0.038	0.364*	0.009	0.302*	0.010
$\sigma_{\zeta_{2}}^{2}$	0.516*	0.028	0.578*	0.067	0.499*	0.025	0.460*	0.025
Level 2 dynamic model parameters (see Equation (2))								
Fixed effects								
$\gamma_{10}$, Intercept, NA	1.424*	0.084	1.563*	0.127	1.457*	0.085	1.384*	0.085
$\gamma_{20}$, Intercept, PSS	1.917*	0.112	1.927*	0.113	1.916*	0.110	1.850*	0.108
$\gamma_{11}$, Gender→NA	−0.043	0.048	−0.067	0.053	−0.058	0.048	−0.045	0.046
$\gamma_{21}$, Gender→PSS	−0.017	0.063	−0.018	0.057	−0.015	0.062	0.000	0.059
$\gamma_{12}$, Age→NA	−0.022	0.023	−0.047	0.027	−0.021	0.023	−0.010	0.022
$\gamma_{22}$, Age→PSS	−0.098*	0.030	−0.094*	0.032	−0.092*	0.030	−0.086*	0.030
$\gamma_{13}$, ExtravMean→NA	−0.033	0.024	−0.025	0.023	−0.024	0.024	−0.021	0.023
$\gamma_{23}$, ExtravMean→PSS	−0.043	0.032	−0.043	0.028	−0.042	0.031	−0.040	0.030
$\gamma_{14}$, EmoStabMean→NA	−0.109*	0.028	−0.091*	0.027	−0.100*	0.028	−0.107*	0.027
$\gamma_{24}$, EmoStabMean→PSS	−0.138*	0.037	−0.130*	0.033	−0.129*	0.036	−0.134*	0.035
$\gamma_{15}$, AgreeMean→NA	−0.035	0.029	−0.050	0.028	−0.043	0.029	−0.042	0.028
$\gamma_{25}$, AgreeMean→PSS	−0.046	0.039	−0.061	0.035	−0.060	0.037	−0.062	0.036
$\gamma_{16}$, NegEventsMean→NA	0.083*	0.024	0.074*	0.024	0.075*	0.024	0.077*	0.023
$\gamma_{26}$, NegEventsMean→PSS	0.108*	0.032	0.102*	0.029	0.104*	0.031	0.105*	0.030
Random effects								
$\sigma_{e_{1}}^{2}$	0.076*	0.010	0.069*	0.013	0.080*	0.010	0.071*	0.009
$\sigma_{e_{12}}$	0.068*	0.011	0.056*	0.010	0.071*	0.011	0.064*	0.010
$\sigma_{e_{2}}^{2}$	0.142*	0.017	0.111*	0.013	0.142*	0.017	0.133*	0.015
Measurement model parameters (see Equation (3))								
$\lambda_{1}$	1.028*	0.015	1.057*	0.070	1.031*	0.015	1.032*	0.022
$\lambda_{2}$	0.999*	0.015	0.999*	0.037	1.003*	0.015	1.026*	0.022
$\sigma_{\epsilon_{1}}^{2}$	0.360*	0.028	0.476*	0.097	0.420*	0.019	0.384*	0.024
$\sigma_{\epsilon_{2}}^{2}$	0.320*	0.028	0.365*	0.111	0.379*	0.020	0.353*	0.019
$\sigma_{\epsilon_{3}}^{2}$	0.361*	0.027	0.427*	0.107	0.417*	0.019	0.365*	0.016
$\sigma_{\epsilon_{4}}^{2}$	0.290*	0.025	0.389*	0.056	0.307*	0.022	0.222*	0.020
$\sigma_{\epsilon_{12}}$	0.015	0.027	0.049	0.104	0.070*	0.018	0.041	0.023
$\sigma_{\epsilon_{13}}$	0.025	0.026	0.076	0.103	0.078*	0.018	0.034*	0.017
$\sigma_{\epsilon_{23}}$	0.017	0.027	0.045	0.108	0.078*	0.018	0.037	0.022

*Notes*: Est: point estimates; SE: standard error estimates;

* *p* < 0.05. Results were obtained based on 217 participants and 26 to 74 measurement occasions per participant.

## Footnotes

1 Items included in the three parcels were: (1) parcel 1: angry, sad, distressed, jittery, guilty and afraid; (2) parcel 2: upset, hostile, irritable, tense and ashamed, and (3) parcel 3: depressed, agitated, nervous, anxious and scared.

2 For instance, words used to measure extraversion included *Passive* vs. *Active*; *Unenergetic* vs. *Energetic*; and *Dominant* vs. *Submissive*.
