# Supplementary material for: Multiple Imputation with Factor Scores: A Practical Approach for Handling Simultaneous Missingness Across Items in Longitudinal Designs
Source: Multivariate Behav Res. Author manuscript; Available in PMC 2025 Apr 2. (PMC11724938; doi:10.1080/00273171.2024.2371816)
Supplement: Supp 1 [file NIHMS2008219-supplement-Supp_1.pdf]

Supplementary Material: Full Simulation Results

Part I. Main Simulation Results

Simulation results with different missing data handling approaches under different conditions are summarized in Tables S1 - S16. In the following tables, “LD” = Listwise deletion; “PMI-MV” = Partial multiple imputation (MI) with manifest variables; “MI-MV” = MI with manifest variables; “MI-FS” = MI with factor scores; “True” represents the true value; “RBias” represents the relative bias; “CR” represents the coverage rate. Detailed descriptions about the four missing data handling approaches and definitions of the summary statistics can be found in the main article.

Part II. MI without Manifest Variables

In this section, we considered an alternative approach where manifest variables were not included in MI — that is, when imputing a specific manifest variable, other manifest variables would not be included in the imputation model, and tested the performance of this approach under one simulation condition (i.e., high-autocorrelation and factor-dependent missingness) by running a small simulation study (100 replications). By comparing results in Tables S15 – S17, we found that although estimates of the dynamic model were satisfactory, estimates of parameters in the measurement model were more biased with this approach compared with either MI-FS or MI-MV under the same simulation condition. Results indicated that manifest variables (or observed indicators) also played a critical role in the MI process.

Part III. Simulation with Scattered vs. Simultaneous Missing Data Patterns

To make sure that the simultaneous vs. scattered missing data patterns did not confound the comparisons across missing data handling approaches, we conducted a small simulation study by considering two alternative missingness generation scenarios — randomly generated scattered vs. simultaneous missingness (see details in the main text). We found comparable estimation results between these two scenarios regardless of which missing data handling approach we used (see Tables S18 – S23). That is, differences in missing data patterns were not a contributor of the different estimation results evidenced across missing data handling approaches in Part I.

Part IV. Simulation with Null Effects

To examine whether there was a type I error inflation issue in our study, we conducted a small simulation study with a model with several null effects by setting the true values of cross-regression parameters and coefficients of covariates to 0. Results in Table S24 – S26 showed that type I error rates generally fell into a reasonable range (e.g., below 0.08) for most parameters (see more detailed discussion in the main text).

Table S1

Simulation results based on LD and data with (1) a low level of autocorrelation and (2) item-dependent missingness

	True	Bias	RBias	SE	MCSE	dSEfull	RMSE	Power	CR(%)
a_1	0.5	-0.15	-0.30	0.03	0.02	0.02	0.15	1.00	0
a_2	0.5	-0.16	-0.32	0.03	0.03	0.01	0.16	1.00	0
b_1	-0.2	0.03	-0.13	0.02	0.02	0.01	0.03	1.00	68
b_2	-0.3	0.05	-0.17	0.04	0.04	0.02	0.07	1.00	73
c_1	0.3	0.09	0.28	0.08	0.09	0.03	0.12	0.99	82
c_2	-0.3	-0.12	0.40	0.13	0.14	0.04	0.18	0.86	85
d_1	0.5	0.04	0.08	0.04	0.04	0.02	0.06	1.00	84
d_2	-0.4	-0.08	0.20	0.06	0.06	0.02	0.10	1.00	75
$\sigma^2_{\zeta_1}$	2.0	0.32	0.16	0.12	0.12	0.05	0.35	1.00	21
$\sigma_{\zeta_{12}}$	0.5	-0.77	-1.53	0.11	0.13	0.04	0.78	0.63	0
$\sigma^2_{\zeta_2}$	6.0	0.09	0.01	0.27	0.29	0.10	0.30	1.00	93
μ_1	3.0	0.51	0.17	0.11	0.12	0.00	0.52	1.00	1
μ_2	3.0	1.07	0.36	0.21	0.22	0.00	1.09	1.00	0
μ_3	3.0	0.51	0.17	0.11	0.12	0.00	0.52	1.00	1
μ_4	3.0	-0.72	-0.24	0.16	0.17	0.00	0.74	1.00	1
μ_5	3.0	-1.52	-0.51	0.31	0.33	-0.01	1.56	1.00	0
μ_6	3.0	-0.72	-0.24	0.16	0.17	0.00	0.74	1.00	1
λ_1	2.0	-0.05	-0.02	0.04	0.04	0.02	0.06	1.00	76
λ_2	1.0	0.00	0.00	0.02	0.02	0.01	0.02	1.00	93
λ_3	2.0	-0.04	-0.02	0.03	0.03	0.02	0.05	1.00	71
λ_4	1.0	0.00	0.00	0.02	0.02	0.01	0.02	1.00	95
$\sigma^2_{\epsilon_1}$	1.0	-0.03	-0.03	0.06	0.06	0.03	0.06	1.00	91
$\sigma^2_{\epsilon_2}$	1.0	0.02	0.02	0.14	0.14	0.06	0.14	1.00	96
$\sigma^2_{\epsilon_3}$	1.0	-0.03	-0.03	0.06	0.06	0.03	0.06	1.00	90
$\sigma^2_{\epsilon_4}$	1.0	-0.02	-0.02	0.06	0.06	0.03	0.06	1.00	94
$\sigma^2_{\epsilon_5}$	1.0	0.01	0.01	0.15	0.15	0.06	0.16	1.00	94
$\sigma^2_{\epsilon_6}$	1.0	-0.02	-0.02	0.06	0.06	0.03	0.06	1.00	91

Table S2

Simulation results based on PMI-MV and data with (1) a low level of autocorrelation and (2) item-dependent missingness

	True	Bias	RBias	SE	MCSE	dSEfull	RMSE	Power	CR(%)
a_1	0.5	-0.03	-0.06	0.02	0.02	0.01	0.03	1.00	61
a_2	0.5	-0.04	-0.08	0.02	0.02	0.00	0.04	1.00	44
b_1	-0.2	0.01	-0.03	0.01	0.01	0.00	0.01	1.00	93
b_2	-0.3	0.03	-0.11	0.03	0.03	0.01	0.04	1.00	73
c_1	0.3	0.01	0.04	0.06	0.06	0.01	0.06	1.00	96
c_2	-0.3	-0.03	0.10	0.10	0.10	0.01	0.11	0.89	93
d_1	0.5	0.04	0.09	0.03	0.03	0.01	0.05	1.00	61
d_2	-0.4	-0.09	0.23	0.04	0.04	0.00	0.10	1.00	41
$\sigma^2_{\zeta_1}$	2.0	-0.23	-0.11	0.07	0.07	0.00	0.24	1.00	13
$\sigma_{\zeta_{12}}$	0.5	-0.20	-0.40	0.07	0.08	0.00	0.21	0.98	23
$\sigma^2_{\zeta_2}$	6.0	-1.02	-0.17	0.17	0.17	0.00	1.03	1.00	0
μ_1	3.0	0.22	0.07	0.11	0.12	0.00	0.25	1.00	48
μ_2	3.0	0.49	0.16	0.21	0.22	0.00	0.54	1.00	37
μ_3	3.0	0.22	0.07	0.11	0.11	0.00	0.25	1.00	50
μ_4	3.0	-0.42	-0.14	0.16	0.17	0.00	0.45	1.00	28
μ_5	3.0	-0.92	-0.31	0.31	0.33	-0.01	0.98	1.00	18
μ_6	3.0	-0.42	-0.14	0.16	0.17	0.00	0.45	1.00	26
λ_1	2.0	-0.06	-0.03	0.03	0.03	0.01	0.07	1.00	52
λ_2	1.0	0.00	0.00	0.02	0.02	0.01	0.02	1.00	95
λ_3	2.0	-0.04	-0.02	0.02	0.02	0.01	0.04	1.00	52
λ_4	1.0	0.00	0.00	0.01	0.01	0.00	0.01	1.00	93
$\sigma^2_{\epsilon_1}$	1.0	-0.04	-0.04	0.04	0.04	0.01	0.06	1.00	83
$\sigma^2_{\epsilon_2}$	1.0	0.03	0.03	0.10	0.10	0.02	0.10	1.00	96
$\sigma^2_{\epsilon_3}$	1.0	-0.04	-0.04	0.04	0.04	0.01	0.06	1.00	82
$\sigma^2_{\epsilon_4}$	1.0	-0.03	-0.03	0.04	0.04	0.01	0.05	1.00	88
$\sigma^2_{\epsilon_5}$	1.0	0.02	0.02	0.11	0.12	0.02	0.12	1.00	93
$\sigma^2_{\epsilon_6}$	1.0	-0.03	-0.03	0.04	0.04	0.01	0.05	1.00	90

Table S3
Simulation results based on MI-MV and data with (1) a low level of autocorrelation and (2) item-dependent missingness

	True	Bias	RBias	SE	MCSE	dSEfull	RMSE	Power	CR(%)
a_1	0.5	-0.04	-0.08	0.02	0.01	0.01	0.04	1.00	32
a_2	0.5	-0.03	-0.07	0.02	0.02	0.00	0.04	1.00	52
b_1	-0.2	0.01	-0.03	0.01	0.01	0.00	0.01	1.00	92
b_2	-0.3	0.02	-0.05	0.03	0.03	0.01	0.03	1.00	89
c_1	0.3	0.00	0.00	0.07	0.06	0.02	0.06	0.99	95
c_2	-0.3	0.03	-0.12	0.11	0.11	0.02	0.11	0.70	92
d_1	0.5	0.01	0.03	0.03	0.03	0.01	0.03	1.00	93
d_2	-0.4	0.01	-0.02	0.04	0.04	0.00	0.04	1.00	94
$\sigma^2_{\zeta_1}$	2.0	0.02	0.01	0.09	0.09	0.02	0.09	1.00	95
$\sigma_{\zeta_{12}}$	0.5	-0.22	-0.45	0.08	0.08	0.01	0.24	0.93	20
$\sigma^2_{\zeta_2}$	6.0	-0.31	-0.05	0.21	0.22	0.04	0.38	1.00	65
μ_1	3.0	0.16	0.05	0.12	0.12	0.01	0.20	1.00	70
μ_2	3.0	0.37	0.12	0.22	0.22	0.01	0.43	1.00	60
μ_3	3.0	0.16	0.05	0.12	0.12	0.01	0.20	1.00	73
μ_4	3.0	-0.20	-0.07	0.17	0.18	0.01	0.27	1.00	77
μ_5	3.0	-0.53	-0.18	0.33	0.36	0.01	0.64	1.00	62
μ_6	3.0	-0.20	-0.07	0.17	0.18	0.01	0.27	1.00	77
λ_1	2.0	-0.08	-0.04	0.03	0.04	0.01	0.09	1.00	28
λ_2	1.0	0.00	0.00	0.02	0.02	0.01	0.02	1.00	93
λ_3	2.0	-0.07	-0.04	0.03	0.03	0.02	0.08	1.00	23
λ_4	1.0	0.00	0.00	0.01	0.02	0.00	0.02	1.00	90
$\sigma^2_{\epsilon_1}$	1.0	-0.04	-0.04	0.04	0.05	0.01	0.06	1.00	81
$\sigma^2_{\epsilon_2}$	1.0	0.10	0.10	0.11	0.11	0.03	0.15	1.00	83
$\sigma^2_{\epsilon_3}$	1.0	-0.05	-0.05	0.04	0.05	0.01	0.06	1.00	77
$\sigma^2_{\epsilon_4}$	1.0	-0.01	-0.01	0.05	0.05	0.02	0.05	1.00	88
$\sigma^2_{\epsilon_5}$	1.0	0.15	0.15	0.13	0.13	0.04	0.20	1.00	77
$\sigma^2_{\epsilon_6}$	1.0	-0.03	-0.03	0.05	0.05	0.02	0.06	1.00	88

Table S4

Simulation results based on MI-FS and data with (1) a low level of autocorrelation and (2) item-dependent missingness

	True	Bias	RBias	SE	MCSE	dSEfull	RMSE	Power	CR(%)
a_1	0.5	0.01	0.02	0.01	0.02	0.00	0.02	1.00	83
a_2	0.5	-0.01	-0.03	0.02	0.02	0.00	0.02	1.00	81
b_1	-0.2	0.00	-0.01	0.01	0.01	0.00	0.01	1.00	90
b_2	-0.3	0.03	-0.09	0.02	0.03	0.00	0.04	1.00	70
c_1	0.3	-0.04	-0.13	0.06	0.05	0.01	0.07	1.00	89
c_2	-0.3	0.01	-0.04	0.09	0.09	0.00	0.10	0.86	94
d_1	0.5	-0.04	-0.09	0.02	0.03	0.00	0.05	1.00	52
d_2	-0.4	-0.02	0.04	0.04	0.04	0.00	0.04	1.00	91
$\sigma^2_{\zeta_1}$	2.0	-0.40	-0.20	0.06	0.08	-0.01	0.41	1.00	0
$\sigma_{\zeta_{12}}$	0.5	-0.23	-0.46	0.06	0.07	-0.01	0.24	0.98	5
$\sigma^2_{\zeta_2}$	6.0	-1.49	-0.25	0.14	0.16	-0.03	1.50	1.00	0
μ_1	3.0	0.19	0.06	0.11	0.12	0.00	0.23	1.00	58
μ_2	3.0	0.52	0.17	0.21	0.22	0.00	0.56	1.00	29
μ_3	3.0	0.19	0.06	0.11	0.12	0.00	0.23	1.00	58
μ_4	3.0	-0.44	-0.15	0.16	0.17	0.00	0.47	1.00	23
μ_5	3.0	-0.97	-0.32	0.31	0.33	-0.01	1.03	1.00	16
μ_6	3.0	-0.46	-0.15	0.16	0.17	0.00	0.49	1.00	19
λ_1	2.0	-0.10	-0.05	0.02	0.03	0.00	0.11	1.00	5
λ_2	1.0	-0.01	-0.01	0.02	0.03	0.01	0.03	1.00	78
λ_3	2.0	-0.08	-0.04	0.02	0.03	0.01	0.09	1.00	8
λ_4	1.0	-0.04	-0.04	0.01	0.01	0.00	0.04	1.00	15
$\sigma^2_{\epsilon_1}$	1.0	-0.23	-0.23	0.03	0.04	0.00	0.24	1.00	0
$\sigma^2_{\epsilon_2}$	1.0	-0.22	-0.22	0.07	0.17	-0.01	0.28	1.00	12
$\sigma^2_{\epsilon_3}$	1.0	-0.17	-0.17	0.03	0.17	0.00	0.24	1.00	0
$\sigma^2_{\epsilon_4}$	1.0	-0.26	-0.26	0.04	0.05	0.01	0.26	1.00	1
$\sigma^2_{\epsilon_5}$	1.0	0.53	0.53	0.14	0.15	0.05	0.55	1.00	4
$\sigma^2_{\epsilon_6}$	1.0	0.40	0.40	0.09	0.12	0.06	0.42	1.00	0

Table S5

Simulation results based on LD and data with (1) a high level of autocorrelation and (2) item-dependent missingness

	True	Bias	RBias	SE	MCSE	dSEfull	RMSE	Power	CR(%)
a_1	0.7	-0.10	-0.15	0.02	0.02	0.01	0.11	1.00	0
a_2	0.7	-0.13	-0.18	0.02	0.03	0.01	0.13	1.00	0
b_1	-0.2	-0.05	0.23	0.02	0.02	0.01	0.05	1.00	16
b_2	-0.3	-0.06	0.21	0.03	0.03	0.01	0.07	1.00	50
c_1	0.3	0.14	0.47	0.09	0.10	0.04	0.17	0.99	67
c_2	-0.3	-0.19	0.63	0.14	0.15	0.05	0.24	0.93	73
d_1	0.5	0.01	0.02	0.04	0.04	0.02	0.04	1.00	96
d_2	-0.4	-0.03	0.08	0.07	0.07	0.03	0.08	1.00	92
$\sigma^2_{\zeta_1}$	2.0	0.97	0.49	0.14	0.15	0.08	0.98	1.00	0
$\sigma_{\zeta_{12}}$	0.5	-1.18	-2.36	0.14	0.17	0.07	1.19	0.99	0
$\sigma^2_{\zeta_2}$	6.0	1.46	0.24	0.32	0.37	0.15	1.51	1.00	0
μ_1	3.0	1.43	0.48	0.40	0.44	0.00	1.50	1.00	9
μ_2	3.0	2.86	0.95	0.79	0.88	-0.01	2.99	1.00	9
μ_3	3.0	1.43	0.48	0.40	0.44	0.00	1.49	1.00	9
μ_4	3.0	-1.95	-0.65	0.50	0.54	-0.03	2.02	0.55	6
μ_5	3.0	-3.91	-1.30	0.99	1.09	-0.07	4.06	0.19	5
μ_6	3.0	-1.95	-0.65	0.50	0.54	-0.03	2.03	0.56	6
λ_1	2.0	-0.01	0.00	0.02	0.02	0.01	0.02	1.00	91
λ_2	1.0	0.00	0.00	0.01	0.01	0.00	0.01	1.00	95
λ_3	2.0	-0.01	0.00	0.02	0.02	0.01	0.02	1.00	91
λ_4	1.0	0.00	0.00	0.01	0.01	0.01	0.01	1.00	94
$\sigma^2_{\epsilon_1}$	1.0	-0.02	-0.02	0.05	0.05	0.02	0.06	1.00	92
$\sigma^2_{\epsilon_2}$	1.0	0.02	0.02	0.13	0.13	0.06	0.13	1.00	96
$\sigma^2_{\epsilon_3}$	1.0	-0.03	-0.03	0.05	0.05	0.02	0.06	1.00	92
$\sigma^2_{\epsilon_4}$	1.0	-0.01	-0.01	0.06	0.06	0.03	0.06	1.00	94
$\sigma^2_{\epsilon_5}$	1.0	0.00	0.00	0.15	0.15	0.07	0.15	1.00	95
$\sigma^2_{\epsilon_6}$	1.0	-0.02	-0.02	0.06	0.06	0.03	0.06	1.00	93

Table S6

Simulation results based on PMI-MV and data with (1) a high level of autocorrelation and (2) item-dependent missingness

	True	Bias	RBias	SE	MCSE	dSEfull	RMSE	Power	CR(%)
a_1	0.7	-0.01	-0.01	0.01	0.01	0.00	0.01	1.00	94
a_2	0.7	-0.03	-0.04	0.02	0.02	0.01	0.03	1.00	62
b_1	-0.2	-0.01	0.03	0.01	0.01	0.00	0.01	1.00	91
b_2	-0.3	0.00	0.00	0.02	0.02	0.00	0.02	1.00	93
c_1	0.3	-0.02	-0.06	0.06	0.06	0.01	0.07	0.99	95
c_2	-0.3	0.02	-0.06	0.10	0.10	0.01	0.10	0.80	94
d_1	0.5	0.01	0.01	0.03	0.03	0.01	0.03	1.00	94
d_2	-0.4	-0.03	0.09	0.04	0.04	0.00	0.05	1.00	87
$\sigma^2_{\zeta_1}$	2.0	-0.16	-0.08	0.07	0.07	0.01	0.17	1.00	33
$\sigma_{\zeta_{12}}$	0.5	-0.12	-0.23	0.07	0.07	0.00	0.14	1.00	64
$\sigma^2_{\zeta_2}$	6.0	-0.74	-0.12	0.17	0.18	0.00	0.76	1.00	1
μ_1	3.0	0.25	0.08	0.43	0.44	0.03	0.50	1.00	87
μ_2	3.0	0.46	0.15	0.85	0.87	0.05	0.98	0.97	89
μ_3	3.0	0.25	0.08	0.43	0.44	0.03	0.50	1.00	88
μ_4	3.0	-0.66	-0.22	0.54	0.54	0.01	0.85	0.99	72
μ_5	3.0	-1.28	-0.43	1.08	1.07	0.02	1.67	0.37	72
μ_6	3.0	-0.66	-0.22	0.54	0.54	0.01	0.85	0.99	71
λ_1	2.0	-0.01	0.00	0.02	0.02	0.01	0.02	1.00	91
λ_2	1.0	0.00	0.00	0.01	0.01	0.00	0.01	1.00	94
λ_3	2.0	0.00	0.00	0.01	0.01	0.00	0.01	1.00	93
λ_4	1.0	0.00	0.00	0.01	0.01	0.01	0.01	1.00	95
$\sigma^2_{\epsilon_1}$	1.0	-0.03	-0.03	0.04	0.04	0.01	0.05	1.00	88
$\sigma^2_{\epsilon_2}$	1.0	0.02	0.02	0.09	0.09	0.02	0.09	1.00	94
$\sigma^2_{\epsilon_3}$	1.0	-0.03	-0.03	0.04	0.04	0.01	0.05	1.00	88
$\sigma^2_{\epsilon_4}$	1.0	-0.02	-0.02	0.04	0.04	0.01	0.05	1.00	90
$\sigma^2_{\epsilon_5}$	1.0	0.02	0.02	0.10	0.11	0.02	0.11	1.00	94
$\sigma^2_{\epsilon_6}$	1.0	-0.02	-0.02	0.04	0.04	0.01	0.05	1.00	91

Table S7

Simulation results based on MI-MV and data with (1) a high level of autocorrelation and (2) item-dependent missingness

	True	Bias	RBias	SE	MCSE	dSEfull	RMSE	Power	CR(%)
a_1	0.7	-0.03	-0.04	0.01	0.01	0.00	0.03	1.00	40
a_2	0.7	-0.04	-0.06	0.02	0.02	0.01	0.04	1.00	31
b_1	-0.2	-0.01	0.06	0.01	0.01	0.00	0.02	1.00	79
b_2	-0.3	-0.01	0.05	0.02	0.02	0.00	0.03	1.00	88
c_1	0.3	-0.01	-0.03	0.07	0.07	0.02	0.07	0.98	96
c_2	-0.3	0.03	-0.11	0.11	0.10	0.02	0.10	0.72	94
d_1	0.5	-0.01	-0.03	0.03	0.03	0.01	0.03	1.00	94
d_2	-0.4	0.03	-0.07	0.05	0.04	0.01	0.05	1.00	92
$\sigma^2_{\zeta_1}$	2.0	0.28	0.14	0.10	0.12	0.04	0.31	1.00	22
$\sigma_{\zeta_{12}}$	0.5	-0.39	-0.77	0.09	0.11	0.02	0.40	0.30	1
$\sigma^2_{\zeta_2}$	6.0	-0.04	-0.01	0.23	0.27	0.06	0.27	1.00	89
μ_1	3.0	0.44	0.15	0.44	0.40	0.04	0.60	0.99	77
μ_2	3.0	1.00	0.33	0.87	0.78	0.07	1.27	0.98	73
μ_3	3.0	0.44	0.15	0.44	0.40	0.04	0.59	0.99	78
μ_4	3.0	-0.71	-0.24	0.57	0.52	0.04	0.88	0.96	71
μ_5	3.0	-1.60	-0.53	1.11	1.01	0.05	1.89	0.27	64
μ_6	3.0	-0.70	-0.23	0.57	0.51	0.04	0.87	0.96	71
λ_1	2.0	-0.04	-0.02	0.02	0.03	0.01	0.05	1.00	46
λ_2	1.0	0.00	0.00	0.01	0.01	0.00	0.01	1.00	91
λ_3	2.0	-0.04	-0.02	0.02	0.03	0.01	0.05	1.00	46
λ_4	1.0	0.00	0.00	0.01	0.01	0.01	0.01	1.00	90
$\sigma^2_{\epsilon_1}$	1.0	0.01	0.01	0.05	0.05	0.02	0.05	1.00	91
$\sigma^2_{\epsilon_2}$	1.0	0.16	0.16	0.12	0.11	0.05	0.19	1.00	76
$\sigma^2_{\epsilon_3}$	1.0	-0.01	-0.01	0.05	0.05	0.02	0.05	1.00	91
$\sigma^2_{\epsilon_4}$	1.0	0.04	0.04	0.06	0.07	0.03	0.08	1.00	86
$\sigma^2_{\epsilon_5}$	1.0	0.23	0.23	0.15	0.15	0.07	0.28	1.00	67
$\sigma^2_{\epsilon_6}$	1.0	0.02	0.02	0.06	0.07	0.03	0.07	1.00	92

Table S8

Simulation results based on MI-FS and data with (1) a high level of autocorrelation and (2) item-dependent missingness

	True	Bias	RBias	SE	MCSE	dSEfull	RMSE	Power	CR(%)
a_1	0.7	0.01	0.01	0.01	0.01	0.00	0.01	1.00	89
a_2	0.7	-0.02	-0.03	0.02	0.02	0.01	0.03	1.00	69
b_1	-0.2	0.00	0.01	0.01	0.01	0.00	0.01	1.00	92
b_2	-0.3	0.01	-0.02	0.02	0.03	0.00	0.03	1.00	87
c_1	0.3	-0.05	-0.16	0.06	0.06	0.01	0.07	0.99	89
c_2	-0.3	0.04	-0.13	0.10	0.09	0.01	0.10	0.77	94
d_1	0.5	-0.07	-0.14	0.03	0.03	0.01	0.08	1.00	21
d_2	-0.4	0.04	-0.11	0.04	0.04	0.00	0.06	1.00	84
$\sigma^2_{\zeta_1}$	2.0	-0.31	-0.15	0.06	0.08	0.00	0.32	1.00	2
$\sigma_{\zeta_{12}}$	0.5	-0.25	-0.50	0.07	0.07	0.00	0.26	0.94	6
$\sigma^2_{\zeta_2}$	6.0	-1.17	-0.19	0.18	0.39	0.01	1.23	1.00	7
μ_1	3.0	0.34	0.11	0.46	0.45	0.06	0.56	1.00	82
μ_2	3.0	0.77	0.26	0.90	0.88	0.10	1.17	0.96	79
μ_3	3.0	0.40	0.13	0.45	0.44	0.05	0.60	1.00	78
μ_4	3.0	-0.83	-0.28	0.57	0.56	0.04	1.00	0.95	62
μ_5	3.0	-1.77	-0.59	1.12	1.10	0.06	2.08	0.26	58
μ_6	3.0	-0.95	-0.32	0.55	0.56	0.02	1.10	0.94	53
λ_1	2.0	-0.04	-0.02	0.02	0.02	0.01	0.05	1.00	44
λ_2	1.0	-0.03	-0.03	0.01	0.01	0.00	0.03	1.00	50
λ_3	2.0	-0.04	-0.02	0.02	0.02	0.01	0.04	1.00	45
λ_4	1.0	-0.04	-0.04	0.01	0.02	0.01	0.05	1.00	16
$\sigma^2_{\epsilon_1}$	1.0	-0.13	-0.13	0.04	0.05	0.01	0.14	1.00	17
$\sigma^2_{\epsilon_2}$	1.0	0.56	0.56	0.14	0.14	0.07	0.58	1.00	1
$\sigma^2_{\epsilon_3}$	1.0	0.54	0.54	0.10	0.14	0.07	0.56	1.00	0
$\sigma^2_{\epsilon_4}$	1.0	-0.19	-0.19	0.06	0.07	0.03	0.20	1.00	22
$\sigma^2_{\epsilon_5}$	1.0	0.86	0.86	0.25	0.27	0.17	0.90	1.00	11
$\sigma^2_{\epsilon_6}$	1.0	1.67	1.67	0.30	0.89	0.27	1.89	1.00	0

Table S9

Simulation results based on LD and data with (1) a low level of autocorrelation and (2) factor-dependent missingness

	True	Bias	RBias	SE	MCSE	dSEfull	RMSE	Power	CR(%)
a_1	0.5	-0.14	-0.28	0.02	0.03	0.01	0.14	1.00	0
a_2	0.5	-0.16	-0.31	0.03	0.03	0.01	0.16	1.00	0
b_1	-0.2	0.02	-0.10	0.02	0.02	0.01	0.03	1.00	79
b_2	-0.3	0.05	-0.16	0.04	0.04	0.02	0.06	1.00	76
c_1	0.3	0.07	0.23	0.09	0.09	0.04	0.11	0.98	88
c_2	-0.3	-0.11	0.38	0.14	0.14	0.05	0.18	0.84	88
d_1	0.5	0.03	0.05	0.04	0.04	0.02	0.05	1.00	91
d_2	-0.4	-0.08	0.19	0.06	0.06	0.02	0.10	1.00	78
$\sigma^2_{\zeta_1}$	2.0	0.53	0.27	0.13	0.13	0.06	0.55	1.00	0
$\sigma_{\zeta_{12}}$	0.5	-0.76	-1.53	0.12	0.13	0.05	0.77	0.55	0
$\sigma^2_{\zeta_2}$	6.0	0.51	0.09	0.29	0.31	0.12	0.60	1.00	60
μ_1	3.0	0.36	0.12	0.11	0.12	0.00	0.38	1.00	14
μ_2	3.0	0.76	0.25	0.22	0.24	0.01	0.79	1.00	10
μ_3	3.0	0.36	0.12	0.11	0.12	0.00	0.38	1.00	14
μ_4	3.0	-0.60	-0.20	0.16	0.18	0.00	0.63	1.00	6
μ_5	3.0	-1.25	-0.42	0.32	0.35	0.00	1.29	1.00	5
μ_6	3.0	-0.60	-0.20	0.16	0.18	0.00	0.63	1.00	6
λ_1	2.0	0.00	0.00	0.04	0.04	0.02	0.04	1.00	96
λ_2	1.0	0.00	0.00	0.02	0.02	0.01	0.02	1.00	95
λ_3	2.0	-0.01	0.00	0.03	0.03	0.02	0.03	1.00	92
λ_4	1.0	0.00	0.00	0.02	0.02	0.01	0.02	1.00	96
$\sigma^2_{\epsilon_1}$	1.0	0.00	0.00	0.05	0.05	0.02	0.05	1.00	95
$\sigma^2_{\epsilon_2}$	1.0	0.00	0.00	0.14	0.14	0.06	0.14	1.00	96
$\sigma^2_{\epsilon_3}$	1.0	0.00	0.00	0.05	0.05	0.02	0.05	1.00	96
$\sigma^2_{\epsilon_4}$	1.0	0.00	0.00	0.06	0.06	0.03	0.06	1.00	93
$\sigma^2_{\epsilon_5}$	1.0	0.00	0.00	0.16	0.16	0.07	0.16	1.00	94
$\sigma^2_{\epsilon_6}$	1.0	0.00	0.00	0.06	0.06	0.03	0.06	1.00	93

Table S10

Simulation results based on PMI-MV and data with (1) a low level of autocorrelation and (2) factor-dependent missingness

	True	Bias	RBias	SE	MCSE	dSEfull	RMSE	Power	CR(%)
a_1	0.5	-0.01	-0.03	0.02	0.02	0.01	0.02	1.00	88
a_2	0.5	-0.03	-0.07	0.02	0.02	0.00	0.04	1.00	54
b_1	-0.2	0.00	0.00	0.01	0.01	0.00	0.01	1.00	96
b_2	-0.3	0.03	-0.09	0.03	0.03	0.01	0.04	1.00	81
c_1	0.3	-0.01	-0.03	0.07	0.06	0.02	0.06	0.99	96
c_2	-0.3	-0.03	0.10	0.11	0.10	0.02	0.11	0.90	95
d_1	0.5	0.02	0.05	0.03	0.03	0.01	0.04	1.00	84
d_2	-0.4	-0.09	0.21	0.04	0.04	0.00	0.10	1.00	51
$\sigma^2_{\zeta_1}$	2.0	-0.08	-0.04	0.08	0.08	0.01	0.11	1.00	81
$\sigma_{\zeta_{12}}$	0.5	-0.13	-0.26	0.08	0.08	0.01	0.15	1.00	56
$\sigma^2_{\zeta_2}$	6.0	-0.65	-0.11	0.18	0.17	0.01	0.67	1.00	5
μ_1	3.0	0.03	0.01	0.12	0.12	0.01	0.13	1.00	94
μ_2	3.0	0.10	0.03	0.23	0.24	0.02	0.26	1.00	91
μ_3	3.0	0.03	0.01	0.12	0.12	0.01	0.12	1.00	94
μ_4	3.0	-0.27	-0.09	0.17	0.18	0.01	0.32	1.00	62
μ_5	3.0	-0.58	-0.19	0.33	0.35	0.01	0.68	1.00	57
μ_6	3.0	-0.27	-0.09	0.17	0.18	0.01	0.32	1.00	63
λ_1	2.0	0.00	0.00	0.03	0.03	0.01	0.03	1.00	95
λ_2	1.0	0.00	0.00	0.02	0.02	0.01	0.02	1.00	95
λ_3	2.0	-0.01	0.00	0.02	0.02	0.01	0.02	1.00	93
λ_4	1.0	0.00	0.00	0.01	0.01	0.00	0.01	1.00	95
$\sigma^2_{\epsilon_1}$	1.0	0.00	0.00	0.04	0.04	0.01	0.04	1.00	95
$\sigma^2_{\epsilon_2}$	1.0	0.00	0.00	0.10	0.10	0.02	0.10	1.00	96
$\sigma^2_{\epsilon_3}$	1.0	0.00	0.00	0.04	0.04	0.01	0.04	1.00	95
$\sigma^2_{\epsilon_4}$	1.0	0.00	0.00	0.04	0.05	0.01	0.05	1.00	92
$\sigma^2_{\epsilon_5}$	1.0	0.00	0.00	0.12	0.12	0.03	0.12	1.00	93
$\sigma^2_{\epsilon_6}$	1.0	0.00	0.00	0.04	0.04	0.01	0.04	1.00	95

Table S11

Simulation results based on MI-MV and data with (1) a low level of autocorrelation and (2) factor-dependent missingness

	True	Bias	RBias	SE	MCSE	dSEfull	RMSE	Power	CR(%)
a_1	0.5	-0.03	-0.06	0.02	0.01	0.01	0.03	1.00	60
a_2	0.5	-0.04	-0.08	0.02	0.02	0.00	0.04	1.00	42
b_1	-0.2	0.01	-0.04	0.01	0.01	0.00	0.01	1.00	93
b_2	-0.3	0.01	-0.02	0.03	0.03	0.01	0.03	1.00	94
c_1	0.3	-0.02	-0.06	0.07	0.07	0.02	0.07	0.98	94
c_2	-0.3	0.04	-0.14	0.11	0.11	0.02	0.12	0.63	92
d_1	0.5	-0.01	-0.02	0.03	0.03	0.01	0.03	1.00	95
d_2	-0.4	0.02	-0.06	0.05	0.05	0.01	0.05	1.00	92
$\sigma^2_{\zeta_1}$	2.0	0.19	0.09	0.09	0.09	0.02	0.21	1.00	48
$\sigma_{\zeta_{12}}$	0.5	-0.21	-0.42	0.09	0.08	0.02	0.23	0.93	31
$\sigma^2_{\zeta_2}$	6.0	0.25	0.04	0.24	0.26	0.07	0.36	1.00	80
μ_1	3.0	0.00	0.00	0.12	0.13	0.01	0.13	1.00	95
μ_2	3.0	0.06	0.02	0.24	0.25	0.03	0.25	1.00	93
μ_3	3.0	0.00	0.00	0.12	0.13	0.01	0.13	1.00	95
μ_4	3.0	-0.05	-0.02	0.18	0.19	0.02	0.20	1.00	92
μ_5	3.0	-0.17	-0.06	0.35	0.38	0.03	0.42	1.00	91
μ_6	3.0	-0.05	-0.02	0.18	0.19	0.02	0.20	1.00	92
λ_1	2.0	-0.01	-0.01	0.03	0.03	0.01	0.03	1.00	91
λ_2	1.0	0.00	0.00	0.02	0.02	0.01	0.02	1.00	93
λ_3	2.0	-0.01	-0.01	0.02	0.03	0.01	0.03	1.00	87
λ_4	1.0	0.00	0.00	0.01	0.02	0.00	0.02	1.00	90
$\sigma^2_{\epsilon_1}$	1.0	0.00	0.00	0.04	0.04	0.01	0.04	1.00	94
$\sigma^2_{\epsilon_2}$	1.0	0.07	0.07	0.11	0.10	0.03	0.12	1.00	92
$\sigma^2_{\epsilon_3}$	1.0	0.00	0.00	0.04	0.04	0.01	0.04	1.00	92
$\sigma^2_{\epsilon_4}$	1.0	0.01	0.01	0.05	0.05	0.02	0.05	1.00	90
$\sigma^2_{\epsilon_5}$	1.0	0.07	0.07	0.13	0.14	0.04	0.15	1.00	89
$\sigma^2_{\epsilon_6}$	1.0	0.00	0.00	0.05	0.05	0.02	0.05	1.00	93

Table S12

Simulation results based on MI-FS and data with (1) a low level of autocorrelation and (2) factor-dependent missingness

	True	Bias	RBias	SE	MCSE	dSEfull	RMSE	Power	CR(%)
a_1	0.5	0.01	0.02	0.01	0.02	0.00	0.02	1.00	86
a_2	0.5	-0.01	-0.02	0.02	0.02	0.00	0.02	1.00	90
b_1	-0.2	-0.01	0.03	0.01	0.01	0.00	0.01	1.00	87
b_2	-0.3	0.03	-0.10	0.02	0.03	0.00	0.04	1.00	68
c_1	0.3	-0.04	-0.14	0.06	0.06	0.01	0.07	0.99	90
c_2	-0.3	0.01	-0.04	0.10	0.10	0.01	0.10	0.84	94
d_1	0.5	-0.05	-0.09	0.02	0.03	0.00	0.05	1.00	56
d_2	-0.4	-0.01	0.03	0.04	0.04	0.00	0.04	1.00	92
$\sigma^2_{\zeta_1}$	2.0	-0.20	-0.10	0.07	0.09	0.00	0.22	1.00	26
$\sigma_{\zeta_{12}}$	0.5	-0.16	-0.32	0.06	0.08	-0.01	0.18	0.99	32
$\sigma^2_{\zeta_2}$	6.0	-1.15	-0.19	0.16	0.17	-0.01	1.16	1.00	0
μ_1	3.0	0.02	0.01	0.12	0.12	0.01	0.12	1.00	95
μ_2	3.0	0.09	0.03	0.24	0.24	0.03	0.26	1.00	92
μ_3	3.0	0.03	0.01	0.12	0.12	0.01	0.12	1.00	94
μ_4	3.0	-0.29	-0.10	0.17	0.18	0.01	0.34	1.00	57
μ_5	3.0	-0.63	-0.21	0.33	0.35	0.01	0.72	1.00	51
μ_6	3.0	-0.31	-0.10	0.16	0.18	0.00	0.36	1.00	50
λ_1	2.0	-0.03	-0.01	0.03	0.03	0.01	0.04	1.00	75
λ_2	1.0	-0.03	-0.03	0.02	0.03	0.01	0.04	1.00	47
λ_3	2.0	-0.03	-0.01	0.02	0.02	0.01	0.04	1.00	67
λ_4	1.0	-0.05	-0.05	0.01	0.01	0.00	0.05	1.00	10
$\sigma^2_{\epsilon_1}$	1.0	-0.17	-0.17	0.03	0.04	0.00	0.18	1.00	1
$\sigma^2_{\epsilon_2}$	1.0	0.02	0.02	0.09	0.25	0.01	0.25	1.00	30
$\sigma^2_{\epsilon_3}$	1.0	0.08	0.08	0.05	0.22	0.02	0.24	1.00	0
$\sigma^2_{\epsilon_4}$	1.0	-0.22	-0.22	0.04	0.05	0.01	0.23	1.00	1
$\sigma^2_{\epsilon_5}$	1.0	0.47	0.47	0.15	0.14	0.06	0.49	1.00	15
$\sigma^2_{\epsilon_6}$	1.0	0.57	0.57	0.10	0.11	0.07	0.58	1.00	0

Table S13

Simulation results based on LD and data with (1) a high level of autocorrelation and (2) factor-dependent missingness

	True	Bias	RBias	SE	MCSE	dSEfull	RMSE	Power	CR(%)
a_1	0.7	-0.10	-0.14	0.02	0.02	0.01	0.10	1.00	0
a_2	0.7	-0.13	-0.18	0.03	0.03	0.02	0.13	1.00	0
b_1	-0.2	-0.05	0.24	0.02	0.02	0.01	0.05	1.00	15
b_2	-0.3	-0.06	0.21	0.03	0.03	0.01	0.07	1.00	51
c_1	0.3	0.14	0.46	0.09	0.10	0.04	0.17	0.99	69
c_2	-0.3	-0.19	0.64	0.14	0.15	0.05	0.24	0.91	73
d_1	0.5	0.01	0.03	0.05	0.04	0.03	0.05	1.00	96
d_2	-0.4	-0.04	0.10	0.07	0.07	0.03	0.08	1.00	91
$\sigma^2_{\zeta_1}$	2.0	1.13	0.56	0.14	0.16	0.08	1.14	1.00	0
$\sigma_{\zeta_{12}}$	0.5	-1.21	-2.41	0.15	0.18	0.08	1.22	0.99	0
$\sigma^2_{\zeta_2}$	6.0	1.81	0.30	0.34	0.38	0.17	1.84	1.00	0
μ_1	3.0	1.29	0.43	0.41	0.46	0.01	1.37	1.00	15
μ_2	3.0	2.62	0.87	0.81	0.91	0.01	2.77	1.00	14
μ_3	3.0	1.29	0.43	0.41	0.46	0.01	1.37	1.00	14
μ_4	3.0	-1.81	-0.60	0.51	0.56	-0.02	1.89	0.63	9
μ_5	3.0	-3.67	-1.22	1.02	1.12	-0.04	3.83	0.15	8
μ_6	3.0	-1.81	-0.60	0.51	0.56	-0.02	1.90	0.63	10
λ_1	2.0	-0.01	0.00	0.02	0.02	0.01	0.02	1.00	94
λ_2	1.0	0.00	0.00	0.01	0.01	0.00	0.01	1.00	94
λ_3	2.0	-0.01	0.00	0.02	0.02	0.01	0.02	1.00	93
λ_4	1.0	0.00	0.00	0.01	0.01	0.01	0.01	1.00	95
$\sigma^2_{\epsilon_1}$	1.0	0.00	0.00	0.05	0.05	0.02	0.05	1.00	97
$\sigma^2_{\epsilon_2}$	1.0	0.02	0.02	0.14	0.13	0.07	0.14	1.00	96
$\sigma^2_{\epsilon_3}$	1.0	0.00	0.00	0.05	0.05	0.02	0.05	1.00	96
$\sigma^2_{\epsilon_4}$	1.0	0.00	0.00	0.06	0.06	0.03	0.06	1.00	95
$\sigma^2_{\epsilon_5}$	1.0	0.00	0.00	0.15	0.15	0.07	0.15	1.00	96
$\sigma^2_{\epsilon_6}$	1.0	0.00	0.00	0.06	0.06	0.03	0.06	1.00	94

Table S14

Simulation results based on PMI-MV and data with (1) a high level of autocorrelation and (2) factor-dependent missingness

	True	Bias	RBias	SE	MCSE	dSEfull	RMSE	Power	CR(%)
a_1	0.7	0.00	0.00	0.01	0.01	0.00	0.01	1.00	96
a_2	0.7	-0.02	-0.03	0.02	0.02	0.01	0.03	1.00	67
b_1	-0.2	-0.01	0.03	0.01	0.01	0.00	0.01	1.00	90
b_2	-0.3	0.00	-0.01	0.02	0.02	0.00	0.02	1.00	94
c_1	0.3	-0.02	-0.08	0.07	0.06	0.02	0.07	0.99	96
c_2	-0.3	0.02	-0.05	0.11	0.10	0.02	0.11	0.77	94
d_1	0.5	0.00	0.01	0.03	0.03	0.01	0.03	1.00	96
d_2	-0.4	-0.04	0.10	0.04	0.04	0.00	0.06	1.00	86
$\sigma^2_{\zeta_1}$	2.0	-0.06	-0.03	0.07	0.07	0.01	0.09	1.00	85
$\sigma_{\zeta_{12}}$	0.5	-0.08	-0.17	0.08	0.08	0.01	0.12	1.00	81
$\sigma^2_{\zeta_2}$	6.0	-0.47	-0.08	0.18	0.18	0.01	0.51	1.00	27
μ_1	3.0	0.07	0.02	0.46	0.44	0.06	0.45	1.00	93
μ_2	3.0	0.19	0.06	0.91	0.89	0.11	0.91	0.94	92
μ_3	3.0	0.07	0.02	0.46	0.44	0.06	0.45	1.00	93
μ_4	3.0	-0.49	-0.16	0.58	0.55	0.05	0.74	0.98	82
μ_5	3.0	-1.04	-0.35	1.15	1.11	0.09	1.52	0.47	81
μ_6	3.0	-0.50	-0.17	0.58	0.55	0.05	0.74	0.98	82
λ_1	2.0	-0.01	0.00	0.02	0.02	0.01	0.02	1.00	92
λ_2	1.0	0.00	0.00	0.01	0.01	0.00	0.01	1.00	93
λ_3	2.0	-0.01	0.00	0.01	0.01	0.00	0.01	1.00	94
λ_4	1.0	0.00	0.00	0.01	0.01	0.01	0.01	1.00	96
$\sigma^2_{\epsilon_1}$	1.0	0.00	0.00	0.04	0.04	0.01	0.04	1.00	96
$\sigma^2_{\epsilon_2}$	1.0	0.01	0.01	0.09	0.09	0.02	0.09	1.00	95
$\sigma^2_{\epsilon_3}$	1.0	0.00	0.00	0.04	0.04	0.01	0.04	1.00	95
$\sigma^2_{\epsilon_4}$	1.0	0.00	0.00	0.04	0.04	0.01	0.04	1.00	94
$\sigma^2_{\epsilon_5}$	1.0	0.01	0.01	0.11	0.11	0.03	0.11	1.00	95
$\sigma^2_{\epsilon_6}$	1.0	0.00	0.00	0.04	0.04	0.01	0.04	1.00	94

Table S15

Simulation results based on MI-MV and data with (1) a high level of autocorrelation and (2) factor-dependent missingness

	True	Bias	RBias	SE	MCSE	dSEfull	RMSE	Power	CR(%)
a_1	0.7	-0.04	-0.06	0.01	0.01	0.00	0.04	1.00	16
a_2	0.7	-0.05	-0.07	0.02	0.02	0.01	0.05	1.00	9
b_1	-0.2	-0.01	0.07	0.01	0.01	0.00	0.02	1.00	83
b_2	-0.3	-0.02	0.07	0.02	0.02	0.00	0.03	1.00	87
c_1	0.3	0.00	0.00	0.08	0.07	0.03	0.07	0.98	96
c_2	-0.3	0.01	-0.03	0.12	0.11	0.03	0.11	0.70	94
d_1	0.5	-0.01	-0.02	0.03	0.03	0.01	0.03	1.00	95
d_2	-0.4	0.02	-0.05	0.05	0.05	0.01	0.05	1.00	94
$\sigma^2_{\zeta_1}$	2.0	0.68	0.34	0.12	0.17	0.06	0.70	1.00	0
$\sigma_{\zeta_{12}}$	0.5	-0.63	-1.26	0.11	0.15	0.04	0.65	0.25	0
$\sigma^2_{\zeta_2}$	6.0	0.71	0.12	0.26	0.29	0.09	0.77	1.00	24
μ_1	3.0	0.42	0.14	0.42	0.40	0.02	0.58	0.99	81
μ_2	3.0	0.91	0.30	0.84	0.79	0.04	1.21	0.99	77
μ_3	3.0	0.41	0.14	0.42	0.40	0.02	0.57	0.99	81
μ_4	3.0	-0.68	-0.23	0.54	0.51	0.01	0.85	0.97	75
μ_5	3.0	-1.44	-0.48	1.08	1.03	0.02	1.76	0.32	71
μ_6	3.0	-0.68	-0.23	0.54	0.52	0.01	0.85	0.97	73
λ_1	2.0	-0.01	-0.01	0.02	0.03	0.01	0.03	1.00	83
λ_2	1.0	0.00	0.00	0.01	0.02	0.00	0.02	1.00	87
λ_3	2.0	-0.01	0.00	0.02	0.02	0.01	0.03	1.00	82
λ_4	1.0	0.00	0.00	0.01	0.01	0.01	0.01	1.00	86
$\sigma^2_{\epsilon_1}$	1.0	0.00	0.00	0.05	0.05	0.02	0.05	1.00	93
$\sigma^2_{\epsilon_2}$	1.0	0.15	0.15	0.11	0.11	0.04	0.18	1.00	76
$\sigma^2_{\epsilon_3}$	1.0	0.00	0.00	0.05	0.05	0.02	0.05	1.00	94
$\sigma^2_{\epsilon_4}$	1.0	0.01	0.01	0.05	0.06	0.02	0.06	1.00	89
$\sigma^2_{\epsilon_5}$	1.0	0.14	0.14	0.14	0.15	0.06	0.21	1.00	84
$\sigma^2_{\epsilon_6}$	1.0	0.02	0.02	0.06	0.06	0.03	0.06	1.00	92

Table S16

Simulation results based on MI-FS and data with (1) a high level of autocorrelation and (2) factor-dependent missingness

	True	Bias	RBias	SE	MCSE	dSEfull	RMSE	Power	CR(%)
a_1	0.7	0.01	0.01	0.01	0.01	0.00	0.02	1.00	86
a_2	0.7	-0.01	-0.02	0.01	0.02	0.00	0.02	1.00	84
b_1	-0.2	0.00	0.02	0.01	0.01	0.00	0.01	1.00	92
b_2	-0.3	0.01	-0.05	0.02	0.02	0.00	0.03	1.00	81
c_1	0.3	-0.05	-0.18	0.06	0.06	0.01	0.08	0.98	87
c_2	-0.3	0.04	-0.13	0.10	0.09	0.01	0.10	0.77	95
d_1	0.5	-0.08	-0.16	0.03	0.02	0.01	0.09	1.00	9
d_2	-0.4	0.04	-0.11	0.04	0.04	0.00	0.06	1.00	81
$\sigma^2_{\zeta_1}$	2.0	-0.25	-0.12	0.07	0.08	0.01	0.26	1.00	9
$\sigma_{\zeta_{12}}$	0.5	-0.24	-0.48	0.07	0.08	0.00	0.25	0.95	8
$\sigma^2_{\zeta_2}$	6.0	-1.10	-0.18	0.17	0.24	0.00	1.13	1.00	1
μ_1	3.0	0.13	0.04	0.47	0.46	0.07	0.47	1.00	89
μ_2	3.0	0.32	0.11	0.94	0.91	0.14	0.96	0.90	88
μ_3	3.0	0.18	0.06	0.46	0.45	0.06	0.48	1.00	87
μ_4	3.0	-0.63	-0.21	0.59	0.56	0.06	0.84	0.95	75
μ_5	3.0	-1.28	-0.43	1.17	1.11	0.11	1.69	0.38	75
μ_6	3.0	-0.72	-0.24	0.57	0.55	0.04	0.90	0.95	69
λ_1	2.0	-0.01	-0.01	0.02	0.02	0.01	0.02	1.00	85
λ_2	1.0	-0.02	-0.02	0.01	0.01	0.00	0.02	1.00	61
λ_3	2.0	-0.01	0.00	0.02	0.02	0.01	0.02	1.00	92
λ_4	1.0	-0.03	-0.03	0.01	0.01	0.01	0.04	1.00	29
$\sigma^2_{\epsilon_1}$	1.0	-0.14	-0.14	0.04	0.04	0.01	0.15	1.00	11
$\sigma^2_{\epsilon_2}$	1.0	0.44	0.44	0.12	0.11	0.05	0.45	1.00	4
$\sigma^2_{\epsilon_3}$	1.0	0.46	0.46	0.09	0.09	0.06	0.47	1.00	0
$\sigma^2_{\epsilon_4}$	1.0	-0.19	-0.19	0.06	0.06	0.03	0.20	1.00	14
$\sigma^2_{\epsilon_5}$	1.0	0.70	0.70	0.21	0.19	0.13	0.73	1.00	10
$\sigma^2_{\epsilon_6}$	1.0	1.35	1.35	0.22	0.36	0.19	1.40	1.00	0

Table S17

Simulation results based on MI without manifest variables and data with (1) a high level of autocorrelation and (2) factor-dependent missingness

	True	Bias	RBias	SE	MCSE	dSEfull	RMSE	Power	CR(%)
a_1	0.7	0.01	0.02	0.01	0.01	0.00	0.02	1.00	79
a_2	0.7	0.01	0.01	0.01	0.02	0.00	0.02	1.00	83
b_1	-0.2	-0.01	0.03	0.01	0.01	0.00	0.01	1.00	90
b_2	-0.3	0.03	-0.09	0.02	0.02	0.00	0.04	1.00	58
c_1	0.3	-0.05	-0.18	0.06	0.06	0.01	0.08	0.96	90
c_2	-0.3	0.03	-0.10	0.10	0.10	0.01	0.10	0.75	92
d_1	0.5	-0.05	-0.10	0.02	0.03	0.00	0.06	1.00	50
d_2	-0.4	0.03	-0.09	0.04	0.04	0.00	0.05	1.00	90
$\sigma^2_{\zeta_1}$	2.0	-0.42	-0.21	0.06	0.07	0.00	0.42	1.00	0
$\sigma_{\zeta_{12}}$	0.5	-0.21	-0.43	0.06	0.09	-0.01	0.23	0.98	19
$\sigma^2_{\zeta_2}$	6.0	-1.49	-0.25	0.14	0.20	-0.03	1.51	1.00	0
μ_1	3.0	-0.18	-0.06	0.55	0.42	0.15	0.45	0.98	100
μ_2	3.0	0.11	0.04	1.07	0.81	0.27	0.81	0.85	96
μ_3	3.0	-0.18	-0.06	0.56	0.41	0.16	0.45	0.98	98
μ_4	3.0	-0.27	-0.09	0.69	0.54	0.16	0.60	0.94	92
μ_5	3.0	-1.15	-0.38	1.32	1.08	0.26	1.57	0.44	79
μ_6	3.0	-0.28	-0.09	0.69	0.55	0.16	0.61	0.94	88
λ_1	2.0	-0.07	-0.04	0.02	0.02	0.01	0.08	1.00	8
λ_2	1.0	0.00	0.00	0.01	0.01	0.00	0.01	1.00	92
λ_3	2.0	-0.10	-0.05	0.02	0.03	0.01	0.10	1.00	4
λ_4	1.0	0.00	0.00	0.01	0.01	0.01	0.01	1.00	92
$\sigma^2_{\epsilon_1}$	1.0	-0.09	-0.09	0.04	0.03	0.01	0.09	1.00	40
$\sigma^2_{\epsilon_2}$	1.0	3.04	3.04	0.24	0.24	0.17	3.05	1.00	0
$\sigma^2_{\epsilon_3}$	1.0	-0.08	-0.08	0.04	0.04	0.01	0.09	1.00	48
$\sigma^2_{\epsilon_4}$	1.0	-0.12	-0.12	0.05	0.05	0.02	0.13	1.00	29
$\sigma^2_{\epsilon_5}$	1.0	8.16	8.16	0.50	0.66	0.42	8.18	1.00	0
$\sigma^2_{\epsilon_6}$	1.0	-0.13	-0.13	0.05	0.05	0.02	0.14	1.00	17

Table S18

Simulation results based on PMI-MV and data with (1) a low level of autocorrelation and (2) scattered missingness

	True	Bias	RBias	SE	MCSE	dSEfull	RMSE	Power	CR(%)
a_1	0.5	0.00	0.00	0.02	0.02	0.01	0.02	1.00	94
a_2	0.5	0.00	0.00	0.02	0.02	0.00	0.02	1.00	94
b_1	-0.2	0.00	0.00	0.01	0.01	0.00	0.01	1.00	98
b_2	-0.3	0.00	-0.01	0.03	0.03	0.01	0.03	1.00	98
c_1	0.3	-0.01	-0.03	0.07	0.07	0.02	0.07	0.98	94
c_2	-0.3	0.00	0.00	0.11	0.10	0.02	0.10	0.81	98
d_1	0.5	0.00	0.00	0.03	0.03	0.01	0.03	1.00	96
d_2	-0.4	0.00	-0.01	0.04	0.05	0.00	0.05	1.00	95
$\sigma^2_{\zeta_1}$	2.0	0.00	0.00	0.08	0.08	0.01	0.08	1.00	91
$\sigma_{\zeta_{12}}$	0.5	0.00	0.00	0.08	0.08	0.01	0.08	1.00	92
$\sigma^2_{\zeta_2}$	6.0	0.02	0.00	0.19	0.18	0.02	0.18	1.00	97
μ_1	3.0	0.01	0.00	0.12	0.13	0.01	0.13	1.00	94
μ_2	3.0	0.02	0.01	0.24	0.26	0.03	0.26	1.00	93
μ_3	3.0	0.01	0.00	0.12	0.13	0.01	0.13	1.00	95
μ_4	3.0	-0.03	-0.01	0.18	0.19	0.02	0.19	1.00	94
μ_5	3.0	-0.05	-0.02	0.36	0.38	0.04	0.38	1.00	95
μ_6	3.0	-0.03	-0.01	0.18	0.19	0.02	0.19	1.00	97
λ_1	2.0	0.00	0.00	0.03	0.03	0.01	0.03	1.00	91
λ_2	1.0	0.00	0.00	0.02	0.02	0.01	0.02	1.00	96
λ_3	2.0	0.00	0.00	0.02	0.02	0.01	0.02	1.00	98
λ_4	1.0	0.00	0.00	0.01	0.01	0.00	0.01	1.00	95
$\sigma^2_{\epsilon_1}$	1.0	-0.01	-0.01	0.04	0.04	0.01	0.04	1.00	95
$\sigma^2_{\epsilon_2}$	1.0	0.00	0.00	0.11	0.11	0.03	0.11	1.00	94
$\sigma^2_{\epsilon_3}$	1.0	0.00	0.00	0.04	0.04	0.01	0.04	1.00	97
$\sigma^2_{\epsilon_4}$	1.0	0.00	0.00	0.05	0.05	0.02	0.05	1.00	94
$\sigma^2_{\epsilon_5}$	1.0	0.00	0.00	0.13	0.15	0.04	0.15	1.00	94
$\sigma^2_{\epsilon_6}$	1.0	0.00	0.00	0.05	0.05	0.02	0.05	1.00	95

Table S19

Simulation results based on MI-MV and data with (1) a low level of autocorrelation and (2) scattered missingness

	True	Bias	RBias	SE	MCSE	dSEfull	RMSE	Power	CR(%)
a_1	0.5	-0.02	-0.04	0.02	0.02	0.01	0.02	1.00	76
a_2	0.5	-0.02	-0.03	0.02	0.02	0.00	0.02	1.00	84
b_1	-0.2	0.00	-0.01	0.01	0.01	0.00	0.01	1.00	99
b_2	-0.3	0.01	-0.02	0.03	0.03	0.01	0.03	1.00	99
c_1	0.3	0.00	-0.01	0.07	0.07	0.02	0.07	0.97	94
c_2	-0.3	-0.01	0.02	0.12	0.11	0.03	0.10	0.77	98
d_1	0.5	0.00	0.01	0.03	0.03	0.01	0.03	1.00	95
d_2	-0.4	0.00	-0.01	0.04	0.05	0.00	0.05	1.00	94
$\sigma^2_{\zeta_1}$	2.0	0.11	0.05	0.08	0.09	0.01	0.14	1.00	71
$\sigma_{\zeta_{12}}$	0.5	-0.10	-0.21	0.08	0.08	0.01	0.13	1.00	79
$\sigma^2_{\zeta_2}$	6.0	0.20	0.03	0.20	0.17	0.03	0.26	1.00	87
μ_1	3.0	0.04	0.01	0.12	0.13	0.01	0.13	1.00	91
μ_2	3.0	0.07	0.02	0.23	0.25	0.02	0.26	1.00	94
μ_3	3.0	0.04	0.01	0.12	0.12	0.01	0.13	1.00	91
μ_4	3.0	-0.05	-0.02	0.18	0.19	0.02	0.19	1.00	95
μ_5	3.0	-0.10	-0.03	0.36	0.37	0.04	0.39	1.00	96
μ_6	3.0	-0.05	-0.02	0.18	0.18	0.02	0.19	1.00	96
λ_1	2.0	0.00	0.00	0.03	0.03	0.01	0.03	1.00	90
λ_2	1.0	0.00	0.00	0.02	0.02	0.01	0.02	1.00	93
λ_3	2.0	0.00	0.00	0.02	0.02	0.01	0.02	1.00	95
λ_4	1.0	0.00	0.00	0.01	0.01	0.00	0.01	1.00	96
$\sigma^2_{\epsilon_1}$	1.0	-0.01	-0.01	0.04	0.04	0.01	0.05	1.00	98
$\sigma^2_{\epsilon_2}$	1.0	0.07	0.07	0.12	0.12	0.04	0.13	1.00	88
$\sigma^2_{\epsilon_3}$	1.0	-0.01	-0.01	0.04	0.05	0.01	0.05	1.00	93
$\sigma^2_{\epsilon_4}$	1.0	0.00	0.00	0.05	0.05	0.02	0.05	1.00	94
$\sigma^2_{\epsilon_5}$	1.0	0.05	0.05	0.14	0.15	0.05	0.16	1.00	90
$\sigma^2_{\epsilon_6}$	1.0	0.00	0.00	0.05	0.05	0.02	0.05	1.00	90

Table S20

Simulation results based on MI-FS and data with (1) a low level of autocorrelation and (2) scattered missingness

	True	Bias	RBias	SE	MCSE	dSEfull	RMSE	Power	CR(%)
a_1	0.5	0.00	0.01	0.01	0.02	0.00	0.02	1.00	86
a_2	0.5	0.00	0.00	0.02	0.02	0.00	0.02	1.00	92
b_1	-0.2	0.00	0.01	0.01	0.01	0.00	0.01	1.00	94
b_2	-0.3	0.01	-0.03	0.02	0.03	0.00	0.03	1.00	92
c_1	0.3	-0.02	-0.07	0.07	0.06	0.02	0.06	1.00	97
c_2	-0.3	0.00	-0.01	0.11	0.11	0.02	0.11	0.79	94
d_1	0.5	-0.02	-0.05	0.03	0.03	0.01	0.04	1.00	85
d_2	-0.4	0.03	-0.07	0.04	0.04	0.00	0.05	1.00	91
$\sigma^2_{\zeta_1}$	2.0	0.03	0.01	0.07	0.10	0.00	0.10	1.00	82
$\sigma_{\zeta_{12}}$	0.5	0.00	0.00	0.07	0.08	0.00	0.08	1.00	91
$\sigma^2_{\zeta_2}$	6.0	-0.03	0.00	0.18	0.16	0.01	0.17	1.00	95
μ_1	3.0	0.03	0.01	0.12	0.12	0.01	0.12	1.00	94
μ_2	3.0	0.05	0.02	0.24	0.23	0.03	0.23	1.00	95
μ_3	3.0	0.03	0.01	0.12	0.11	0.01	0.12	1.00	94
μ_4	3.0	-0.03	-0.01	0.18	0.18	0.02	0.18	1.00	97
μ_5	3.0	-0.07	-0.02	0.36	0.36	0.04	0.37	1.00	97
μ_6	3.0	-0.04	-0.01	0.18	0.17	0.02	0.18	1.00	97
λ_1	2.0	-0.02	-0.01	0.02	0.03	0.00	0.03	1.00	79
λ_2	1.0	-0.01	-0.01	0.01	0.02	0.00	0.02	1.00	79
λ_3	2.0	-0.01	-0.01	0.02	0.02	0.01	0.02	1.00	85
λ_4	1.0	-0.01	-0.01	0.01	0.01	0.00	0.01	1.00	80
$\sigma^2_{\epsilon_1}$	1.0	-0.19	-0.19	0.03	0.06	0.00	0.20	1.00	5
$\sigma^2_{\epsilon_2}$	1.0	-0.16	-0.16	0.09	0.12	0.01	0.20	1.00	61
$\sigma^2_{\epsilon_3}$	1.0	-0.06	-0.06	0.04	0.09	0.01	0.11	1.00	74
$\sigma^2_{\epsilon_4}$	1.0	-0.18	-0.18	0.04	0.05	0.01	0.19	1.00	3
$\sigma^2_{\epsilon_5}$	1.0	-0.12	-0.12	0.10	0.15	0.01	0.19	1.00	68
$\sigma^2_{\epsilon_6}$	1.0	-0.03	-0.03	0.04	0.06	0.01	0.06	1.00	77

Table S21

Simulation results based on PMI-MV and data with (1) a low level of autocorrelation and (2) simultaneous missingness

	True	Bias	RBias	SE	MCSE	dSEfull	RMSE	Power	CR(%)
a_1	0.5	0.00	0.00	0.02	0.02	0.01	0.02	1.00	95
a_2	0.5	0.00	0.00	0.02	0.02	0.00	0.02	1.00	95
b_1	-0.2	0.00	0.00	0.01	0.01	0.00	0.01	1.00	97
b_2	-0.3	0.01	-0.04	0.03	0.03	0.01	0.03	1.00	91
c_1	0.3	-0.01	-0.03	0.07	0.08	0.02	0.08	0.94	91
c_2	-0.3	0.02	-0.05	0.13	0.11	0.04	0.11	0.63	94
d_1	0.5	0.00	0.00	0.03	0.03	0.01	0.03	1.00	95
d_2	-0.4	0.00	-0.01	0.05	0.05	0.01	0.05	1.00	95
$\sigma^2_{\zeta_1}$	2.0	0.01	0.00	0.08	0.08	0.01	0.08	1.00	93
$\sigma_{\zeta_{12}}$	0.5	-0.02	-0.03	0.10	0.11	0.03	0.11	0.99	92
$\sigma^2_{\zeta_2}$	6.0	0.01	0.00	0.21	0.19	0.04	0.19	1.00	95
μ_1	3.0	0.02	0.01	0.13	0.13	0.02	0.13	1.00	93
μ_2	3.0	0.04	0.01	0.26	0.25	0.05	0.26	1.00	95
μ_3	3.0	0.02	0.01	0.13	0.13	0.02	0.13	1.00	94
μ_4	3.0	-0.05	-0.02	0.20	0.19	0.04	0.20	1.00	95
μ_5	3.0	-0.11	-0.04	0.40	0.38	0.08	0.40	1.00	95
μ_6	3.0	-0.05	-0.02	0.20	0.19	0.04	0.20	1.00	95
λ_1	2.0	0.00	0.00	0.03	0.03	0.01	0.03	1.00	94
λ_2	1.0	0.00	0.00	0.02	0.02	0.01	0.02	1.00	91
λ_3	2.0	0.00	0.00	0.02	0.01	0.01	0.01	1.00	97
λ_4	1.0	0.00	0.00	0.01	0.01	0.00	0.01	1.00	98
$\sigma^2_{\epsilon_1}$	1.0	-0.01	-0.01	0.04	0.04	0.01	0.04	1.00	89
$\sigma^2_{\epsilon_2}$	1.0	0.00	0.00	0.10	0.10	0.02	0.10	1.00	97
$\sigma^2_{\epsilon_3}$	1.0	0.00	0.00	0.04	0.04	0.01	0.04	1.00	93
$\sigma^2_{\epsilon_4}$	1.0	0.00	0.00	0.04	0.05	0.01	0.05	1.00	92
$\sigma^2_{\epsilon_5}$	1.0	-0.01	-0.01	0.10	0.11	0.01	0.11	1.00	91
$\sigma^2_{\epsilon_6}$	1.0	0.00	0.00	0.04	0.04	0.01	0.04	1.00	93

Table S22

Simulation results based on MI-MV and data with (1) a low level of autocorrelation and (2) simultaneous missingness

	True	Bias	RBias	SE	MCSE	dSEfull	RMSE	Power	CR(%)
a_1	0.5	-0.08	-0.17	0.02	0.02	0.01	0.09	1.00	0
a_2	0.5	-0.12	-0.25	0.02	0.02	0.00	0.12	1.00	0
b_1	-0.2	0.03	-0.15	0.01	0.01	0.00	0.03	1.00	46
b_2	-0.3	0.02	-0.06	0.03	0.03	0.01	0.03	1.00	97
c_1	0.3	0.01	0.04	0.08	0.08	0.03	0.08	0.98	94
c_2	-0.3	-0.01	0.04	0.14	0.13	0.05	0.13	0.64	94
d_1	0.5	0.00	0.00	0.03	0.03	0.01	0.03	1.00	94
d_2	-0.4	0.01	-0.03	0.06	0.05	0.02	0.05	1.00	95
$\sigma^2_{\zeta_1}$	2.0	0.55	0.27	0.11	0.11	0.04	0.56	1.00	0
$\sigma_{\zeta_{12}}$	0.5	-0.66	-1.31	0.12	0.12	0.05	0.67	0.26	0
$\sigma^2_{\zeta_2}$	6.0	1.12	0.19	0.26	0.24	0.09	1.14	1.00	1
μ_1	3.0	0.14	0.05	0.11	0.11	0.00	0.18	1.00	74
μ_2	3.0	0.28	0.09	0.22	0.21	0.01	0.35	1.00	78
μ_3	3.0	0.14	0.05	0.11	0.11	0.00	0.18	1.00	78
μ_4	3.0	-0.20	-0.07	0.17	0.18	0.01	0.27	1.00	76
μ_5	3.0	-0.41	-0.14	0.33	0.36	0.01	0.54	1.00	76
μ_6	3.0	-0.20	-0.07	0.17	0.18	0.01	0.27	1.00	76
λ_1	2.0	0.00	0.00	0.03	0.03	0.01	0.03	1.00	95
λ_2	1.0	0.00	0.00	0.02	0.02	0.01	0.02	1.00	95
λ_3	2.0	0.00	0.00	0.02	0.01	0.01	0.01	1.00	96
λ_4	1.0	0.00	0.00	0.01	0.01	0.00	0.01	1.00	99
$\sigma^2_{\epsilon_1}$	1.0	-0.01	-0.01	0.04	0.04	0.01	0.04	1.00	94
$\sigma^2_{\epsilon_2}$	1.0	0.03	0.03	0.11	0.10	0.03	0.11	1.00	94
$\sigma^2_{\epsilon_3}$	1.0	0.00	0.00	0.04	0.04	0.01	0.04	1.00	94
$\sigma^2_{\epsilon_4}$	1.0	0.00	0.00	0.04	0.05	0.01	0.05	1.00	90
$\sigma^2_{\epsilon_5}$	1.0	0.00	0.00	0.11	0.11	0.02	0.11	1.00	92
$\sigma^2_{\epsilon_6}$	1.0	0.00	0.00	0.04	0.04	0.01	0.04	1.00	93

Table S23

Simulation results based on MI-FS and data with (1) a low level of autocorrelation and (2) simultaneous missingness

	True	Bias	RBias	SE	MCSE	dSEfull	RMSE	Power	CR(%)
a_1	0.5	-0.04	-0.08	0.02	0.02	0.01	0.04	1.00	40
a_2	0.5	-0.07	-0.14	0.02	0.02	0.00	0.07	1.00	4
b_1	-0.2	0.02	-0.08	0.01	0.01	0.00	0.02	1.00	73
b_2	-0.3	0.00	0.00	0.03	0.03	0.01	0.03	1.00	98
c_1	0.3	-0.01	-0.02	0.08	0.08	0.03	0.08	0.95	93
c_2	-0.3	0.01	-0.02	0.13	0.12	0.04	0.12	0.66	95
d_1	0.5	-0.01	-0.03	0.03	0.03	0.01	0.03	1.00	94
d_2	-0.4	0.01	-0.02	0.05	0.05	0.01	0.05	1.00	95
$\sigma^2_{\zeta_1}$	2.0	0.28	0.14	0.09	0.10	0.02	0.29	1.00	16
$\sigma_{\zeta_{12}}$	0.5	-0.39	-0.77	0.11	0.12	0.04	0.40	0.24	7
$\sigma^2_{\zeta_2}$	6.0	0.59	0.10	0.24	0.22	0.07	0.63	1.00	27
μ_1	3.0	0.10	0.03	0.12	0.12	0.01	0.16	1.00	84
μ_2	3.0	0.19	0.06	0.24	0.24	0.03	0.30	1.00	85
μ_3	3.0	0.10	0.03	0.12	0.12	0.01	0.15	1.00	86
μ_4	3.0	-0.15	-0.05	0.18	0.20	0.02	0.25	1.00	82
μ_5	3.0	-0.30	-0.10	0.35	0.40	0.03	0.50	1.00	81
μ_6	3.0	-0.15	-0.05	0.18	0.20	0.02	0.25	1.00	81
λ_1	2.0	-0.01	0.00	0.03	0.03	0.01	0.03	1.00	92
λ_2	1.0	0.00	0.00	0.02	0.02	0.01	0.02	1.00	91
λ_3	2.0	0.00	0.00	0.02	0.01	0.01	0.01	1.00	94
λ_4	1.0	0.00	0.00	0.01	0.01	0.00	0.01	1.00	98
$\sigma^2_{\epsilon_1}$	1.0	-0.01	-0.01	0.04	0.04	0.01	0.04	1.00	94
$\sigma^2_{\epsilon_2}$	1.0	0.02	0.02	0.10	0.10	0.02	0.10	1.00	94
$\sigma^2_{\epsilon_3}$	1.0	0.00	0.00	0.04	0.04	0.01	0.04	1.00	94
$\sigma^2_{\epsilon_4}$	1.0	0.00	0.00	0.04	0.05	0.01	0.05	1.00	89
$\sigma^2_{\epsilon_5}$	1.0	0.01	0.01	0.11	0.12	0.02	0.12	1.00	91
$\sigma^2_{\epsilon_6}$	1.0	0.00	0.00	0.04	0.04	0.01	0.04	1.00	95

Table S24

Simulation results based on PMI-MV and data with (1) a low level of autocorrelation; (2) factor-dependent missingness; and (3) null effects on certain parameters

	True	Bias	RBias	SE	MCSE	dSEfull	RMSE	Power	CR(%)
a_1	0.5	-0.01	-0.03	0.02	0.02	0.00	0.02	1	91
a_2	0.5	-0.04	-0.07	0.02	0.02	0.00	0.04	1	58
b_1	0.0	0.00	–	0.01	0.01	0.00	0.01	0.03	97
b_2	0.0	0.01	–	0.03	0.03	0.00	0.03	0.03	97
c_1	0.0	0.04	–	0.07	0.06	0.02	0.07	0.06	94
c_2	0.0	0.02	–	0.07	0.07	0.05	0.08	0.05	95
d_1	0.0	-0.11	–	0.11	0.10	0.02	0.15	0.14	86
d_2	0.0	-0.03	–	0.12	0.11	0.08	0.12	0.05	95
$\sigma^2_{\zeta_1}$	2.0	-0.09	-0.04	0.08	0.08	0.01	0.12	1	76
$\sigma_{\zeta_{12}}$	0.5	-0.11	-0.22	0.08	0.08	0.01	0.14	1	71
$\sigma^2_{\zeta_2}$	6.0	-0.61	-0.10	0.18	0.18	0.00	0.63	1	9
μ_1	3.0	0.07	0.02	0.09	0.08	0.01	0.11	1	90
μ_2	3.0	0.17	0.06	0.17	0.15	0.02	0.23	1	86
μ_3	3.0	0.07	0.02	0.09	0.08	0.01	0.11	1	89
μ_4	3.0	-0.24	-0.08	0.14	0.13	0.01	0.27	1	59
μ_5	3.0	-0.52	-0.17	0.27	0.26	0.02	0.58	1	51
μ_6	3.0	-0.23	-0.08	0.14	0.13	0.01	0.27	1	59
λ_1	2.0	-0.01	-0.01	0.04	0.04	0.01	0.04	1	96
λ_2	1.0	0.00	0.00	0.02	0.02	0.00	0.02	1	94
λ_3	2.0	-0.01	0.00	0.02	0.02	0.00	0.02	1	97
λ_4	1.0	0.00	0.00	0.04	0.04	0.01	0.04	1	96
$\sigma^2_{\epsilon_1}$	1.0	0.02	0.02	0.11	0.11	0.03	0.11	1	93
$\sigma^2_{\epsilon_2}$	1.0	0.00	0.00	0.04	0.04	0.01	0.04	1	95
$\sigma^2_{\epsilon_3}$	1.0	0.00	0.00	0.04	0.04	0.01	0.04	1	94
$\sigma^2_{\epsilon_4}$	1.0	-0.01	-0.01	0.12	0.12	0.03	0.12	1	95
$\sigma^2_{\epsilon_5}$	1.0	0.01	0.01	0.04	0.04	0.01	0.04	1	96
$\sigma^2_{\epsilon_6}$	1.0	0.01	0.01	0.04	0.04	0.01	0.04	1	97

Table S25

Simulation results based on MI-MV and data with (1) a low level of autocorrelation; (2) factor-dependent missingness; and (3) null effects on certain parameters

	True	Bias	RBias	SE	MCSE	dSEfull	RMSE	Power	CR(%)
a_1	0.5	-0.05	-0.1	0.02	0.02	0.00	0.05	1	27
a_2	0.5	-0.04	-0.08	0.02	0.02	0.00	0.05	1	42
b_1	0	0	–	0.01	0.01	0.00	0.01	0.03	97
b_2	0	0.01	–	0.03	0.03	0.00	0.03	0.01	99
c_1	0	0	–	0.06	0.06	0.01	0.06	0.06	94
c_2	0	0	–	0.03	0.03	0.01	0.03	0.07	93
d_1	0	-0.01	–	0.10	0.10	0.01	0.10	0.07	93
d_2	0	0.01	–	0.05	0.05	0.01	0.06	0.06	94
$\sigma^2_{\zeta_1}$	2	0.12	0.06	0.10	0.11	0.03	0.16	1	72
$\sigma_{\zeta_{12}}$	0.5	0.01	0.02	0.08	0.09	0.01	0.09	1	94
$\sigma^2_{\zeta_2}$	6	0.19	0.03	0.24	0.26	0.06	0.32	1	88
μ_1	3	0.01	0	0.08	0.08	0.00	0.08	1	95
μ_2	3	0.08	0.03	0.15	0.14	0.00	0.16	1	94
μ_3	3	0.01	0	0.08	0.07	0.00	0.08	1	97
μ_4	3	-0.02	-0.01	0.13	0.13	0.00	0.13	1	94
μ_5	3	-0.1	-0.03	0.25	0.25	0.00	0.27	1	94
μ_6	3	-0.02	-0.01	0.13	0.13	0.00	0.13	1	96
λ_1	2	-0.02	-0.01	0.04	0.04	0.01	0.04	1	83
λ_2	1	0	0	0.02	0.02	0.00	0.02	1	94
λ_3	2	-0.01	-0.01	0.03	0.02	0.01	0.03	1	93
λ_4	1	0	0	0.02	0.01	0.01	0.04	1	91
$\sigma^2_{\epsilon_1}$	1	0.07	0.07	0.12	0.10	0.04	0.12	1	94
$\sigma^2_{\epsilon_2}$	1	-0.01	-0.01	0.04	0.04	0.01	0.04	1	93
$\sigma^2_{\epsilon_3}$	1	0.01	0.01	0.05	0.05	0.02	0.05	1	93
$\sigma^2_{\epsilon_4}$	1	0.04	0.04	0.13	0.12	0.04	0.13	1	95
$\sigma^2_{\epsilon_5}$	1	0.01	0.01	0.05	0.05	0.02	0.05	1	91
$\sigma^2_{\epsilon_6}$	1	0.04	0.04	0.13	0.12	0.04	0.13	1	93

Table S26

Simulation results based on MI-FS and data with (1) a low level of autocorrelation; (2) factor-dependent missingness; and (3) null effects on certain parameters

	True	Bias	RBias	SE	MCSE	dSEfull	RMSE	Power	CR(%)
a_1	0.5	0.04	0.07	0.02	0.02	0.00	0.04	1.00	41
a_2	0.5	0.00	-0.01	0.02	0.02	0.00	0.02	1.00	89
b_1	0.0	0.00	–	0.01	0.01	0.00	0.01	0.06	94
b_2	0.0	0.01	–	0.03	0.03	0.00	0.04	0.07	93
c_1	0.0	0.03	–	0.06	0.05	0.01	0.06	0.07	93
c_2	0.0	-0.10	–	0.10	0.09	0.01	0.14	0.13	87
d_1	0.0	0.02	–	0.06	0.06	0.04	0.07	0.08	92
d_2	0.0	-0.03	–	0.11	0.10	0.07	0.11	0.08	92
$\sigma^2_{\zeta_1}$	2.0	-0.36	-0.18	0.06	0.09	-0.01	0.37	1.00	4
$\sigma_{\zeta_{12}}$	0.5	-0.12	-0.24	0.06	0.08	-0.01	0.14	1.00	47
$\sigma^2_{\zeta_2}$	6.0	-1.12	-0.19	0.16	0.17	-0.02	1.13	1.00	0
μ_1	3.0	0.07	0.02	0.09	0.08	0.01	0.11	1.00	89
μ_2	3.0	0.19	0.06	0.16	0.15	0.01	0.24	1.00	82
μ_3	3.0	0.07	0.02	0.08	0.08	0.00	0.10	1.00	90
μ_4	3.0	-0.25	-0.08	0.14	0.13	0.01	0.28	1.00	53
μ_5	3.0	-0.53	-0.18	0.27	0.25	0.02	0.58	1.00	52
μ_6	3.0	-0.25	-0.08	0.13	0.13	0.00	0.28	1.00	52
λ_1	2.0	-0.03	-0.02	0.03	0.03	0.00	0.05	1.00	75
λ_2	1.0	-0.01	-0.01	0.02	0.03	0.00	0.03	1.00	83
λ_3	2.0	-0.03	-0.02	0.02	0.02	0.00	0.04	1.00	69
λ_4	1.0	-0.06	-0.06	0.02	0.01	0.01	0.06	1.00	1
$\sigma^2_{\epsilon_1}$	1.0	-0.17	-0.17	0.03	0.04	0.00	0.18	1.00	0
$\sigma^2_{\epsilon_2}$	1.0	-0.30	-0.30	0.07	0.12	-0.01	0.33	1.00	7
$\sigma^2_{\epsilon_3}$	1.0	-0.15	-0.15	0.03	0.12	0.00	0.19	1.00	0
$\sigma^2_{\epsilon_4}$	1.0	-0.22	-0.22	0.05	0.05	0.02	0.23	1.00	1
$\sigma^2_{\epsilon_5}$	1.0	0.46	0.46	0.17	0.15	0.08	0.48	1.00	22
$\sigma^2_{\epsilon_6}$	1.0	0.64	0.64	0.12	0.11	0.09	0.65	1.00	0
